# Supplementary material for: Formation of supramolecular channels by reversible unwinding-rewinding of bis(indole) double helix via ion coordination
Source: Nat Commun. 2022 Oct 31;13:6507. doi: 10.1038/s41467-022-34159-y (PMC9622825; doi:10.1038/s41467-022-34159-y)
Supplement: Supplementary file 1 — Supplementary Information [file 41467_2022_34159_MOESM1_ESM.pdf]

Supplementary Information

Formation of supramolecular channels by reversible unwinding-rewinding of bis(indole) double helix via ion coordination

Debashis Mondal[1], Manzoor Ahmad[1], Bijoy Dey[2], Abhishek Mondal[1] & Pinaki Talukdar[1]*

[1] Department of Chemistry, Indian Institute of Science Education and Research Pune, Pune 411008, India. E-mail: ptalukdar@iiserpune.ac.in

[2] Department of Chemistry, Tata Institute of Fundamental Research, 36/P, Gopanpally Village, Hyderabad, 500046, India

Table of Contents

Contents	Page Number
Supplementary Methods	S2
Synthesis of Compounds	S3
Crystallographic Measurement and Analysis	S14
ESI-MS Spectra of **1a** and **1b**	S18
Solution Phase NMR Studies	S20
Field Emission Scanning Electron Microscopy (FESEM)	S25
Atomic Force Microscopy (AFM)	S26
High-Resolution Transmission Electron Microscopy (HRTEM)	S27
ESI-MS Spectrum of Cl^- Bound Complex of **1b**	S28
Solution Phase Anion Binding in NMR	S35
Ion Transport Studies	S40
Supramolecular Channel Formation Proposed Model	S52
NMR Spectra of Compounds	S53
Supplementary References	S68

Supplementary Methods

All reagents used for the synthesis were purchased either from Sigma-Aldrich, Avra, TCI, Spectrochem, and used without further purification. Dry solvents e.g. THF, $CHCl_3$, CH_2Cl_2 and MeOH used for the synthesis were purchased from Merck and used without further drying. All the dry reactions were placed in oven-dried apparatus under nitrogen atmospheric condition. The progress and completion of the reaction was monitored by performing thin layer chromatography experiments where the plates were visualized either by short wave UV light or by different staining reagents (Ninhydrin, PMA, etc.). Column chromatography for purification of the compound was performed using distilled organic solvents on silica gel (100-200 mesh).

All ^{1}H and ^{13}C NMR spectra were recorded using solution of the compound in deuterated solvents, on either Jeol 400 MHz or Bruker 400 MHz NMR spectrometers. The chemical shifts (δ, in ppm unit) were referenced to the residual signals of deuterium solvents (^{1}H NMR $CDCl_3$: δ 7.26 ppm; ^{13}C NMR $CDCl_3$: δ 77.2 ppm; ^{1}H NMR $C_2D_2Cl_4$: δ 6.00 ppm; ^{13}C NMR $C_2D_2Cl_4$: δ 73.8 ppm; ^{1}H NMR DMSO-d_6: δ 2.5 ppm; ^{13}C NMR DMSO-d_6: δ 39.5 ppm). The multiplicities of the peaks are s (singlet), d (doublet), t (triplet), q (quartet), dd (doublet of doublet), m (multiplet). The high-resolution mass spectra (HRMS) were acquired from MicroMass ESI-TOF MS spectrometer and were acquired in the ESI (+ve or −ve) mode. All the fluorescence emission spectra were recorded on a Fluoromax-4 instrument, from Horiba scientific, where experimental cell is equipped with a black injector port and a magnetic stirrer. The experimental data obtained from the fluorescence-based experiments were processed in Origin 8.5 software. Planar lipid bilayer conductance measurements were carried out on a workstation from Warner instrument, USA. The single crystal X-ray diffraction (SCXRD) data collected on a Bruker Smart Apex Duo diffractometer using Mo Kα radiation for all the compounds at either 100 K or 150 K temperature. The field emission scanning electron microscopy (FESEM) images data were obtained using FEI Quanta 3D dual beam ESEM at 3.0 kV. The atomic force microscopy (AFM) images were recorded using Nano Wizard Atomic Force Microscopy. The high-resolution transmission electron microscopy (HRTEM) images were acquired on Jeol USA JEM-2200 FS transmission electron microscope.

Preparation of 7-Nitro-1*H*-indole-2-carboxylic acid 7 ($C_9H_6N_2O_4$): To synthesize the 7-Nitro-1*H*-indole-2-carboxylic acid **7**, we started with 2-nitroaniline **2**, which was converted to (2-nitrophenyl)hydrazine **3** following the reported literature procedure.[1] Subsequently, the (2-nitrophenyl)hydrazine **3** was converted to 7-Nitro-1*H*-indole-2-carboxylic acid **7** in three steps following another literature report.[2] The combined detail synthetic protocol for synthesizing 7-Nitro-1*H*-indole-2-carboxylic acid **7** is provided below.

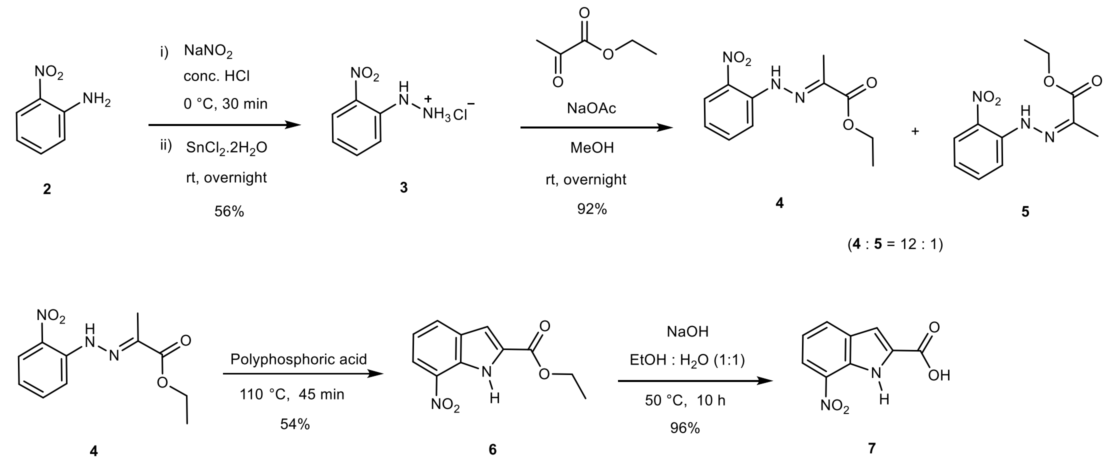

Supplementary Figure 1. Synthesis of indole acid 7. Synthesis of the 7-Nitro-1*H*-indole-2-carboxylic acid **7** in four steps starting from 2-nitroaniline.

In a 500 mL round-bottomed flask 2-nitroaniline (8.0 g, 58 mmol) was taken and dissolved it in 60 mL of conc. HCl, which led to the formation of a suspended solution. The suspended solution was then cooled to −5 ˚C and into that a $NaNO_2$ solution (4.1 g, 60 mmol, in 6 mL water) was added slowly while keeping the reaction mixture temperature below 0 ˚C. After that the reaction solution was stirred at 0 ˚C for about 30 min and then a $SnCl_2·2H_2O$ solution (26.7 g, 119 mmol, in 30 mL conc. HCl) was added slowly. The reaction mixture was then stirred overnight while allowing it to slowly reach to room temperature. After completion of the reaction the precipitate formed was collected via filtration (no additional HCl or water was added during filtration), dried in high vacuum to furnished the orange colour phenylhydrazine hydrochloride product **3** in 56% yield, which was then used for the next step without further purification. Next, in a 100 mL round-bottomed flask the (2-nitrophenyl)hydrazine hydrochloride **3** (5.1 g, 26.9 mmol) and sodium acetate (2.7 g, 32.8 mmol) were taken and dissolved it in 40 mL of dry methanol. After stirring the reaction mixture for 5 min ethyl pyruvate (3.5 g, 30.1 mmol) was added into it slowly and the resultant mixture was then stirred at room temperature for overnight. After completion of the reaction, the reaction mixture was then concentrated and purified via silica gel column

chromatography using ethyl acetate in petroleum ether (1 : 25) as a solvent system to get the pure **4** (*E* isomer) and **5** (*Z* isomer) product separately in 92% overall yield (6.2 g, 24.7 mmol). The subsequent cyclization reaction with polyphosphoric acid should be performed individually using either **4** or **5** as a starting material. **^{1}H NMR (400 MHz, $CDCl_3$):** δ 10.96 (s, 1H), 8.21 (d, *J* = 8.5 Hz, 1H), 8.04 (d, *J* = 8.6 Hz, 1H), 7.62 (t, *J* = 7.8 Hz, 1H), 6.99 (t, *J* = 7.8 Hz, 1H), 4.36 (q, *J* = 7.1 Hz, 2H), 2.24 (s, 3H), 1.40 (t, *J* = 7.1 Hz, 3H).

In a 100 mL round-bottomed flask, 30 mL of polyphosphoric acid (highly viscous liquid) was taken and heated in an oil bath in stirring condition at 110 ˚C to make a less dense liquid. The compound **4** (5.0 g, 19.9 mmol) was then added into it as a solid powder and the combined reaction mixture was then stirred at same temperature (110 ˚C) for 30 min. After completion of the reaction the reaction solution was cool down to 45 ˚C and into that 50 mL of water was added to hydrolyse the excess polyphosphoric acid. The combined reaction mixture was then washed with water (2 × 30 mL) and followed by brine solution (1 × 20 mL) while extracting the compound in ethyl acetate (150 mL). The organic layer was then dried over Na_2SO_4 and the solvent was evaporated in a rotary evaporator to get the crude product. The crude product was purified by silica gel column chromatography using ethyl acetate in petroleum ether (1 : 20) as a solvent system to get the pure indole ester **6** in 54% yield (2.5 g, 11.1 mmol). **^{1}H NMR (400 MHz, $CDCl_3$):** δ 1 0.34 (s, 1H), 8.30 (d, *J* = 8.0 Hz, 1H), 8.05 (d, *J* = 7.9 Hz, 1H), 7.36 (d, *J* = 2.1 Hz, 1H), 7.28 (t, *J* = 7.9 Hz, 1H), 4.46 (q, *J* = 7.1 Hz, 2H), 1.45 (t, *J* = 7.1 Hz, 3H).

To synthesize the indole acid derivative **7**, in a round-bottomed flask, indole ester **6** (2.4 g, 10.3 mmol) and ethanol (10 mL) were taken where the compound formed a suspended solution in that solvent. Then 16 mL of 1.0 M KOH solution was added into that suspension and the reaction mixture was then stirred overnight at 50 ˚C. After completion of the reaction (monitored by TLC) the reaction mixture was poured in 150 mL distilled water, acidified with 2.0 N HCl to regenerate the yellow precipitate, and finally collected precipitate via filtration to get the crude product **7** (2.0 g, 9.7 mmol) in 95% yield. The ^{1}H NMR spectrum of indole acid **7** matched with the reported data. **^{1}H NMR (400 MHz, DMSO-d_6):** δ 13.80 (s, 1H), 11.16 (s, 1H), 8.28 (d, *J* = 7.5 Hz, 1H), 8.23 (d, *J* = 5.1 Hz, 1H), 7.42 (s, 1H), 7.36 (d, *J* = 5.1 Hz, 1H).

Synthesis of bis(indole) compound 1a: The nitro-indole acid compound **7**, synthesized from the above-mentioned procedure was used as a starting material.

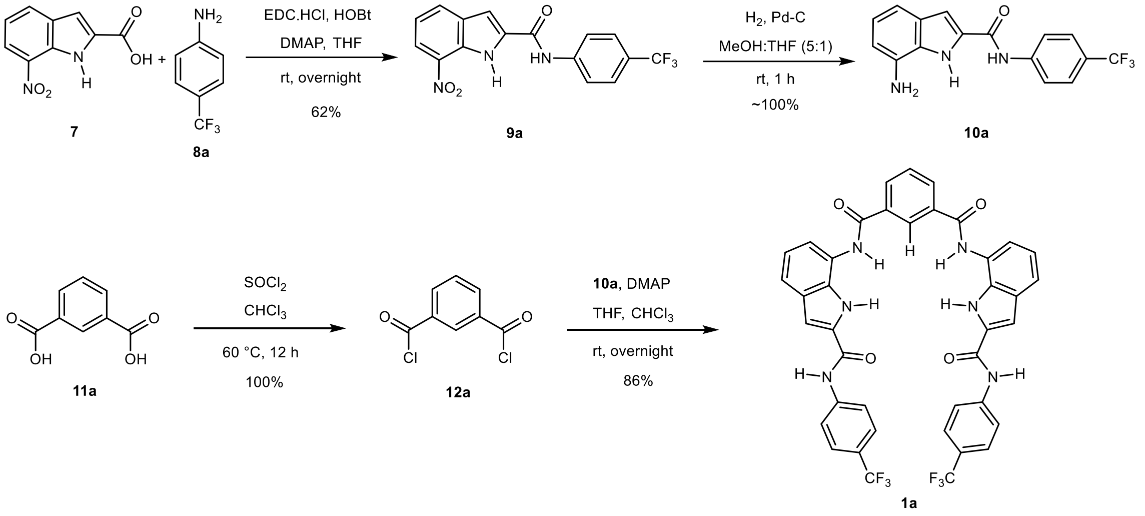

Supplementary Figure 2. Synthesis of bis(indole) compound 1a. The 7-Nitro-1*H*-indole-2-carboxylic acid **7** and Isophthalic acid were used for the synthesis of compound **1a**.

Synthesis of 7-nitro-*N*-(4-(trifluoromethyl)phenyl)-1*H*-indole-2-carboxamide 9a ($C_{16}H_{10}F_3N_3O_3$): In a 50 mL round bottom flask 7-Nitro-1*H*-indole-2-carboxylic acid **7** (0.40 g, 1.94 mmol) and *p*-(trifluoromethyl)aniline **8a** (0.34 g, 2.13 mmol) were taken and dissolved in 8 mL of dry THF. Then into the reaction mixture HOBt (0.29 g, 2.13 mmol), EDC·HCl (0.48 g, 2.52 mmol) and DMAP (0.55 g, 4.46 mmol) were added sequentially. The reaction mixture was then stirred overnight at room temperature under an inert atmosphere. After completion of the reaction, the reaction mixture was washed with water (2 × 30 mL) and followed by brine solution (1 × 20 mL) while extracting the compound in $CHCl_3$ (80 mL). The organic layer was then dried over Na_2SO_4 and the solvent was evaporated in a rotary evaporator to get the crude product. The crude product was purified by silica gel column chromatography using ethyl acetate in petroleum ether (1 : 4) as a solvent system to get the pure amide **9a** in 62% yield (0.42 g, 1.22 mmol). **^{1}H NMR (400 MHz, DMSO-*d*$_6$):** δ 11.59 (s, 1H), 10.96 (s, 1H), 8.28 (dd, *J* = 7.7, 5.7 Hz, 2H), 8.03 (d, *J* = 8.5 Hz, 2H), 7.78 (d, *J* = 8.6 Hz, 2H), 7.66 (s, 1H), 7.37 (t, *J* = 7.9 Hz, 1H); **^{13}C NMR (101 MHz, DMSO-*d*$_6$):** δ 158.5, 142.2, 134.0, 133.2, 130.9, 130.8, 129.1, 126.1 (q, *J* = 3.5 Hz), 124.3 (q, *J* = 271.5 Hz), 123.9 (q, *J* = 31.9 Hz), 121.6, 120.2, 120.1, 108.0; **HRMS (ESI):** Calcd. $C_{16}H_{11}F_3N_3O_3$ $[M+H]^+$: 350.0752, Found: 350.0748.

Synthesis of 7-amino-*N*-(4-(trifluoromethyl)phenyl)-1*H*-indole-2-carboxamide 10a ($C_{16}H_{12}F_3N_3O$): In a 100 mL round bottomed flask the indole-amide **9a** (0.41 g, 1.17 mmol) was dissolved in 25 mL of MeOH:THF (5 : 1) solvent and then the solution was degassed for 30 min using N_2 gas balloon. Then one pinch of Pd-C (10%) was added into that and the solution was stirred under H_2 gas balloon for 1 h at room temperature. After completion of the reaction, the reaction mixture was passed through celite bed while washing with MeOH. The crude product was then purified by silica gel column chromatography using ethyl acetate in petroleum ether (1 : 3) as a solvent system to get the pure amine **10a** in quantitative yield (0.37 g, 1.17 mmol). **^{1}H NMR (400 MHz, DMSO-*d*$_6$):** δ 11.43 (s, 1H), 10.47 (s, 1H), 8.05 (d, J = 8.5 Hz, 2H), 7.74 (d, J = 8.7 Hz, 2H), 7.39 (d, J = 2.1 Hz, 1H), 6.89 (d, J = 7.9 Hz, 1H), 6.84 – 6.76 (m, 1H), 6.41 (d, J = 8.1 Hz, 1H), 5.44 (s, 2H); **^{13}C NMR (101 MHz, DMSO-*d*$_6$):** δ 160.3, 142.8, 134.5, 129.8, 127.8, 126.9, 126.0 (q, J = 3.8 Hz), 124.4 (q, J = 271.3 Hz), 123.3 (q, J = 32.0 Hz), 121.4, 119.8, 109.4, 106.2, 105.0; **HRMS (ESI):** Calcd. $C_{16}H_{13}F_3N_3O$ [M+H]$^+$: 320.1010, Found: 320.1010.

Synthesis of *N*^{1},*N*^{3}-bis(2-((4-(trifluoromethyl)phenyl)carbamoyl)-1*H*-indol-7-yl)isophthalamide 1a ($C_{40}H_{26}F_6N_6O_4$): In a 25 mL round bottomed flask 40 mg of isopthalic acid **11a** (0.24 mmol) was taken and refluxed it for 10 h in the presence of 5 mL of $CHCl_3$:$SOCl_2$ (1:1). After completion of the reaction, volatiles were evaporated and dried in high vacuum for 30 min. Then the acid chloride **12a** was dissolved in 8 mL of dry $CHCl_3$ and added drop wise to a solution of **10a** (0.16 g, 0.49 mmol) and DMAP (0.08 g, 0.60 mmol) in 4 mL of dry THF. The reaction mixture was then stirred at room temperature for overnight under inert atmosphere. After completion of the reaction, the reaction mixture was washed with water (2 × 15 mL) while extracting the compound in $CHCl_3$ (60 mL). The organic layer was then dried over Na_2SO_4 and the solvent was evaporated in a rotary evaporator to get the crude product. The crude product was then purified by silica gel column chromatography using ethyl acetate in petroleum ether (2 : 3) as a solvent system to get the pure amide **1a** in 86% yield (0.16 g, 0.21 mmol). **^{1}H NMR (400 MHz, DMSO-*d*$_6$):** δ 11.92 (s, 2H), 10.61 (s, 2H), 10.40 (s, 2H), 8.66 (s, 1H), 8.27 (dd, J = 7.7, 1.6 Hz, 2H), 8.04 (d, J = 8.6 Hz, 4H), 7.98 (d, J = 7.7 Hz, 2H), 7.79 (t, J = 7.7 Hz, 1H), 7.74 (d, J = 8.8 Hz, 4H), 7.58 (d, J = 2.1 Hz, 2H), 7.56 (d, J = 8.1 Hz, 2H), 7.15 (t, J = 7.8 Hz, 2H); **^{13}C NMR (101 MHz, DMSO-*d*$_6$):** δ 165.6, 160.0, 142.6, 135.3, 131.0, 130.9, 129.3, 128.6, 128.6, 127.6, 126.0 (q, J = 3.7 Hz), 124.4 (q, J = 271.3 Hz), 124.2, 123.5 (q, J = 32.1 Hz), 120.4, 119.9, 118.4, 116.7, 105.2; **^{19}F

NMR (376.6 MHz, DMSO-d_6): δ −60.34; **HRMS (ESI):** Calcd. $C_{40}H_{27}F_6N_6O_4$ $[M+H]^+$: 769.1997, Found: 769.1991.

Synthesis of bis(indole) compound 1b: The nitro-indole acid compound **7**, synthesized from the above-mentioned procedure was used as a starting material.

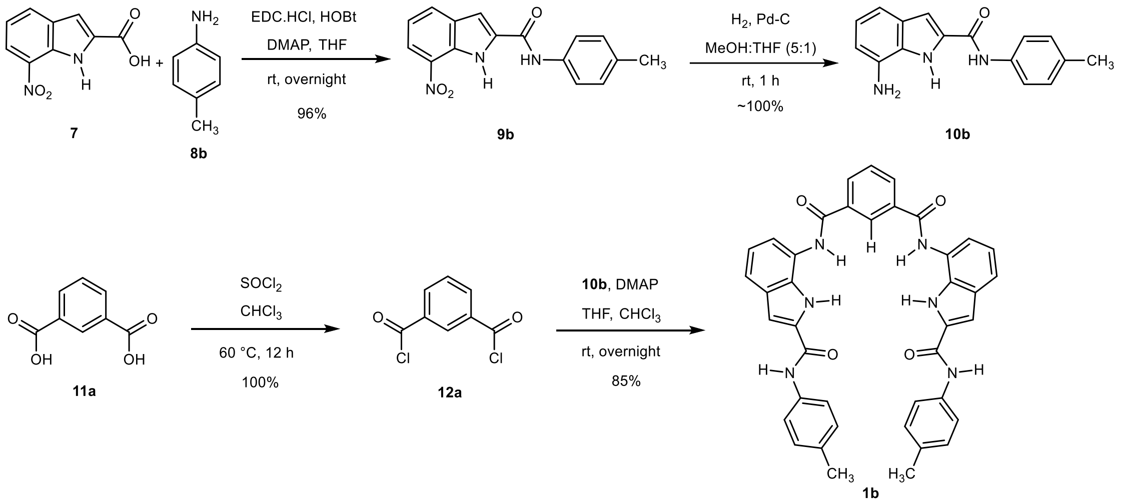

Supplementary Figure 3. Synthesis of bis(indole) compound 1b. The 7-Nitro-1*H*-indole-2-carboxylic acid **7** and Isophthalic acid were used for the synthesis of compound **1b**.

Synthesis of 7-nitro-*N*-(*p*-tolyl)-1*H*-indole-2-carboxamide 9b ($C_{16}H_{13}N_3O_3$): In a 50 mL round bottom flask 7-Nitro-1*H*-indole-2-carboxylic acid **7** (0.60 g, 2.91 mmol) and *p*-toluidine **8b** (0.31 g, 2.91 mmol) were taken and dissolved in 10 mL of dry THF. Then into the reaction mixture HOBt (0.43 g, 3.20 mmol), EDC·HCl (0.73 g, 3.78 mmol) and DMAP (0.90 g, 7.28 mmol) were added sequentially. The reaction mixture was then stirred overnight at room temperature under an inert atmosphere. After completion of the reaction, the reaction mixture was washed with water (2 × 30 mL) and followed by brine solution (1 × 20 mL) while extracting the compound in $CHCl_3$ (100 mL). The organic layer was then dried over Na_2SO_4 and the solvent was evaporated in a rotary evaporator to get the crude product. The crude product was purified by silica gel column chromatography using ethyl acetate in petroleum ether (1 : 4) as a solvent system to get the pure amide **9b** in 96% yield (0.83 g, 2.80 mmol). **^{1}H NMR (400 MHz, DMSO-d_6):** δ 11.51 (s, 1H), 10.60 (s, 1H), 8.26 (t, J = 7.4 Hz, 2H), 7.68 (d, J = 8.3 Hz, 2H), 7.60 (d, J = 1.9 Hz, 1H), 7.37 (d, J = 7.9 Hz, 1H), 7.21 (d, J = 8.3 Hz, 2H), 2.30 (s, 3H); **^{13}C NMR (101 MHz, DMSO-d_6):** δ 157.9, 135.9, 134.6, 133.1,

130.9, 130.7, 129.2, 129.1, 128.9, 121.3, 120.3, 120.0, 107.2, 20.5; **HRMS (ESI):** Calcd. $C_{16}H_{14}N_3O_3$ [M+H]$^+$: 296.1034, Found: 296.1035.

Synthesis of 7-amino-*N*-(*p*-tolyl)-1*H*-indole-2-carboxamide 10b ($C_{16}H_{15}N_3O$): In a 100 mL round bottomed flask the indole-amide **9b** (0.80 g, 2.71 mmol) was dissolved in 40 mL of MeOH:THF (5 : 1) solvent and then the solution was degassed for 30 min using N_2 gas balloon. Then one pinch of Pd-C (10%) was added into that and the solution was stirred under H_2 gas balloon for 1 h at room temperature. After completion of the reaction, the reaction mixture was passed through celite bed while washing with MeOH. The crude product was then purified by silica gel column chromatography using ethyl acetate (1 : 3) in petroleum ether as a solvent system to get the pure amine **10b** in quantitative yield (0.71 g, 2.71 mmol). **^{1}H NMR (400 MHz, DMSO-*d*$_6$):** δ 11.34 (s, 1H), 10.06 (s, 1H), 7.69 (d, J = 8.3 Hz, 2H), 7.31 (d, J = 1.9 Hz, 1H), 7.17 (d, J = 8.3 Hz, 2H), 6.87 (d, J = 7.9 Hz, 1H), 6.80 (t, J = 7.6 Hz, 1H), 6.39 (d, J = 7.2 Hz, 1H), 5.41 (s, 2H), 2.29 (s, 3H).; **^{13}C NMR (101 MHz, DMSO-*d*$_6$):** δ 159.8, 136.5, 134.5, 132.4, 130.5, 129.1, 127.9, 126.6, 121.2, 120.2, 109.3, 106.0, 104.0, 20.5; **HRMS (ESI):** Calcd. $C_{16}H_{16}N_3O$ [M+H]$^+$: 266.1293, Found: 266.1296.

Synthesis of *N*^{1},*N*^{3}-bis(2-(*p*-tolylcarbamoyl)-1*H*-indol-7-yl)isophthalamide 1b ($C_{40}H_{32}N_6O_4$): In a 25 mL round bottomed flask 80 mg of isopthalic acid **11a** (0.48 mmol) was taken and refluxed it for 10 h in the presence of 8 mL of $CHCl_3$:$SOCl_2$ (1:1). After completion of the reaction, volatiles were evaporated and dried in high vacuum for 30 min. Then the acid chloride **12a** was dissolved in 12 mL of dry $CHCl_3$ and added drop wise to a solution of **10b** (0.26 g, 0.72 mmol) and DMAP (0.15 g, 1.20 mmol) in 6 mL of dry THF. The reaction mixture was then stirred at room temperature for overnight under inert atmosphere. After completion of the reaction, the reaction mixture was washed with water (2 × 20 mL) while extracting the compound in $CHCl_3$ (80 mL). The organic layer was then dried over Na_2SO_4 and the solvent was evaporated in a rotary evaporator to get the crude product. The crude product was then purified by silica gel column chromatography using ethyl acetate in petroleum ether (2 : 3) as a solvent system to get the pure amide **1b** in 85% yield (0.27 g, 0.41 mmol). **^{1}H NMR (400 MHz, DMSO-*d*$_6$):** δ 11.82 (s, 2H), 10.40 (s, 2H), 10.22 (s, 2H), 8.67 – 8.62 (m, 1H), 8.26 (dd, J = 7.8, 1.7 Hz, 2H), 7.96 (d, J = 7.6 Hz, 2H), 7.78 (t, J = 7.8 Hz, 1H), 7.68 (d, J = 8.4 Hz, 4H), 7.53 (d, J = 7.9 Hz, 2H), 7.50 (d, J = 2.0 Hz, 2H), 7.17 (d, J = 8.3 Hz, 4H), 7.13 (t, J = 7.9 Hz, 2H), 2.28 (s, 6H); **^{13}C NMR (101 MHz, DMSO-*d*$_6$):** δ 165.6, 159.4, 136.3, 135.3, 132.6, 131.6, 131.0, 129.1, 129.0, 128.7, 128.5, 127.6, 124.2,

120.3, 120.2, 118.2, 116.3, 104.3, 20.5; **HRMS (ESI):** Calcd. $C_{40}H_{33}N_6O_4$ $[M+H]^+$: 661.2563, Found: 661.2559.

Synthesis of bis(indole) compound 1c: The 5-(octyloxy)isophthalic acid **11b** was used as a starting material.

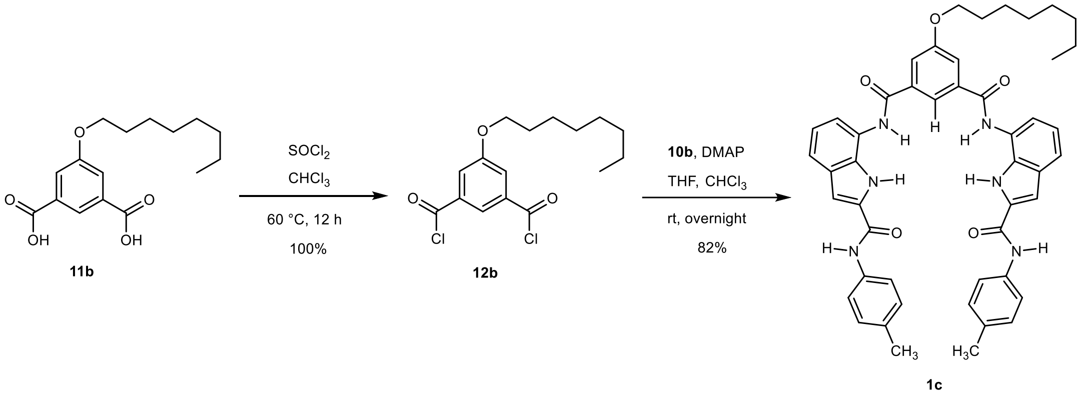

Supplementary Figure 4. Synthesis of bis(indole) compound 1c. The 5-(octyloxy)isophthalic acid **11b** was used for the synthesis of compound **1c**.

Synthesis of 5-(octyloxy)-*N*[1],*N*[3]-bis(2-(*p*-tolylcarbamoyl)-1*H*-indol-7-yl)isophthalamide 1c ($C_{48}H_{48}N_6O_5$): In a 50 mL round bottomed flask 82 mg of 5-(octyloxy)isophthalic acid **11b** (0.28 mmol) was taken and refluxed it for 10 h in the presence of 10 mL of $CHCl_3$:$SOCl_2$ (1:1). After completion of the reaction, volatiles were evaporated and dried in high vacuum for 30 min. Then the acid chloride **12b** was dissolved in 10 mL of dry $CHCl_3$ and added drop wise to a solution of **10b** (0.15 g, 0.56 mmol) and DMAP (0.09 g, 0.74 mmol) in 8 mL of dry THF. The reaction mixture was then stirred at room temperature for overnight under inert atmosphere. After completion of the reaction, the reaction mixture was washed with water (2 × 30 mL) while extracting the compound in $CHCl_3$ (100 mL). The organic layer was then dried over Na_2SO_4 and the solvent was evaporated in a rotary evaporator to get the crude product. The crude product was then purified by silica gel column chromatography using ethyl acetate in petroleum ether as a solvent system to get the pure amide **1c** in 82% yield (0.18 g, 0.23 mmol). **^{1}H NMR (400 MHz, $CDCl_3$):** δ 11.71 (s, 2H), 9.38 (s, 2H), 8.42 (dd, J = 5.9, 2.8 Hz, 2H), 7.71 (s, 2H), 7.42 (s, 1H), 7.03 – 6.88 (m, 6H), 6.78 (d, J = 8.2 Hz, 4H), 6.74 (d, J = 1.7 Hz, 2H), 6.65 (d, J = 8.2 Hz, 4H), 3.99 – 3.78 (m, 2H), 2.16 (s, 6H), 1.99 – 1.81 (m, 2H), 1.53 – 1.32 (m, 12H), 0.99 – 0.91 (m, 3H); **^{13}C NMR (101 MHz, $C_2D_2Cl_4$):** δ

166.3, 160.5, 159.3, 135.6, 134.1, 132.9, 128.6, 128.5, 128.0, 127.9, 123.4, 120.4, 120.1, 118.9, 116.8, 115.5, 115.3, 105.2, 77.3, 67.9, 36.6, 33.7, 29.6, 29.3, 26.2, 24.7, 20.7; **HRMS (ESI):** Calcd. $C_{48}H_{49}N_6O_5$ $[M+H]^+$: 789.3764, Found: 789.3754.

Synthesis of bis(indole) compound 1d: The 5-(octyloxy)isophthalic acid **11b** was used as a starting material.

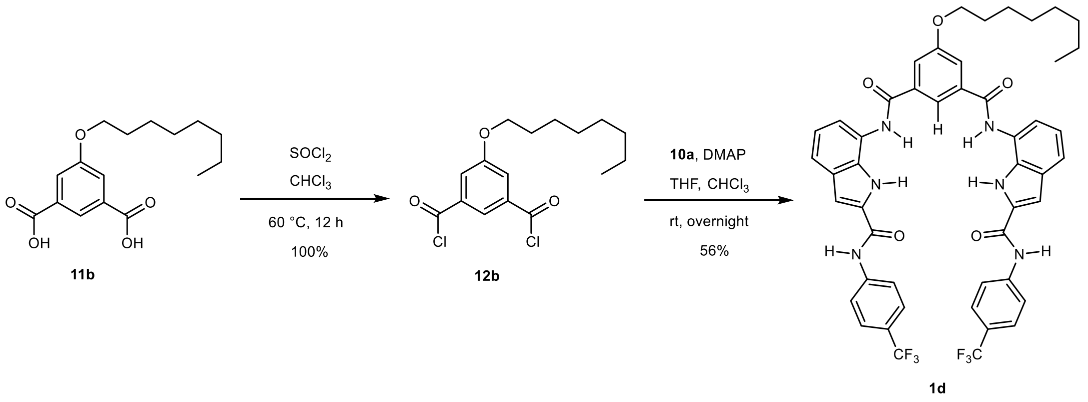

Supplementary Figure 5. Synthesis of bis(indole) compound 1d. The 5-(octyloxy)isophthalic acid **11b** was used for the synthesis of compound **1d**.

Synthesis of 5-(octyloxy)-*N*[1],*N*[3]-bis(2-((4-(trifluoromethyl)phenyl)carbamoyl)-1*H*-indol-7-yl)isophthalamide 1d ($C_{48}H_{42}F_6N_6O_5$): In a 50 mL round bottomed flask 48 mg of 5-(octyloxy)isophthalic acid **11b** (0.16 mmol) was taken and refluxed it for 10 h in the presence of 8 mL of $CHCl_3$:$SOCl_2$ (1:1). After completion of the reaction, volatiles were evaporated and dried in high vacuum for 30 min. Then the acid chloride **12b** was dissolved in 10 mL of dry $CHCl_3$ and added drop wise to a solution of **10a** (0.11 g, 0.33 mmol) and DMAP (0.05 g, 0.41 mmol) in 8 mL of dry THF. The reaction mixture was then stirred at room temperature for overnight under inert atmosphere. After completion of the reaction, the reaction mixture was washed with water (2 × 30 mL) while extracting the compound in $CHCl_3$ (100 mL). The organic layer was then dried over Na_2SO_4 and the solvent was evaporated in a rotary evaporator to get the crude product. The crude product was then purified by silica gel column chromatography using ethyl acetate in petroleum ether as a solvent system to get the pure amide **1d** in 56% yield (0.08 g, 0.09 mmol). **^{1}H NMR (400 MHz, $CDCl_3$):** δ 11.53 (s, 2H), 9.21 (s, 2H), 8.41 (dd, J = 5.1, 3.6 Hz, 2H), 8.08 (s, 2H), 7.31 (s, 1H), 7.14 (d, J = 8.6 Hz, 4H), 7.07 (d, J = 8.5 Hz, 4H), 7.02 – 6.98 (m, 4H), 6.92 (s, 4H), 3.84 (q, J = 6.4 Hz, 2H), 1.92 – 1.82 (m, 2H), 1.66 – 1.39 (m, 10H), 0.98 – 0.93 (m, 3H); **^{13}C NMR (101 MHz,**

$CDCl_3$): δ 166.7, 161.5, 159.9, 138.7, 136.3, 128.7, 128.3, 128.1, 126.6 (q, J = 33.2 Hz), 125.4 (q, J = 3.4 Hz), 125.2, 123.1, 122.5, 121.0, 120.2, 118.5 (q, J = 273.6 Hz), 116.6, 114.9, 107.1, 68.4, 36.9, 32.0, 29.9, 29.4, 26.3, 22.9, 14.3; **^{19}F NMR (376.6 MHz, $CDCl_3$):** δ −62.30; **HRMS (ESI):** Calcd. $C_{48}H_{43}F_6N_6O_5$ $[M+H]^+$: 897.3198, Found: 897.3168.

Synthesis of bis(indole) compound 1e: The nitro-indole ester compound **6**, synthesized from the above-mentioned procedure was used as a starting material.

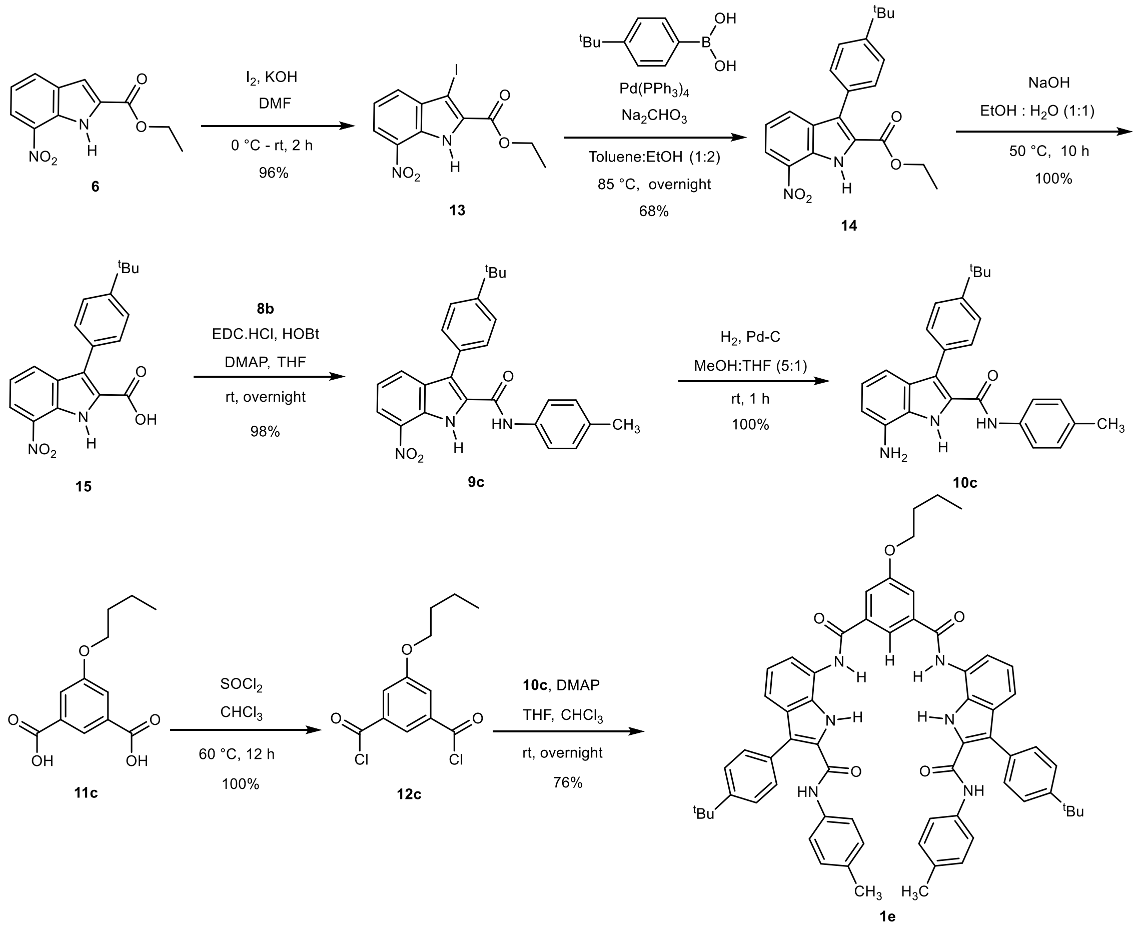

Supplementary Figure 6. Synthesis of bis(indole) compound 1e. The 5-butoxyisophthalic acid **11b** was used for the synthesis of compound **1d**.

In a 25 mL round bottomed flask ethyl 7-nitro-1*H*-indole-2-carboxylate **6** (1.0 g, 4.27 mmol) was taken and dissolved it in 10 mL of dry DMF. The solution was then cooled to 0 ˚C using ice bath. Then into this cooled solution, KOH (1.2 g, 21.35 mmol) was added and the reaction mixture was then stirred at 0 ˚C for 1 h. After that I_2 (1.19 g, 4.70 mmol) was added by dissolving it in 5 mL of dry DMF to the reaction mixture and then the reaction

mixture was stirred for 2 h while allowing it slowly to reach the room temperature. After completion of the reaction, the organic component was extracted in ethyl acetate (100 mL) while washing with water (2 × 30 mL) followed by brine solution (15 mL). The organic layer was then dried over Na_2SO_4 and the solvent was evaporated in a rotary evaporator to get the crude product. The crude product was then purified by silica gel column chromatography using ethyl acetate in petroleum ether as a solvent system to get the pure ester **13** in 96% yield. **^{1}H NMR (400 MHz, $CDCl_3$):** δ 10.54 (s, 1H), 8.37 (dd, *J* = 8.0, 0.9 Hz, 1H), 8.03 – 7.89 (m, 1H), 7.36 (t, *J* = 8.0 Hz, 1H), 4.52 (q, *J* = 7.1 Hz, 2H), 1.50 (t, *J* = 7.1 Hz, 3H); **^{13}C NMR (101 MHz, $CDCl_3$):** δ 159.9, 134.8, 133.5, 132.0, 130.0, 129.6, 123.5, 120.8, 67.2, 62.2; **HRMS (ESI):** Calcd. $C_{11}H_{10}IN_2O_4$ $[M+H]^+$: 360.9685, Found: 360.9688.

For the coupling product **14**, **i**n a 100 mL round bottomed flask iodo ester **13** (1.0 g, 2.78 mmol) and (4-(tert-butyl)phenyl)boronic acid (0.99 g, 5.56 mmol) were taken and dissolved it in 40 mL toluene : ethanol (1:2) solvent system. The solution was then degassed for 30 min using nitrogen gas balloon. The degassed solution was then treated with $Pd(PPh_3)_4$ (0.32 g, 0.28 mmol) and .7 mL of Na_2CO_3 solution (2 M). Then the reaction mixture was refluxed at 85 ˚C for overnight. After completion of the reaction, the solvents were evaporated and the organic component was then extracted in $CHCl_3$ (150 mL) while washing with water (2 × 30 mL) followed by brine solution (15 mL). The organic layer was then dried over Na_2SO_4 and the solvent was evaporated in a rotary evaporator to get the crude product. The crude product was then purified by silica gel column chromatography using ethyl acetate in petroleum ether as a solvent system to get the coupling product **14** in 68% yield. **^{1}H NMR (400 MHz, $CDCl_3$):** δ 10.39 (s, 1H), 8.33 (d, *J* = 7.6 Hz, 1H), 8.03 (d, *J* = 8.0 Hz, 1H), 7.49 (d, *J* = 4.6 Hz, 4H), 7.25 (t, *J* = 8.0 Hz, 1H), 4.36 (q, *J* = 7.2 Hz, 2H), 1.39 (s, 9H), 1.29 (t, *J* = 7.1 Hz, 3H); **^{13}C NMR (101 MHz, $CDCl_3$):** δ 160.9, 151.1, 133.4, 131.9, 130.5, 130.4, 128.9, 128.9, 125.4, 125.2, 125.1, 122.9, 120.1, 61.5, 34.8, 31.5, 14.2.; **HRMS (ESI):** Calcd. $C_{21}H_{23}N_2O_4$ $[M+H]^+$: 367.1657, Found: 367.1656.

In a 50 mL round bottomed flask the coupling product **14** (0.40 g, 1.09 mmol) was taken and dissolved it in 5 mL of ethanol. The solution was then treated with 6 mL of NaOH solution (1.0 M). Then the reaction mixture was stirred under heating condition at 50 ˚C for overnight. After completion of the reaction, the ethanol was evaporated and the remaining mixture was then poured in a 100 mL cold water to get a clear reddish solution. The solution was then treated with dil. HCl until the pH of the solution reaches to 1.0 which leads to the

formation of yellow precipitate. The precipitate was then filtrate out to get pure acid product **15** in quantitative yield. **^{1}H NMR (400 MHz, $CDCl_3$):** δ 10.41 (s, 1H), 8.33 (d, *J* = 7.4 Hz, 1H), 8.02 (d, *J* = 7.7 Hz, 1H), 7.49 (s, 4H), 7.26 – 7.21 (m, 1H), 1.39 (s, 9H); **^{13}C NMR (101 MHz, $CDCl_3$):** δ 165.9, 151.3, 133.4, 131.9, 130.8, 130.4, 129.2, 128.5, 126.6, 125.4, 123.4, 120.3, 114.9, 34.9, 31.5; **HRMS (ESI):** Calcd. $C_{19}H_{19}N_2O_4$ $[M+H]^+$: 339.1345, Found: 339.1341.

For the synthesis of the compound **1e**, at first in a 50 mL round bottom flask 3-(4-(tert-butyl)phenyl)-7-nitro-1*H*-indole-2-carboxylic acid **15** (0.20 g, 0.59 mmol) and *p*-toluidine **8b** (0.07 g, 0.60 mmol) were taken and dissolved in 8 mL of dry THF. Then into the reaction mixture HOBt (0.09 g, 0.65 mmol), EDC.HCl (0.15 g, 0.77 mmol) and DMAP (0.18 g, 1.48 mmol) were added sequentially. The reaction mixture was then stirred overnight at room temperature under an inert atmosphere. After completion of the reaction, the reaction mixture was washed with water (20 mL) and followed by brine solution (15 mL) while extracting the compound in $CHCl_3$ (80 mL). The organic layer was then dried over Na_2SO_4 and the solvent was evaporated in a rotary evaporator to get the crude product. The crude product was purified by silica gel column chromatography using ethyl acetate in petroleum ether as a solvent system to get the pure amide **9c** in 92% yield. **^{1}H NMR (400 MHz, $CDCl_3$):** δ 10.68 (s, 1H), 8.32 (dd, *J* = 8.0, 0.9 Hz, 1H), 7.87 (d, *J* = 7.9 Hz, 1H), 7.67 (d, *J* = 8.5 Hz, 3H), 7.53 (d, *J* = 8.5 Hz, 2H), 7.25 (t, *J* = 8.0 Hz, 1H), 7.08 (s, 4H), 2.29 (s, 3H), 1.45 (s, 9H); **^{13}C NMR (101 MHz, $CDCl_3$):** δ 158.2, 152.9, 134.9, 134.3, 133.5, 132.3, 130.7, 129.7, 129.6, 129.5, 128.7, 128.4, 126.8, 122.2, 120.2, 119.4, 119.3, 35.1, 31.5, 21.0; **HRMS (ESI):** Calcd. $C_{26}H_{26}N_3O_3$ $[M+H]^+$: 428.1974, Found: 428.1980.

In a 100 mL round bottomed flask the indole-amide **9c** (0.15 g, 0.35 mmol) was dissolved in 20 mL of MeOH:THF (5 : 1) solvent and then the solution was degassed for 30 min using N_2 gas balloon. Then one pinch of Pd-C (10%) was added into that and the solution was stirred under H_2 gas balloon for 1 h at room temperature. After completion of the reaction, the reaction mixture was passed through celite bed while washing with MeOH. The crude product was then purified by silica gel column chromatography using ethyl acetate in petroleum ether as a solvent system to get the pure amine **10c** in 98% yield. **^{1}H NMR (400 MHz, $CDCl_3$):** δ 11.10 (s, 1H), 7.87 (s, 1H), 7.63 (d, *J* = 8.4 Hz, 2H), 7.56 (d, *J* = 8.4 Hz, 2H), 7.13 – 7.06 (m, 4H), 6.96 – 6.91 (m, 2H), 6.54 (dd, *J* = 5.5, 2.7 Hz, 1H), 4.17 (bs, 1H), 2.31 (s, 3H), 1.45 (s, 9H); **^{13}C NMR (101 MHz, $CDCl_3$):** δ 161.1, 151.8, 134.8, 134.5, 133.1,

131.0, 130.9, 129.7, 129.4, 126.7, 126.5, 126.3, 121.8, 120.4, 120.2, 110.9, 108.8, 35.0, 31.5, 21.0; **HRMS (ESI):** Calcd. $C_{26}H_{28}N_3O$ $[M+H]^+$: 398.2232, Found: 398.2236.

For the final step, in a 50 mL round bottomed flask 42 mg of isopthalic acid derivative **11c** (0.18 mmol) was taken and refluxed it for 10 h in the presence of 5 mL of $CHCl_3$:$SOCl_2$ (1:1). After completion of the reaction, volatiles were evaporated and dried in high vacuum for 30 min. Then the acid chloride **12c** was dissolved in 8 mL of dry $CHCl_3$ and added drop wise to a solution of **10c** (0.15 g, 0.38 mmol) and DMAP (0.06 g, 0.53 mmol) in 5 mL of dry THF. The reaction mixture was then stirred at room temperature for overnight under inert atmosphere. After completion of the reaction, the reaction mixture was washed with water (2 × 20 mL) while extracting the compound in $CHCl_3$ (50 mL). The organic layer was then dried over Na_2SO_4 and the solvent was evaporated in a rotary evaporator to get the crude product. The crude product was then purified by silica gel column chromatography using ethyl acetate in petroleum ether as a solvent system to get the pure amide **1e** in 76% yield. **^{1}H NMR (400 MHz, $CDCl_3$):** δ 11.95 (s, 2H), 9.65 (s, 2H), 8.83 (d, J = 7.7 Hz, 2H), 7.75 (d, J = 4.4 Hz, 3H), 7.60 (d, J = 7.9 Hz, 4H), 7.54 (d, J = 6.8 Hz, 4H), 7.11 (s, 2H), 6.91 (t, J = 7.9 Hz, 2H), 6.82 (d, J = 7.9 Hz, 2H), 6.52 (d, J = 8.2 Hz, 4H), 6.30 (d, J = 8.2 Hz, 4H), 3.92 (t, J = 6.4 Hz, 2H), 2.13 (s, 6H), 1.87 (dt, J = 14.4, 6.4 Hz, 2H), 1.69 – 1.57 (m, 2H), 1.40 (s, 18H), 1.10 (t, J = 7.4 Hz, 3H); **^{13}C NMR (101 MHz, $CDCl_3$):** δ 165.2, 161.0, 159.1, 151.8, 134.7, 134.3, 133.2, 131.0, 129.9, 129.6, 128.8, 126.9, 126.4, 125.4, 124.3, 121.1, 120.9, 120.5, 117.4, 116.8, 115.8, 115.1, 67.9, 35.0, 31.6, 31.5, 20.9, 19.6, 14.1; **HRMS (ESI):** Calcd. $C_{64}H_{65}N_6O_5$ $[M+H]^+$: 997.5011, Found: 997.5019.

Crystallographic Measurement and Analysis: The single crystal suitable for X-ray analysis for both the complexes **1a** and **1b** were obtained by slow evaporation of ethyl acetate or THF/nitrobenzene solution of the respective compounds. The crystal structure of **1b** with tetrabutylammonium chloride (TBACl) (i.e. **1b′**) was obtained by slow evaporation of solvent from the solution of the receptor with salt in acetonitrile. The single crystal X-ray data collected on a Bruker Smart Apex Duo diffractometer using Mo Kα radiation for both the compounds at 100 K and 150 K respectively. Olex 2 graphical interface[3] were used with ShelXT[4] to solve the structures using intrinsic phasing and refined with ShelXL[5] with full matrix least square minimization on F^2. All non-hydrogen atoms were refined anistropically, except for those in minor disordered parts. Compound **1a** have water molecule as a solvent and two of them have been modelled but neither hydrogen atoms were added nor anisotropic

refinement was not done on them due to disorder. Other two water molecule could not be modelled properly so solvent mask procedure in Olex 2 was adopted which showed electron count of 24 which roughly corresponds to two water molecules and four hydrogen atoms which were not added to the modelled oxygen atoms of the water molecule. Crystallographic parameters for compounds **1a, 1b** and **1b_Cl** are summarised in Supplementary Table 1. The chemical formula of compound **1a** was obtained through a combination of single crystal X-ray, elemental analysis (Supplementary Table 3).

Supplementary Table 1. Crystallographic data for complexes 1a, 1b, and 1b_Cl. Where **1b_Cl** is the crystal structure of **1b** with tetrabutylammonium chloride.

Compound	**1a**	**1b**	**1b_Cl**
Temperature / K	100 K	150 K	100 K
Empirical Formula	$C_{44}H_{38}F_6N_6O_8$	$C_{96}H_{84}N_{14}O_{14}$	$C_{72}H_{104}Cl_2N_8O_4$
Formula Weight [g mol^{-1}]	892.74	1657.77	1216.53
crystal system	Triclinic	Monoclinic	Monoclinic
space group	P-1	$P2_1/c$	$P2_1/c$
a [Å]	15.449(6)	14.4042(18)	8.473(5)
b [Å]	17.681(5)	31.388(4)	19.988(10)
c [Å]	18.086(6)	19.119(3)	39.95(2)
α [°]	104.982(9)	90	90
β [°]	114.745(9)	110.283(3)	92.665(15)
γ [°]	96.945(10)	90	90
cell V [Å^3]	4184(2)	8108.1(19)	6759(6)
Z	4	4	4
reflections collected	38645	175747	96815
independent reflections, R_{int}	16972/0.1571	13927/0.2325	13989
Data/restrains/parameters	16972/0/1129	13927/0/1124	13989/2/773
goodness-of-fit	1.055	1.038	1.024
final *R* indices [I > 2σ(I)]: R_1, wR_2	0.1242, 0.3099	0.0790, 0.1385	0.0968, 0.2223
CCDC no.	2101675	2101678	2101679

$R_1 = \Sigma||F_o| - |F_c||/\Sigma|F_o|$ and $wR_2= |\Sigma w(|F_o|^2 - |F_c|^2)|/\Sigma|w(F_o)^2 |^{1/2}$

Supplementary Table 2. Hydrogen bonding interactions in 1a. Hydrogen bonding interactions present in complex **1a** at 100 K.

D– H···A	D–H(Å)	H···A(Å)	D···A (Å)	<D-H-A(°)	Symmetry#
C6—H6A···O7	0.93	2.59	2.9466(11)	103	0
C26—H26···O6	0.93	2.56	2.9942(12)	109	0
C37—H37···O5	0.93	2.24	2.8404(110	122	0
C55—H55···O2	0.93	2.26	2.7818(11)	115	0
C61—H61···O2	0.93	2.44	2.7503(11)	100	0
C66—H66···O3	0.93	2.34	2.9085(11)	119	0

C75—H75···O4	0.93	2.32	2.8813(11)	119	0
N5—H5···O5	0.86	2.09	2.8388(11)	145	1
N8—H8···O1	0.86	2.29	2.8830(11)	126	1
N9—H9···O1	0.86	2.12	2.9510(11)	161	1
N10—H10···O4	0.86	2.32	3.0345(12)	141	1
N11—H11···O4	0.86	2.12	2.9579(11)	166	1
C23—H23···O1	0.93	2.48	3.2355(13)	139	1
C59—H59···O7	0.93	2.56	3.4197(13)	155	1
C84—H84B···O3	0.96	2.38	3.2233(13)	146	1
N7—H7···O12	0.86	2.14	2.9634(12)	161	2
C67—H67···O3	0.93	2.42	3.3283(13)	164	3
N6—H6···O8	0.86	2.28	3.1085(12)	1625	4
C71—H71···O8	0.93	2.59	3.3289(13)	136	4
N12—H12···O6	0.86	2.14	2.9530(11)	157	5
C35—H35···O6	0.93	2.58	3.2811(13)	132	5
C76—H76···O2	0.93	2.23	3.1537(12)	170	5
#(0) x,y,z; (1) x,y,1+z; (2) 1-x,-y,1-z; (3) 1-x,-y,-z; (4) 1-x,1-y,-z; (5) 1-x,1-y,1-z					

Supplementary Table 3. The elemental analysis of Compound 1a.

Sr. No.	Theoretical %				Observed %			
1	C%	N%	H%	S%	C%	N%	H%	S%
	59.19	9.41	4.29	0.0	58.88	8.41	3.81	0.0

Supplementary Table 4. Hydrogen bonding interactions in 1b. Hydrogen bonding interactions present in complex **1b** at 150 K.

D– H···A	D–H(Å)	H···A(Å)	D···A (Å)	<D-H-A(°)	Symmetry#
N10—H10···N11	0.86	2.61	2.9343(5)	109	0
C15—H15···O2	0.93	2.30	2.8785(5)	120	0
C27—H27···O3	0.93	2.30	2.8292(4)	115	0
C37—H37···O4	0.93	2.42	2.8334(4)	107	0
C46—H46···O5	0.93	2.56	2.9531(5)	106	0
C55—H55···O6	0.93	2.28	2.8560(4)	120	0
C67—H67···O7	0.93	2.40	2.9405(5)	117	0
C75—H75···O8	0.93	2.57	2.9444(5)	105	0
N11—H11···O4	0.86	2.61	2.9343(5)	103	1
C19—H19···O5	0.93	2.52	3.0930(5)	120	1
C19—H19···O8	0.93	2.44	3.2663(5)	148	1
O14—H14B···O13	0.85	1.87	2.7117(4)	173	1
C46—H46···O10	0.93	2.49	3.3588(5)	155	2
C14—H14A···O3	0.85	1.91	2.7309(4)	161	3
C31—H31···O6	0.93	2.57	3.3942(5)	148	4
N12—H12···O7	0.86	2.12	2.9446(5)	162	5
C41—H41A···O9	0.96	2.49	3.3212(5)	146	6
C50—H50···O14	0.93	2.37	3.2108(5)	150	6
#(0) x,y,z; (1) 1/2+x,1/2-y,1/2+z; (2) 1+x,y,z; (3) 1-x,1-y,1-z; (4) 1/2+x,1/2-y,-1/2+z; (5) -1/2+x,1/2-y,-1/2+z; (6) 1/2-x,-1/2+y,1/2-z					

Supplementary Table 5. Hydrogen bonding interactions in 1b_Cl. Hydrogen bonding interactions present in complex **1b_Cl** at 100 K.

D– H···A	D–H(Å)	H···A(Å)	D···A (Å)	<D-H-A(°)	Symmetry#
N2—H2···O2	0.86	2.08	2.6858(16)	127	0
N5—H5···O3	0.86	2.12	2.7195(16)	126	0
C6—H6A···O1	0.93	2.33	2.8857(17)	118	0
C33—H33···O4	0.93	2.34	2.8964(17)	118	0
C89—H89B···Cl1	0.97	2.71	3.477(2)	136	0

C97—H97A···Cl2	0.97	2.6	3.542(2)	164	0
N3—H3···Cl2	0.86	2.35	3.1877(19)	163	1
N4—H4···Cl2	0.86	2.40	3.2213(19)	160	1
C105—H10M···Cl2	0.97	2.69	3.630(2)	164	1
C14—H14···Cl2	0.93	2.8	3.551(2)	138	1
C25—H25···Cl2	0.93	2.73	3.506(2)	142	1
C143—H143···Cl2	0.93	2.75	3.3014(19)	119	1
N1—H1···Cl1	0.86	2.41	3.2328(19)	159	2
C10—H10···Cl1	0.93	2.64	3.459(2)	147	2
C97—H97B···O1	0.97	2.37	3.2755(19)	155	2
N6—H6···Cl1	0.86	2.44	3.2900(19)	168	3
C30—H30···Cl1	0.93	2.59	3.455(2)	156	3
C34—H34···O4	0.93	2.59	3.2038(19)	124	3
#(0) x,y,z; (1) 1+x,y,z; (2) 1-x,-1/2+y,1/2-z; (3) 1-x,1-y,-z					

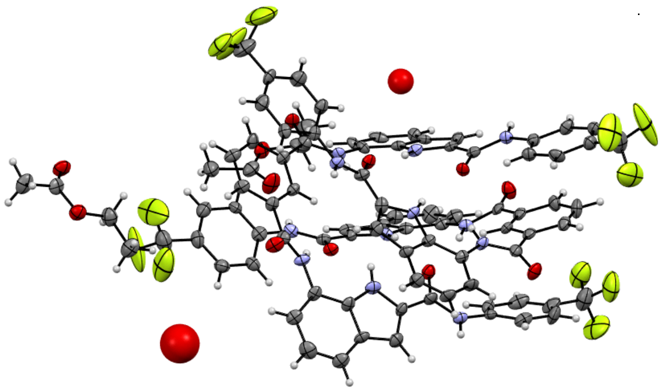

Supplementary Figure 7. The ORTEP diagram of compound 1a. The ellipsoids are shown with 50% probability.

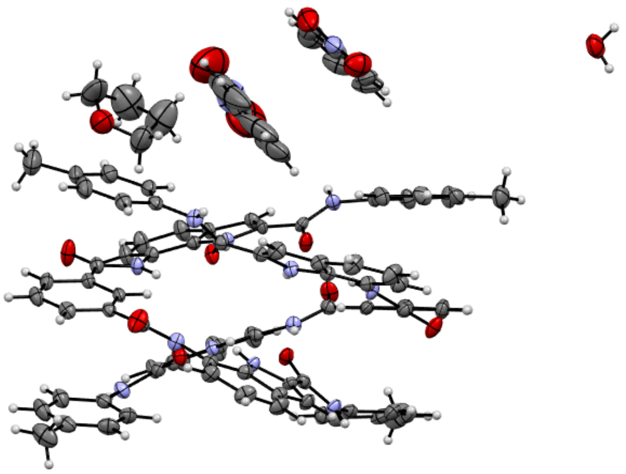

Supplementary Figure 8. The ORTEP diagram of compound 1b. The ellipsoids are shown with 50% probability.

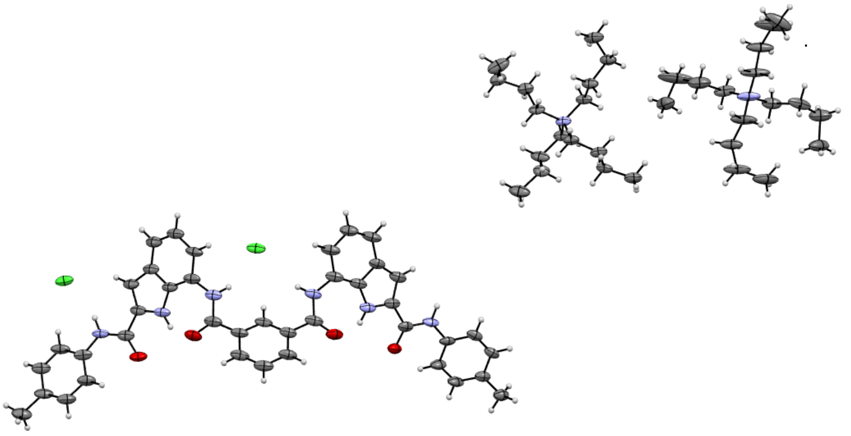

Supplementary Figure 9. The ORTEP diagram of compound 1b_Cl. The ellipsoids are shown with 50% probability.

ESI-MS Spectra of 1a, 1b and Mixture of 1a and 1b: A solution of compound (10 μM) in CH_2Cl_2:CH_3CN (10:1) were prepared and used for electrospray ionization mass spectrometric (ESI-MS) studies in positive mode. The peaks corresponding to $[2M + H]^+$ and/or $[2M + Na]^+$ were observed from ESI-MS experiments (where, M is the exact mass of compound), which evidenced the formation of double helix structure in solution phase.

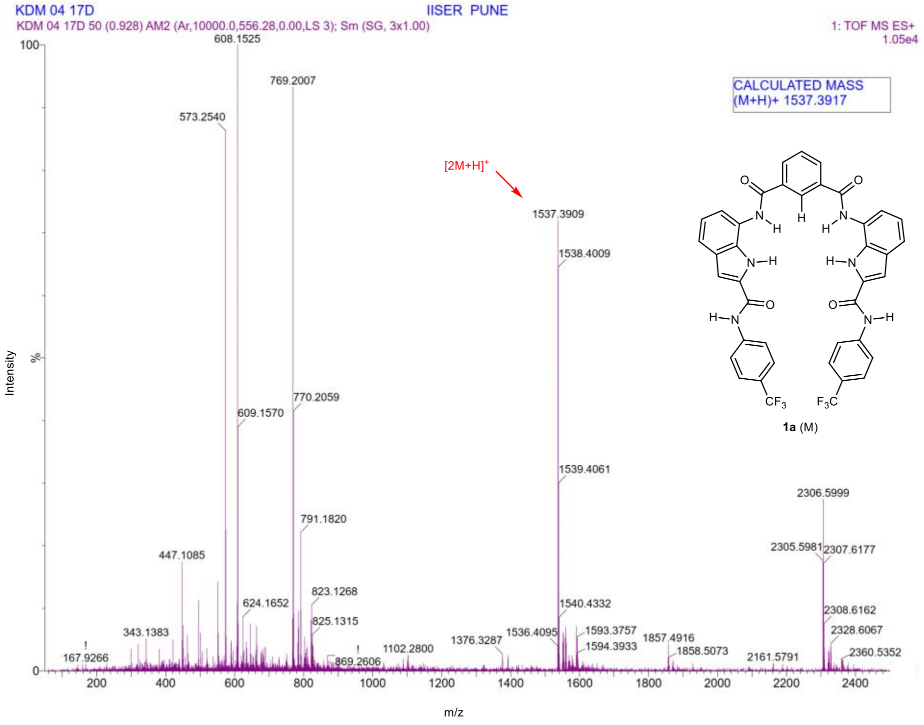

Supplementary Figure 10. The ESI-MS spectrum of 1a in positive mode. The dimeric peak is marked in red color in the spectrum.

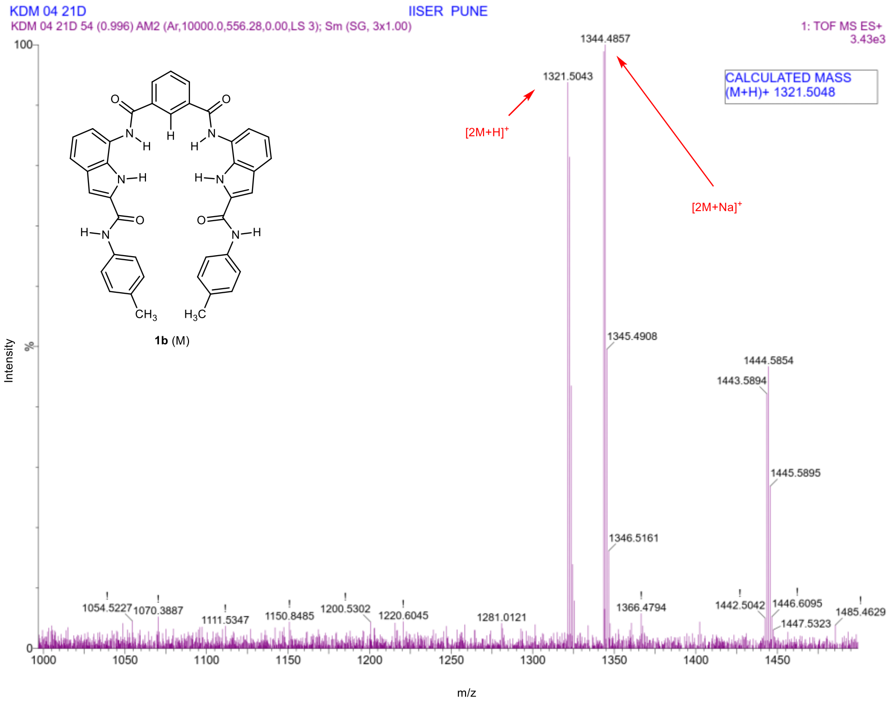

Supplementary Figure 11. The ESI-MS spectrum of 1b in positive mode. The dimeric peak is marked in red color in the spectrum.

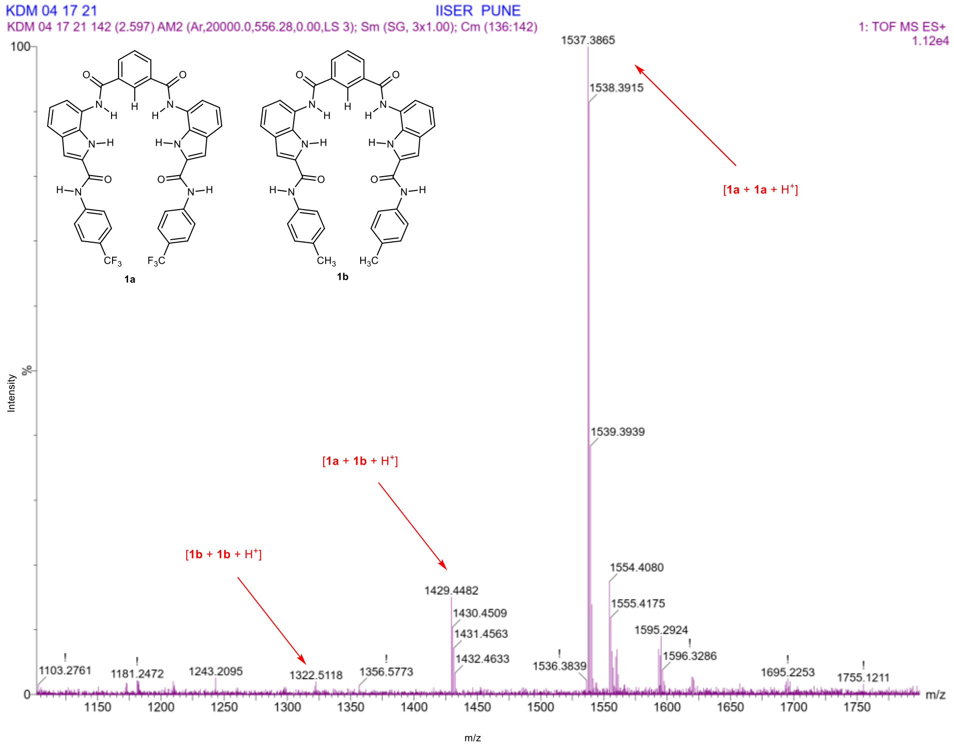

Supplementary Figure 12. The ESI-MS spectrum of 1a and 1b mixture in positive mode. The homo-dimeric ([**1a** + **1a** +H$^+$] and [**1b** + **1b** +H$^+$]) and hetero-dimeric ([**1a** + **1b** +H$^+$]) peaks have been observed and are marked in red colour.

Solution Phase NMR Studies: The formation and the stability of the double helix assembly by the bis(indole) derivative were confirmed by performing various NMR experiments.

2D NMR Studies: All the solution phase studies were performed using **1c** derivative as the **1b** derivative is not soluble in $CDCl_3$. Before performing the solution phase studies, all the acidic protons were assigned by analyzing the 2D NMR data.

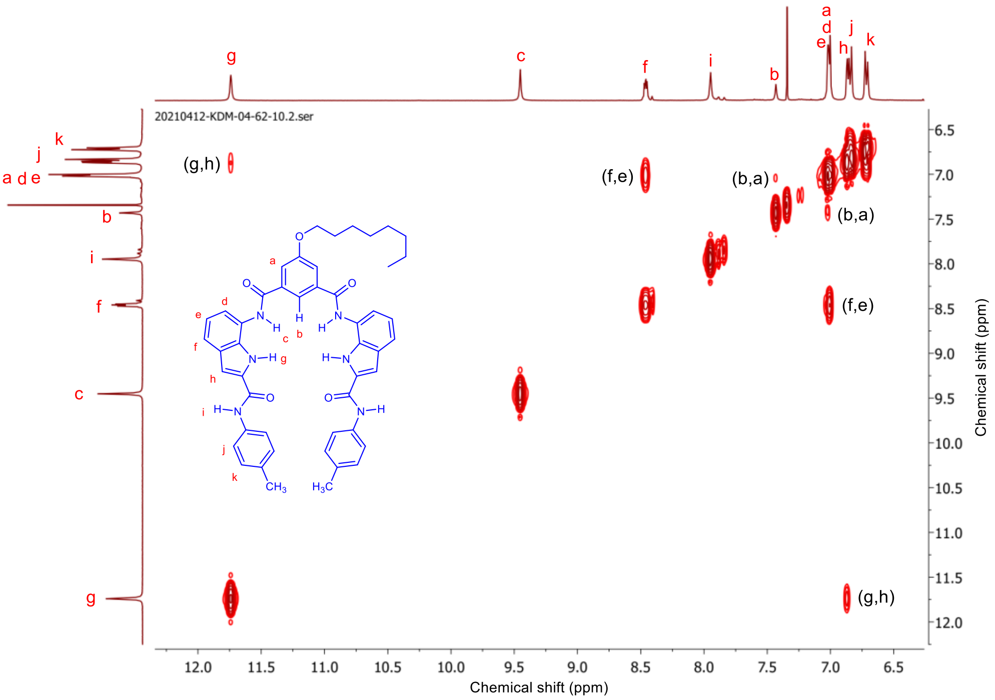

Supplementary Figure 13. The ^{1}H–^{1}H COSY NMR spectrum of 1c. The spectrum was recorded at 25 ˚C (400 MHz, $C_2D_2Cl_4$) with 5.0 mM compound.

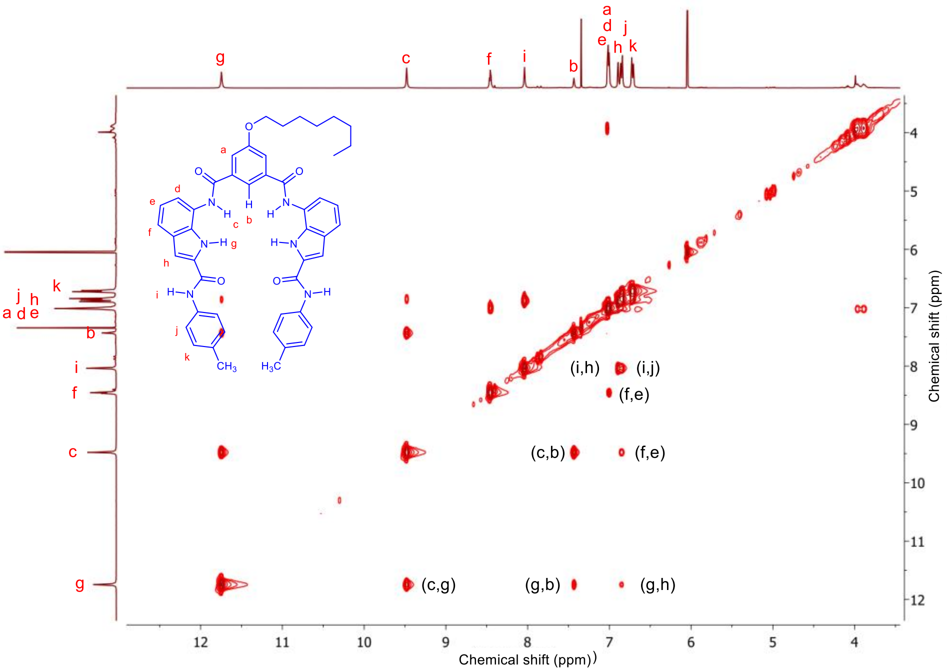

Supplementary Figure 14. The ^1H–^1H NOESY NMR spectrum of 1c. The spectrum was recorded at 25 ˚C (400 MHz, $C_2D_2Cl_4$) with 5.0 mM compound.

Solution Phase NMR of a Mixture of 1c and 1d: The ^1H NMR and ^1H–^1H NOESY NMR experiments were performed by mixing **1c** and **1d** derivatives to see probable self-aggregation.

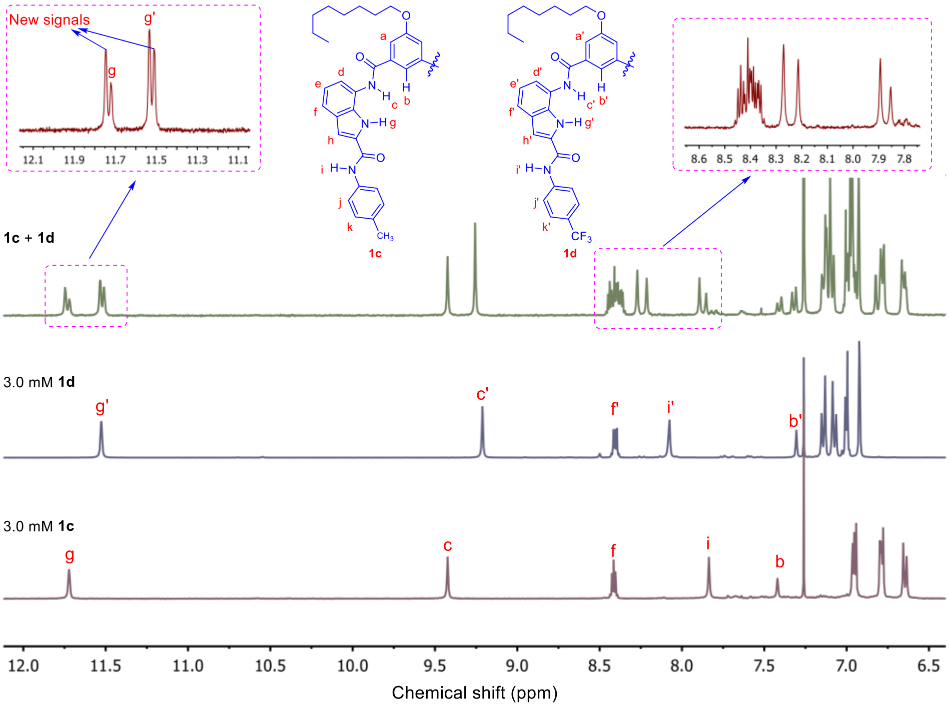

Supplementary Figure 15. The ^1H NMR stacked plot. The stacked plot for ^1H NMR spectra of **1c** (3.0 mM), **1d** (3.0 mM), and a mixture of **1c** and **1d** (3.0 mM + 3.0 mM) in $CDCl_3$ at 25 ˚C (400 MHz).

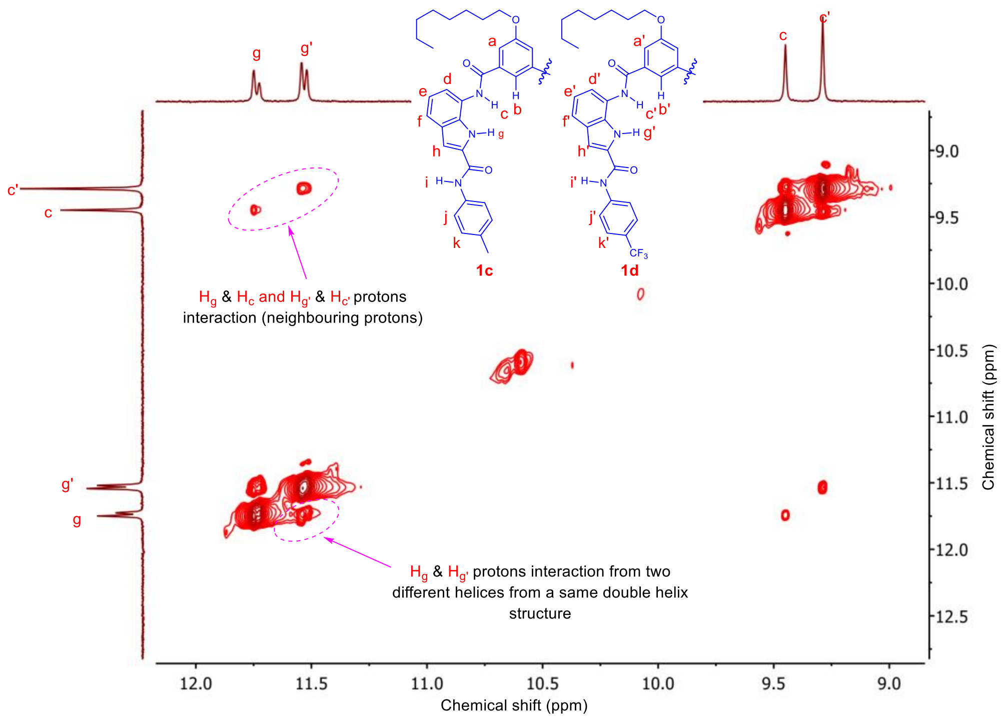

Supplementary Figure 16. The ^{1}H-^{1}H NOESY NMR spectrum of 1c + 1d. The ^{1}H-^{1}H NOESY NMR spectrum of a mixture of **1c** and **1d** (3.0 mM + 3.0 mM) in $CDCl_3$ at 25 ˚C (400 MHz), where the H_g protons from two inter-connected helices are in interactions.

Concentration Dependent NMR Studies of 1c: A series of ^{1}H NMR spectra were recorded for compound **1c** by diluting the sample from 30.0 mM to 0.06 mM in $CDCl_3$.

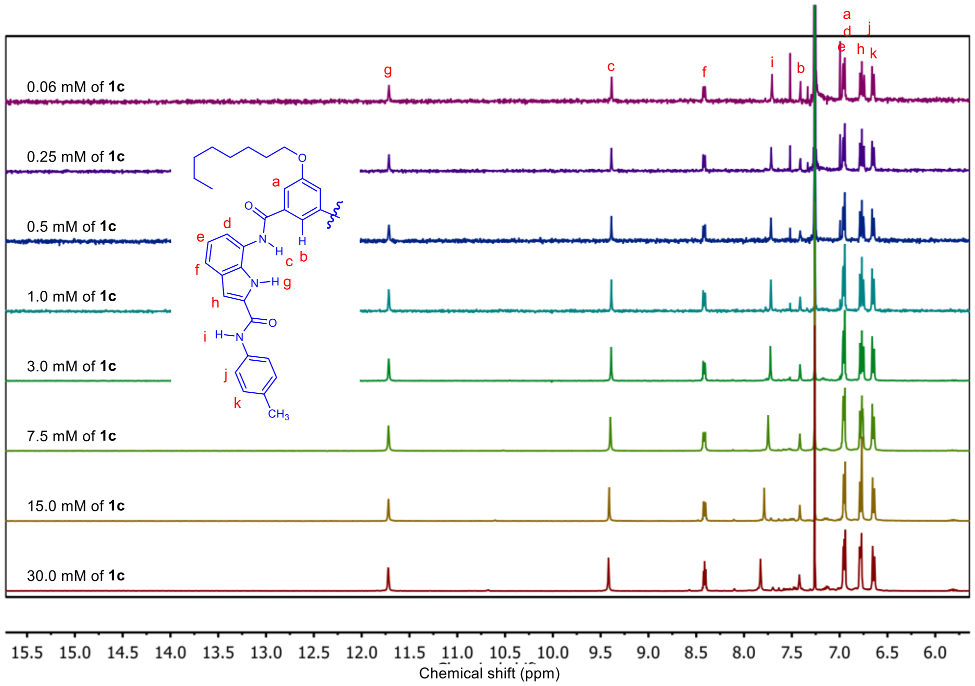

Supplementary Figure 17. The ^{1}H NMR dilution experiment of 1c. The stacked plot for ^{1}H-NMR experiment of **1c** with the successive dilution from 30.0 mM to 0.06 mM at 25 ˚C (400 MHz, $CDCl_3$).

DMSO-d_6 Titration of 1c: The DMSO titration were performed to check the stability of the double-helical assembly in presence of a hydrogen bond acceptor solvent. For that, a 0.5 mL solution was prepared using **1c** (5 mM) in $CDCl_3$ and then recorded the ^1H NMR spectrum. Subsequently, a series of ^1H NMR spectra were recorded with the successive addition of DMSO-d_6 (0–30 mL) at room temperature.

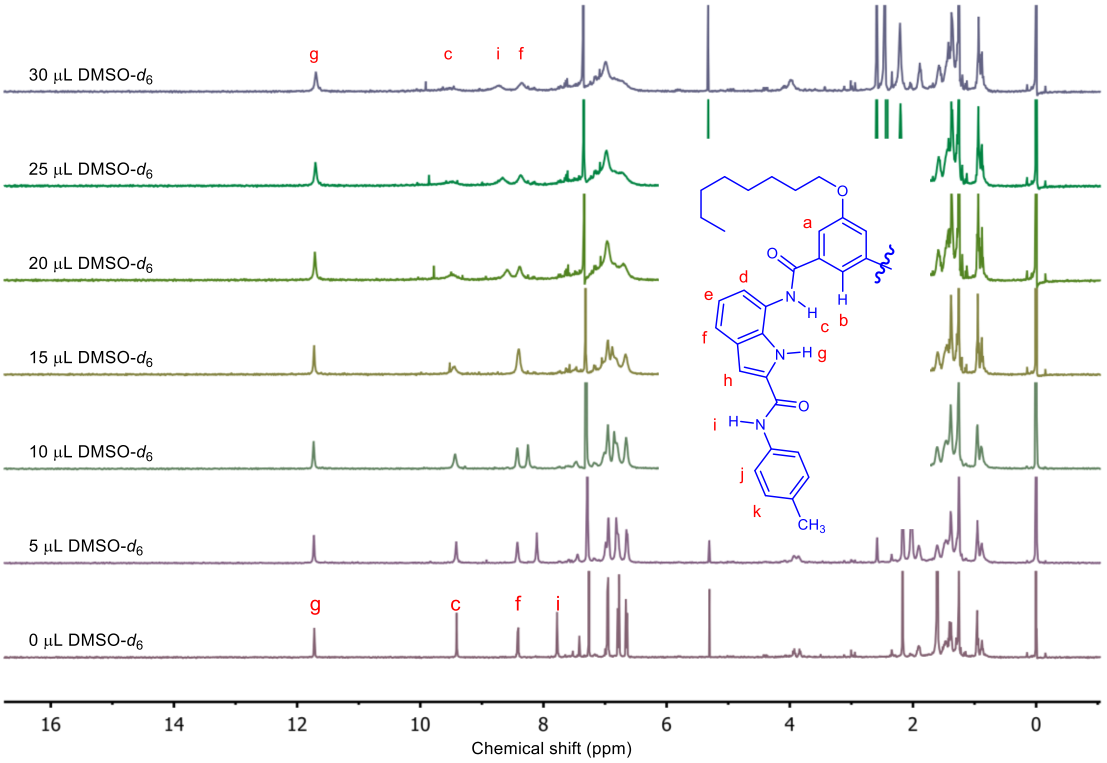

Supplementary Figure 18. The ^1H NMR titration experiment of 1c. The Stacked plot for ^1H-NMR titration experiment for compound **1c** in $CDCl_3$ (0.5 mL) with the successive addition of DMSO-d_6 (0-30 μL) at 25 ˚C (400 MHz). Before stacking all the spectra were calibrated by tetramethylsilane (TMS) peak.

Variable Temperature NMR Experiment of 1c: The variable temperature ^1H NMR experiment were performed to check the stability of the double-helical assembly at higher temperature. For that, a 0.5 mL solution was prepared using **1c** (5 mM) in $C_2D_2Cl_4$ (boiling point 146 ˚C) and then recorded the ^1H NMR spectrum. Subsequently, a series of 1H NMR spectra were recorded while increasing the temperature from 298K to 373K.

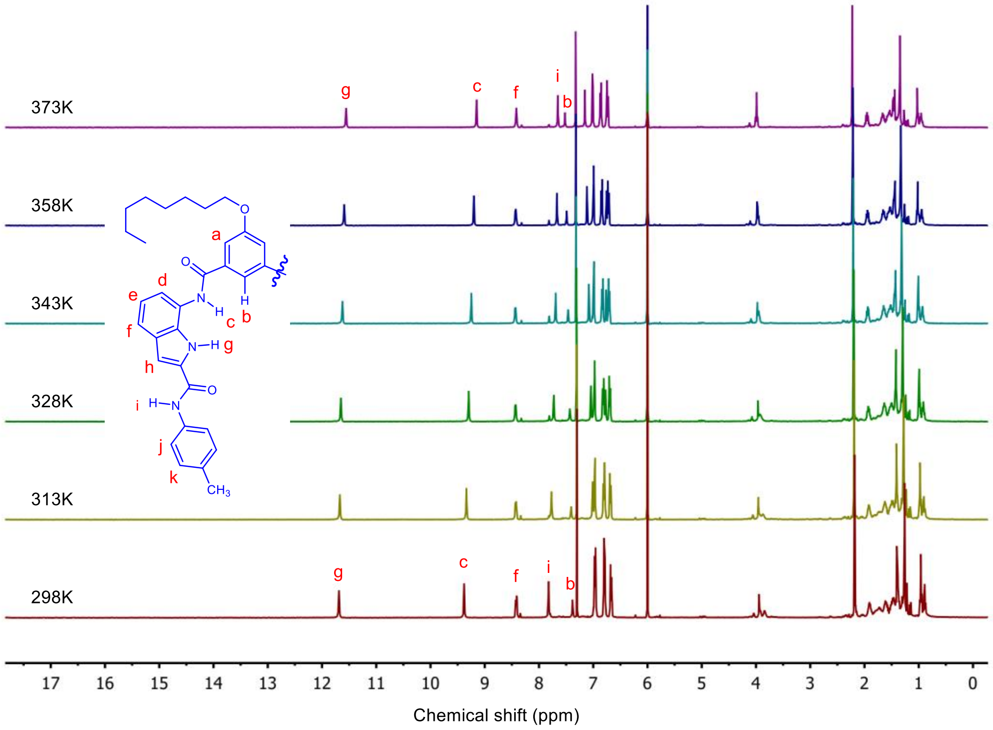

Supplementary Figure 19. The ^{1}H NMR variable temperature experiment of 1c. The stacked plot of variable temperature ^{1}H NMR experiment (298K to 373K) for compound **1c** (5 mM) in $C_2D_2Cl_4$.

The Effect of Urea on 1c in ^{1}H NMR Experiment: It is well known from the literature that the addition of hydrogen bond forming molecules such as urea leads to the decrease in the melting point temperature of the DNA double helix structure.[6, 7] To observe the effect of urea (and cyanuric acid) in the double helix structure formation, ^{1}H NMR experiments were performed for the compound **1c** (5 mM) in the presence and absence of either urea (25 mM) or cyanuric acid (25 mM). The addition of urea or cyanuric acid lead to chemical shift change of only the H_i proton without effecting other protons (Supplementary Figure 20b). This observation is supported by the fact that in the absence of urea the bis(indole) molecules form double helix structure employing the intermolecular hydrogen bonding interactions and H_i proton is not involved in these interactions and participates in the inter-double helical bonding interaction through N–H_i···O=C bonding (Supplementary Figure 20a). With the addition of urea this N-H_i···O=C bonding interaction got disturbed and leads to the downfield shift of the only H_i proton without effecting other acidic protons. These combined observations also support that our bis(indole) molecule form double helix structure in solution, otherwise all acidic proton should have been downfield shifted.

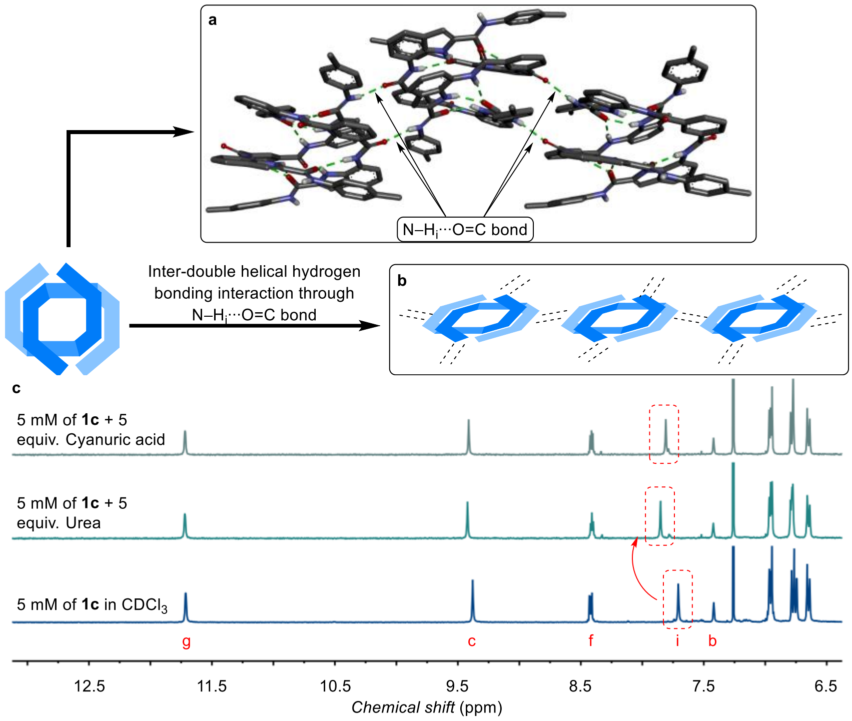

Supplementary Figure 20. The ^{1}H NMR experiment of 1c with urea. The inter-double helical hydrogen bonding interactions in the crystal structure of **1b** (**a**) and in the cartoon representation (**b**) through N-H$_i$···O=C bonding. The ^{1}H NMR spectra of double-helix forming molecule **1c** in absence and presence of urea and cyanuric acid (**c**).

Field Emission Scanning Electron Microscopy (FESEM) Studies for Free Compound 1b: The surface morphology of the compound in solid state was examined by FESEM studies. For that, the compound **1b** was dissolved in acetonitrile-tetrahydrofuran (1:1) solvent system to prepare a 150 μM solution. The compound solution was drop-casted on silicon wafer, dried and then used for FESEM studies.

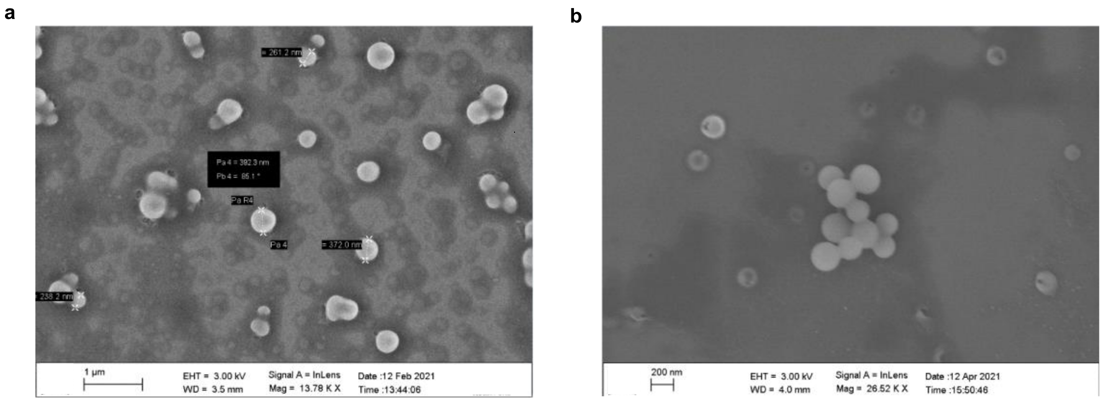

Supplementary Figure 21. The FESEM image of 1b. The FESEM image of free compound **1b**, where the sample preparation was done in acetonitrile-THF solvent (**a**) and chloroform-methanol solvent system (**b**).

FESEM Studies for Compound with 2.0 and 6.0 Equivalent of TBACl: For that, the compound **1b** was dissolved in acetonitrile-tetrahydrofuran (1:1) solvent system to prepare a 150 µM solution and into that solution 2.0 and 6.0 equivalents of TBACl was added separately in two solutions. The compound solutions were drop-casted on silicon wafer, dried and then used for FESEM studies.

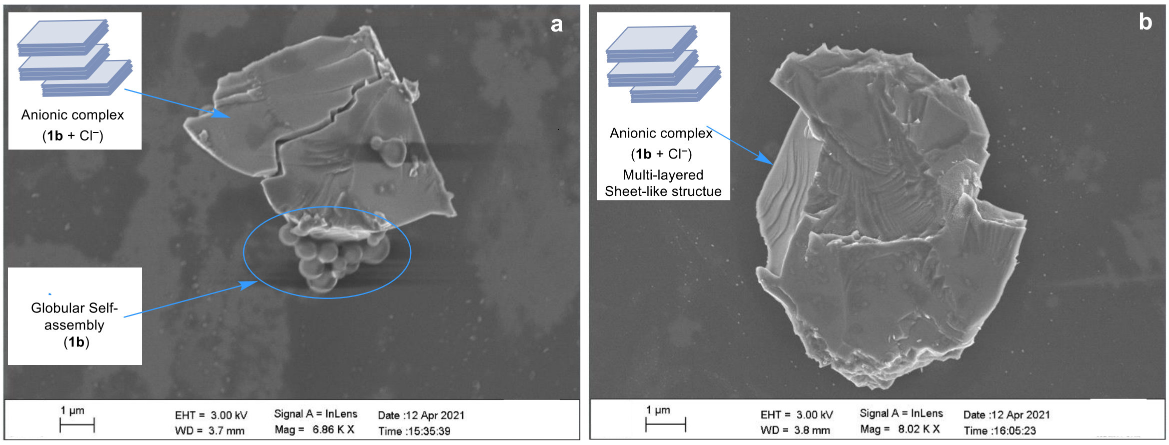

Supplementary Figure 22. The FESEM image of 1b with TBACl. The FESEM images of compound **1b** with 2.0 (**a**) and 6.0 (**b**) equivalents of TBACl.

Atomic Force Microscopy (AFM) Studies for Free Compound 1b: A 100 µM solution the compound **1b** was prepared in acetonitrile-THF (1:1) solvent system. The compound solution was then drop-casted on silicon wafer, dried and then used for AFM studies.

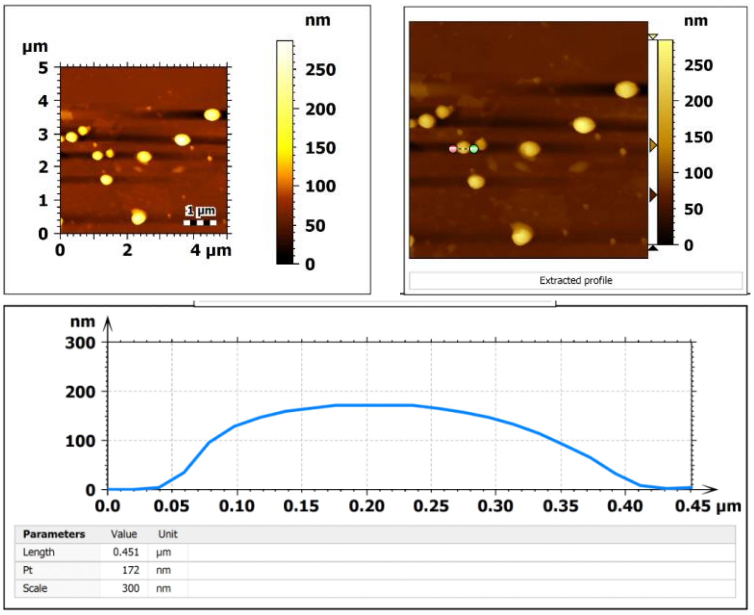

Supplementary Figure 23. The AFM image of 1b. The AFM image of free compound **1b,** where the sample preparation was done in acetonitrile-THF solvent.

AFM Studies for Compound with 6.0 Equivalent of TBACl: A 100 μM solution the compound **1b** was prepared in acetonitrile-tetrahydrofuran (1:1) solvent system and into that solution 6.0 equivalents of TBACl was added. The compound solutions were then drop-casted on silicon wafer, dried and then used for AFM studies.

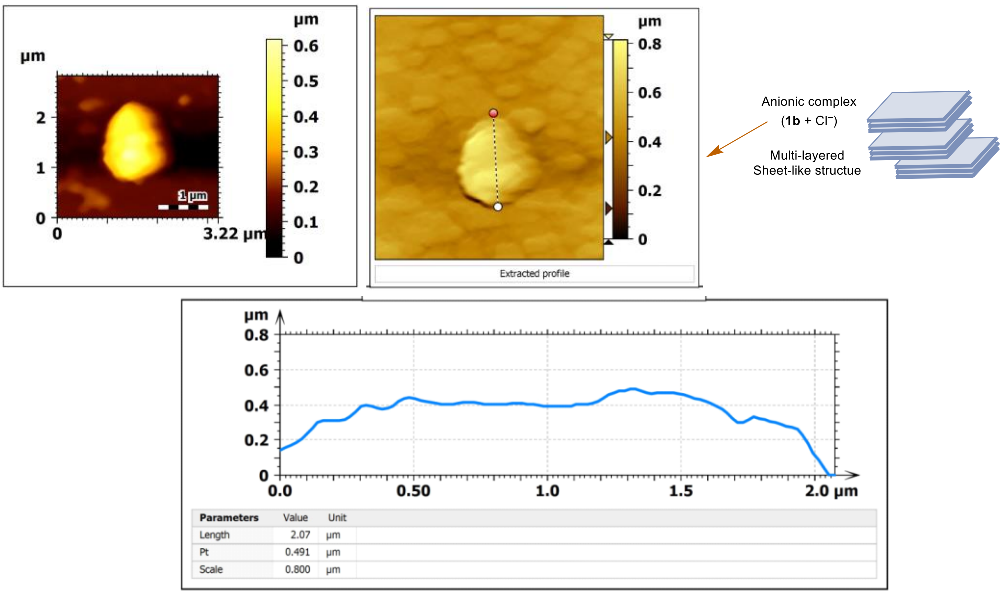

Supplementary Figure 24. The AFM image of 1b with TBACl. The AFM image of compound **1b** with 6.0 equivalents of TBACl, where the sample preparation was done in acetonitrile-THF solvent.

High-Resolution Transmission Electron Microscopy (HRTEM) Studies for Free Compound 1b: A 80 μM solution of the compound **1b** was prepared in in acetonitrile-tetrahydrofuran (1:1) solvent system. The compound solution was then drop-casted on TEM grid, dried and then used for HRTEM imaging.

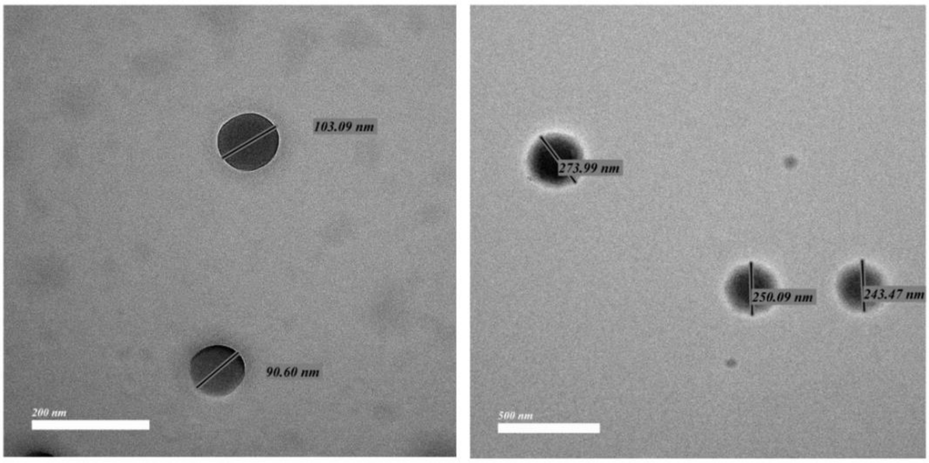

Supplementary Figure 25. The HRTEM image of 1b. The HRTEM image of free compound **1b,** where the sample preparation was done in acetonitrile-THF solvent.

HRTEM Studies for Compound with 6.0 Equivalent of TBACl: A 80 μM solution the compound **1b** was prepared in acetonitrile-tetrahydrofuran (1:1) solvent system and into that solution 6.0 equivalents of TBACl was added. The compound solutions were then drop-casted on TEM grid, dried and then used for HRTEM studies.

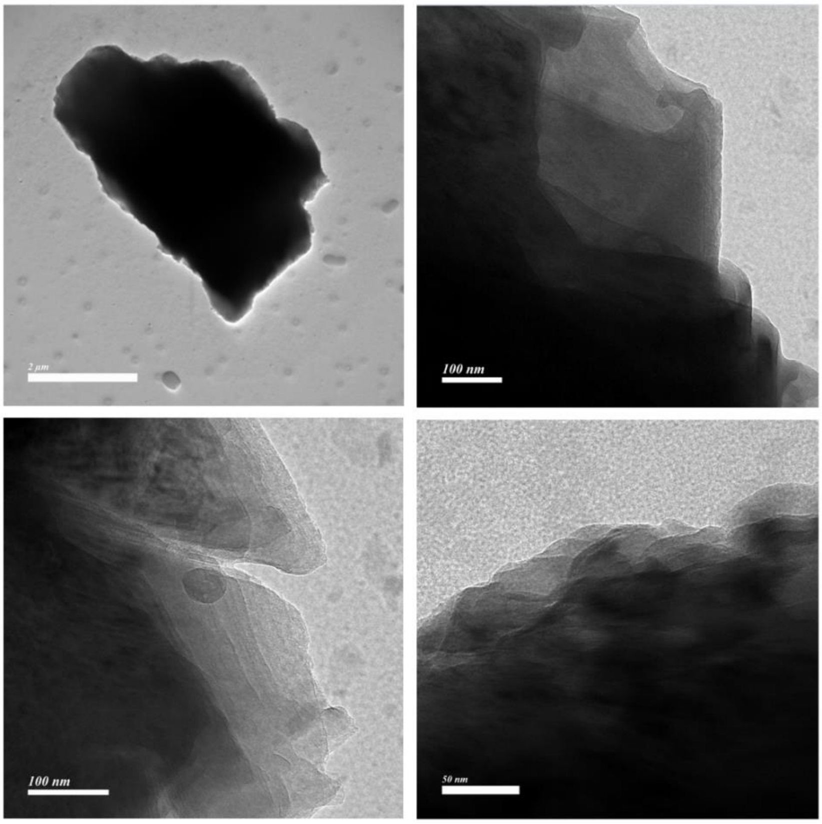

Supplementary Figure 26. The HRTEM image of 1b with TBACl. The HRTEM image of compound **1b** with 6.0 equivalents of TBACl**,** where the sample preparation was done in acetonitrile-THF solvent.

ESI-MS Spectrum of Cl^{-} bound Complex of 1b: The formation of the Cl^{-} ion bound complex by unwinding of the double helical structure of bis(indole) molecule **1b** in solution phase was confirmed from ESI-MS studies. For that, a solution of **1b** (10 μM) was prepared in CH_2Cl_2:CH_3OH (10:1), and into that solution 3.0 equivalents of tetramethylammonium chloride (TMACl) was added. Then the ESI-MS spectrum was recorded in positive (+ve) mode. While analyzing the mass spectrum data, the calculated masses (Supplementary Table 6) are observed as major signals in the recorded spectrum (Supplementary Figure 27), where the computed pattern is found to follow a nice repeating trend. In the repeating trend, the compounds are bound with Cl^{-} and the TMA^{+} ion to make it singly (+1) or doubly (+2) positively charged species. The peak corresponding to different aggregated polymeric units were observed from ESI-MS experiment and are listed there in below table (Supplementary

Table 6; where, M is the exact mass of **1b**), which evidenced the formation of supramolecular polymeric complex in the solution phase.

We also recorded the ESI-MS data for the same combination of compound (i.e. **1b** with TMACl salt) in negative (–ve) mode. The calculated masses (Supplementary Table 7) are observed as major signals in the recorded spectrum (Supplementary Figure 28), where the computed pattern is found to follow a nice repeating trend. In the repeating trend, the compounds are bound with Cl^{-} and the TMA^{+} ion to make it singly (–1) or doubly (–2) negatively charged species. The peak corresponding to different aggregated polymeric units were observed from ESI-MS experiment and are listed there in below table (Supplementary Table 7; where, M is the exact mass of **1b**)

As we have recorded the spectrum with the m/z value of up to 2000, the singly charged species of higher complex has not been observed.

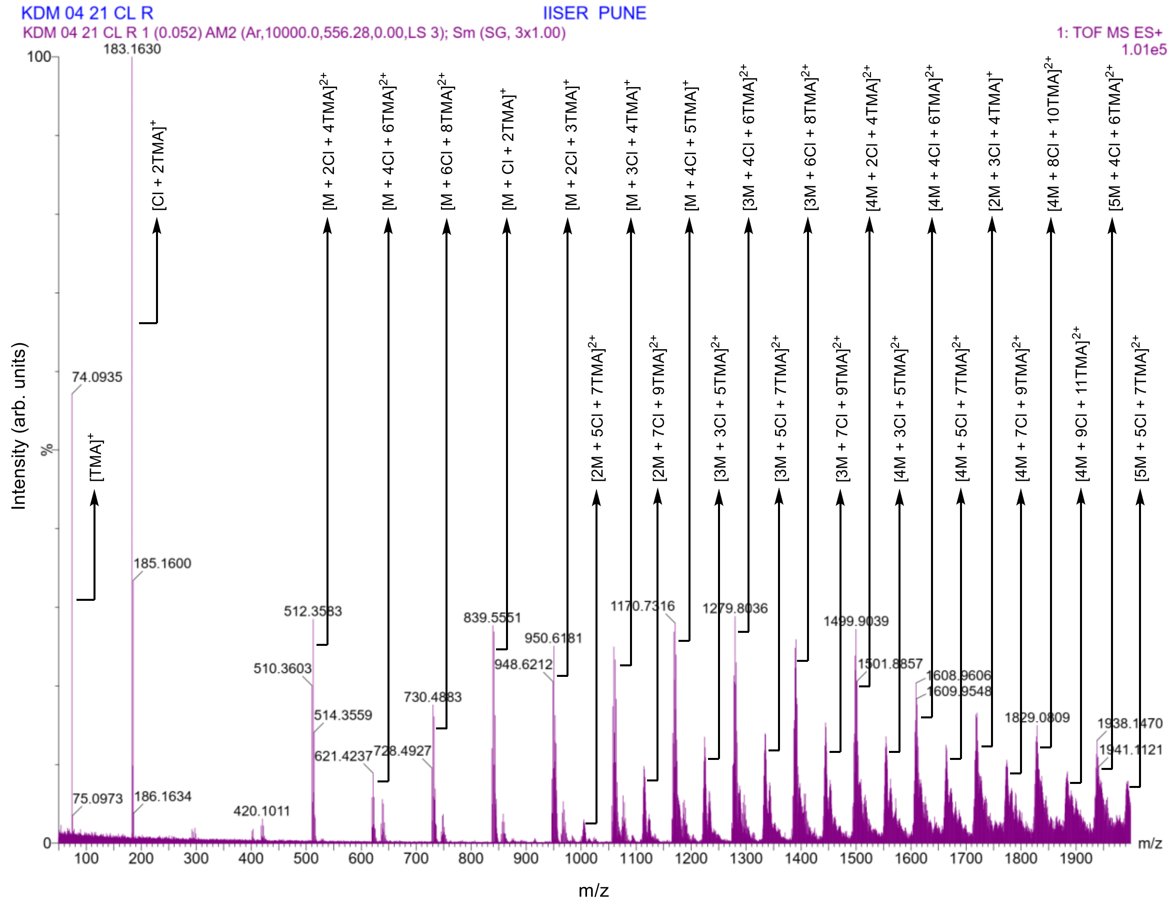

Supplementary Figure 27. The ESI-MS experiment of 1b with TBACl. The ESI-MS spectrum of **1b** with Cl^{-} in positive (+ve) mode.

Supplementary Table 6. Calculated and observed ESI-MS mass of **1b** with Cl^{-} (where M = **1b**) in positive (+ve) mode.

Sr. No.	General Formulae (For M, 2M, 3M, 4M and 5M)	m/z (+ve mode)	Calculated & Matched Signals
1	$[M + n\,Cl + (n+1)TMA]^{+}$ (where n = *1*, *2*, *3*, *4* or *5*)	$[M + Cl + 2TMA]^{+}$	843.41
		$[M + 2Cl + 3TMA]^{+}$	952.47
		$[M + 3Cl + 4TMA]^{+}$	1061.54
		$[M + 4Cl + 5TMA]^{+}$	1170.61
		$[M + 5Cl + 6TMA]^{+}$	1279.67
2	$[M + n\,Cl + (n+2)TMA]^{2+}$ (where n = *2*, *3*, *4*, *5* or *6*)	$[M + 2Cl + 4TMA]^{2+}$	513.28
		$[M + 3Cl + 5TMA]^{2+}$	567.81 (Low)
		$[M + 4Cl + 6TMA]^{2+}$	622.35
		$[M + 5Cl + 7TMA]^{2+}$	676.88
		$[M + 6Cl + 8TMA]^{2+}$	731.41
3	$[2M + n\,Cl + (n+1)TMA]^{+}$ (where n = *1*, *2*, *3*, *4* or *5*)	$[2M + Cl + 2TMA]^{+}$	1503.66
		$[2M + 2Cl + 3TMA]^{+}$	1612.73
		$[2M + 3Cl + 4TMA]^{+}$	1721.79
		$[2M + 4Cl + 5TMA]^{+}$	1830.86
		$[2M + 5Cl + 6TMA]^{+}$	1939.92
4	$[2M + n\,Cl + (n+2)TMA]^{2+}$ (where n = *2*, *3*, *4*, *5*, *6* or *7*)	$[2M + 2Cl + 4TMA]^{2+}$	843.41
		$[2M + 3Cl + 5TMA]^{2+}$	897.94
		$[2M + 4Cl + 6TMA]^{2+}$	952.47
		$[2M + 5Cl + 7TMA]^{2+}$	1007.01
		$[2M + 6Cl + 8TMA]^{2+}$	1061.54
		$[2M + 7Cl + 9TMA]^{2+}$	1116.08

5	[3M + *n* Cl + (*n+1*)TMA]$^{+}$ (where *n* = *2, 3*,or higher)	Above 2000	Above 2000
6	[3M + *n* Cl + (*n+2*)TMA]$^{2+}$ (where *n* = *2, 3, 4, 5, 6, 7* or *8*)	[3M + 2Cl + 4TMA]$^{2+}$	1173.53
		[3M + 3Cl + 5TMA]$^{2+}$	1228.07
		[3M + 4Cl + 6TMA]$^{2+}$	1282.60
		[3M + 5Cl + 7TMA]$^{2+}$	1337.13
		[3M + 6Cl + 8TMA]$^{2+}$	1391.67
		[3M + 7Cl + 9TMA]$^{2+}$	1446.20
		[3M + 8Cl + 10TMA]$^{2+}$	1500.73
7	[4M + *n* Cl + (*n+1*)TMA]$^{+}$ (where *n* = *3, 4*,or higher)	Above 2000	Above 2000
8	[4M + *n* Cl + (*n+2*)TMA]$^{2+}$ (where *n* = *3, 4, 5, 6, 7, 8, 9* or *10*)	[4M + 3Cl + 5TMA]$^{2+}$	1558.19
		[4M + 4Cl + 6TMA]$^{2+}$	1612.73
		[4M + 5Cl + 7TMA]$^{2+}$	1667.26
		[4M + 6Cl + 8TMA]$^{2+}$	1721.79
		[4M + 7Cl + 9TMA]$^{2+}$	1776.32
		[4M + 8Cl + 10TMA]$^{2+}$	1830.86
		[4M + 9Cl + 11TMA]$^{2+}$	1885.39
		[4M + 10Cl + 12TMA]$^{2+}$	1939.92
9	[5M + *n* Cl + (*n+1*)TMA]$^{+}$ (where *n* = *4, 5*,or higher)	Above 2000	Above 2000
10	[5M + *n* Cl + (*n+2*)TMA]$^{2+}$ (where *n* = *4, 5* or *6*,)	[5M + 4Cl + 6TMA]$^{2+}$	1942.85
		[5M + 5Cl + 7TMA]$^{2+}$	1997.38
		[5M + 6Cl + 8TMA]$^{2+}$	Above 2000

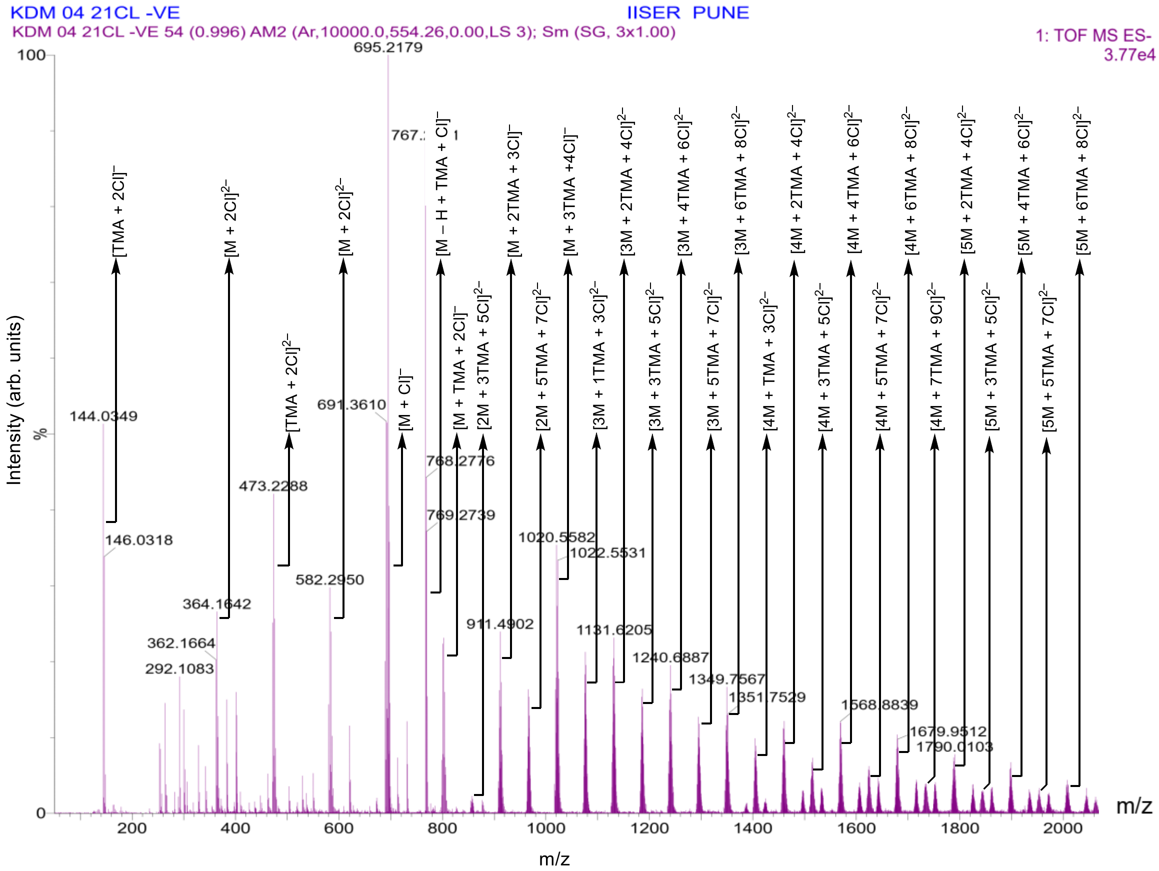

Supplementary Figure 28. The ESI-MS experiment of 1b with TBACl. The ESI-MS spectrum of **1b** with Cl^- in negative (–ve) mode.

Supplementary Table 7. Calculated and observed ESI-MS mass of **1b** with Cl^- (where M = **1b**) in negative (–ve) mode.

Sr. No.	General Formulae (For M, 2M, 3M, 4M and 5M)	m/z (–ve mode)	Calculated & Matched Signals
1	$[M + n\,TMA + (n+1)Cl]^-$ (where $n = 1, 2, 3$ or 4)	$[M + TMA + 2Cl]^-$	804.28
		$[M + 2TMA + 3Cl]^-$	913.35
		$[M + 3TMA + 4Cl]^-$	1022.42
		$[M + 4TMA + 5Cl]^-$	1131.48
2	$[M + n\,TMA + (n+2)Cl]^{2-}$ (where $n = 0, 1$ or 2)	$[M + 2Cl]^{2-}$	365.09
		$[M + TMA + 3Cl]^{2-}$	419.62
		$[M + 2TMA + 4Cl]^{2-}$	474.15

3	[2M + *n* TMA + (*n*+*1*)Cl]$^{-}$ (where *n* = *1*, *2*, *3*, *4* or *5*)	[2M + TMA + 2Cl]$^{-}$	1464.53
		[2M + 2TMA + 3Cl]$^{-}$	1573.60
		[2M + 3TMA + 4Cl]$^{-}$	1682.66
		[2M + 4TMA + 5Cl]$^{-}$	1791.73
		[2M + 5TMA + 6Cl]$^{-}$	1900.80
4	[2M + *n* TMA + (*n*+*2*)Cl]$^{2-}$ (where *n* = *1*, *2*, *3*, *4*, *5* or *6*)	[2M + TMA + 3Cl]$^{2-}$	749.75
		[2M + 2TMA + 4Cl]$^{2-}$	804.28
		[2M + 3TMA + 5Cl]$^{2-}$	858.81
		[2M + 4TMA + 6Cl]$^{2-}$	913.35
		[2M + 5TMA + 7Cl]$^{2-}$	967.88
		[2M + 6TMA + 8Cl]$^{2-}$	1022.42
5	[3M + *n* TMA + (*n*+*1*)Cl]$^{-}$ (where *n* = *1*, *2*, *3*, or *4*)	Above 2000	Above 2000
6	[3M + *n* TMA + (*n*+*2*)Cl]$^{2-}$ (where *n* = 0, *1*, *2*, *3*, *4*, *5*, *6* or *7*)	[3M + 2Cl]$^{2-}$	1025.34
		[3M + TMA + 3Cl]$^{2-}$	1079.88
		[3M + 2TMA + 4Cl]$^{2-}$	1134.41
		[3M + 3TMA + 5Cl]$^{2-}$	1188.94
		[3M + 4TMA + 6Cl]$^{2-}$	1243.47
		[3M + 5TMA + 7Cl]$^{2-}$	1298.01
		[3M + 6TMA + 8Cl]$^{2-}$	1352.54
		[3M + 7TMA + 9Cl]$^{2-}$	1407.07
7	[4M + *n* TMA + (*n*+*1*)Cl]$^{-}$ (where *n* = *3*, *4* or higher)	Above 2000	Above 2000
8	[4M + *n* TMA + (*n*+*2*)Cl]$^{2-}$	[4M + TMA + 3Cl]$^{2-}$	1410.00

	(where *n* = *1, 2, 3, 4, 5, 6, 7, 8* or *9*)	$[4M + 2TMA + 4Cl]^{2-}$	1464.53
		$[4M + 3TMA + 5Cl]^{2-}$	1519.07
		$[4M + 4TMA + 6Cl]^{2-}$	1573.60
		$[4M + 5TMA + 7Cl]^{2-}$	1628.13
		$[4M + 6TMA + 8Cl]^{2-}$	1682.66
		$[4M + 7TMA + 9Cl]^{2-}$	1737.20
		$[4M + 8TMA + 10Cl]^{2-}$	1791.73
		$[4M + 9TMA + 11Cl]^{2-}$	1846.26
9	$[5M + n\ TMA + (n+1)Cl]^{-}$ (where *n* = *4, 5* or higher)	Above 2000	Above 2000
10	$[5M + n\ TMA + (n+2)Cl]^{2-}$ (where *n* = *2, 3, 4*, or *5*)	$[5M + 2TMA + 4Cl]^{2-}$	1794.65
		$[5M + 3TMA + 5Cl]^{2-}$	1849.18
		$[5M + 4TMA + 6Cl]^{2-}$	1903.72
		$[5M + 5TMA + 7Cl]^{2-}$	1958.25

^{1}H NMR Titration of Compound 1c with TBACl:

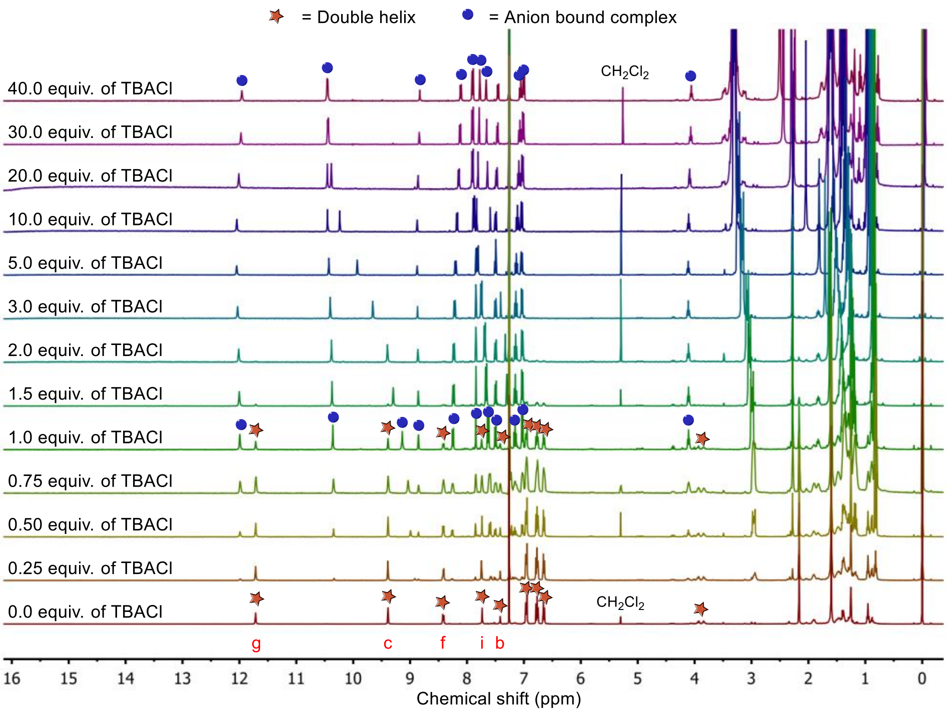

Supplementary Figure 29. The ^{1}H NMR titration experiment of 1c. The stacked plot of ^{1}H NMR titration of compound **1c** in $CDCl_3$ with the successive addition of TBACl at room temperature (400 MHz).

^{1}H NMR Titration of Compound 1c with TBABr, TBAI, and TBANO$_3$:

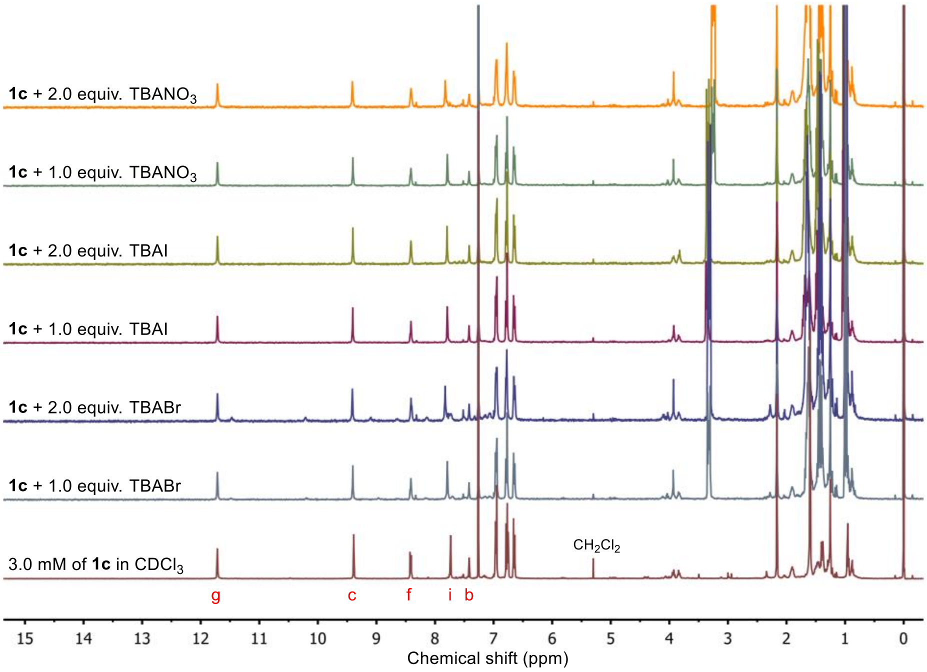

Supplementary Figure 30. The ^{1}H NMR titration experiment of 1c. The stacked plot of ^{1}H NMR of compound **1c** in $CDCl_3$ with different equivalents of TBABr, TBAI, and $TBANO_3$ at room temperature (400 MHz).

Anion Binding Reversibility of Anionic Complex by $AgBF_4$ and $AgPF_6$:

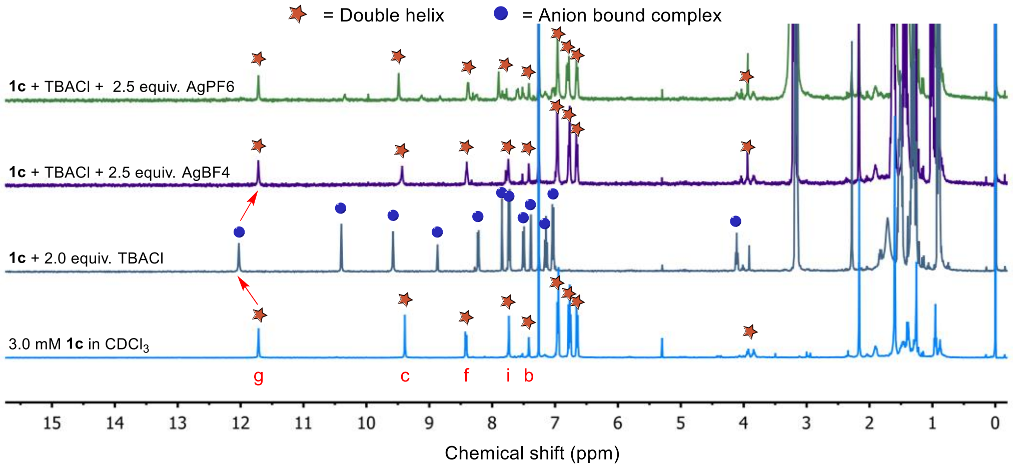

Supplementary Figure 31. The reversible behaviour of ^{1}H NMR titration experiment. The stacked plot of ^{1}H NMR of compound **1c** in $CDCl_3$ with the addition of 2 equivalents of TBACl and then the addition of 2.5 equivalents of silver salts for checking anion binding reversibility at room temperature (400 MHz).

The Cl^- Ion Binding Studies of Bis(indole) compound 1e:

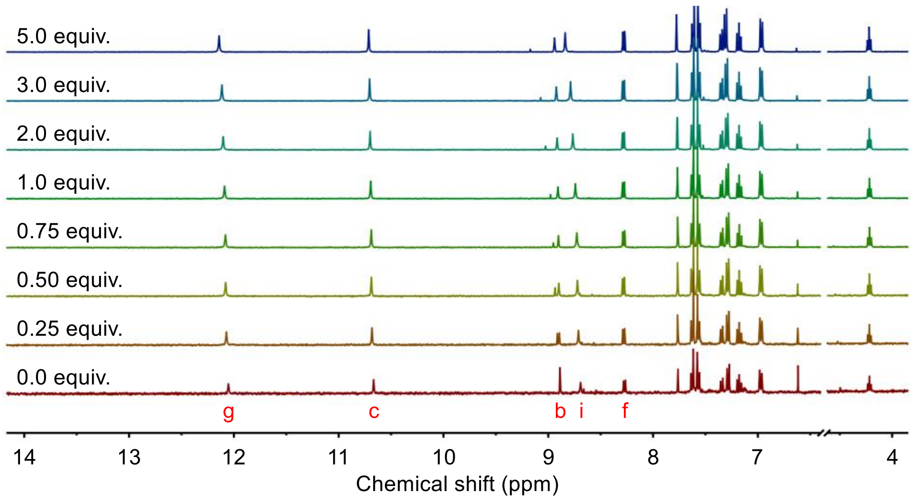

Supplementary Figure 32. The ^{1}H NMR titration experiment of 1e. The stacked plot of ^{1}H NMR spectra of **1e** (3.0 mM) in acetonitrile-d_3 at room temperature upon titrating increasing equivalents of TBACl.

UV-Vis titration Experiment for Anion Binding: The UV-Vis titration experiment was conducted in $C_2H_2Cl_4$ solvent system. The addition of TBACl salt to bis(indole) **1c** solution (10 μM) provided a significant intensity decrement of a shoulder peak at 352 nm of the UV-Vis absorption spectra (Supplementary Figure 33), indicating the formation of an anionic complex by the receptor molecule with the addition of Cl^-. The association constant was calculated by fitting the UV-Vis absorption data in the BindFit model (supramolecular.org) and was found to be 1.26×10^5 M^{-1} (± 10.7%).

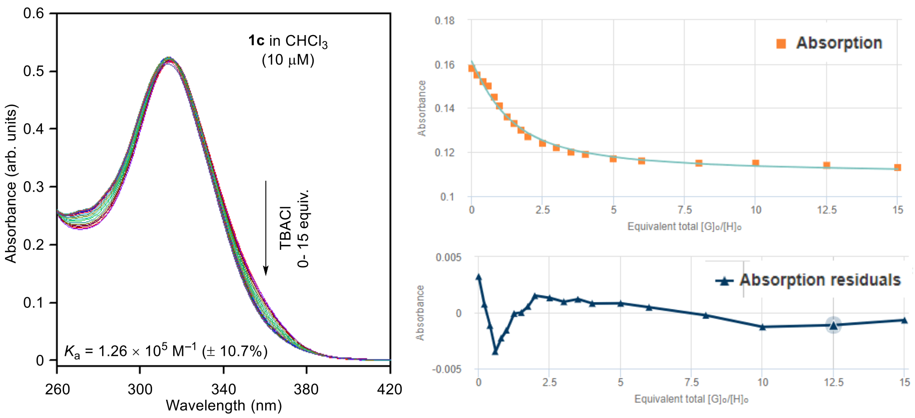

Supplementary Figure 33. The UV-Vis titration experiment of 1c with TBACl. The UV-Vis titration experiment of bis(indole) compound **1c** with the addition of TBACl in $C_2H_2Cl_4$ at 25 C (left side). Screenshot of the fitted data plot from supramolecular.org: The binding constant was found to be 1.26 × 10^5 (± 10.7%) M^{-1} in 1:1 receptor to anion binding model. The changing pattern of chemical shift and chemical shift residuals with the increasing equivalent TBACl (right side). The Bindfit URL for this experiment is: http://app.supramolecular.org/bindfit/view/42f6762e-221a-4e81-bbf6-994aefd74cca.

2D-DOSY NMR Experiment of Bis(indole) Compound 1c: To check the aggregation behaviour, the 2D-DOSY NMR experiments were performed for the free and anionic complex of the receptor $CHCl_3$ solution. At first 2D-DOSY NMR spectrum was recorded for the 5.0 mM solution of the free bis(indole) compound **1c**, which provided the diffusion coefficient value of 5.96 (± 0.03) × 10^{-6} cm^2/s (Supplementary Figure 34). The addition of 3.0 equivalent of TBACl salt in the same 5.0 mM receptor solution leads to the decrement of diffusion coefficient value, and was found to be 4.25 (± 0.02) × 10^{-6} cm^2/s (Supplementary Figure 35). This decrease in the diffusion coefficient value supports the polymer structure formation in the solution phase, which has formed by the conversion of the double helix form to the monomeric unit and subsequent formation of polymeric aggregates.

Further, the 2D-DOSY NMR spectra were also recorded at higher concentrations of the receptor with 3.0 equivalent of TBACl. The diffusion coefficient values were found to be 3.14 (± 0.02) × 10^{-6} cm^2/s and 2.64 (± 0.02) × 10^{-6} cm^2/s for 14 mM and 20 mM of receptor (with 3.0 equivalent of TBACl), respectively. Overall, in the presence of Cl^- ion, the diffusion coefficient values decreased from 4.25 (± 0.02) × 10^{-6} to 2.64 (± 0.02) × 10^{-6} cm^2/s with the increasing concentration of bis(indole) compound **1c**, from 5 to 20 mM (Supplementary Figure 36 and 37).

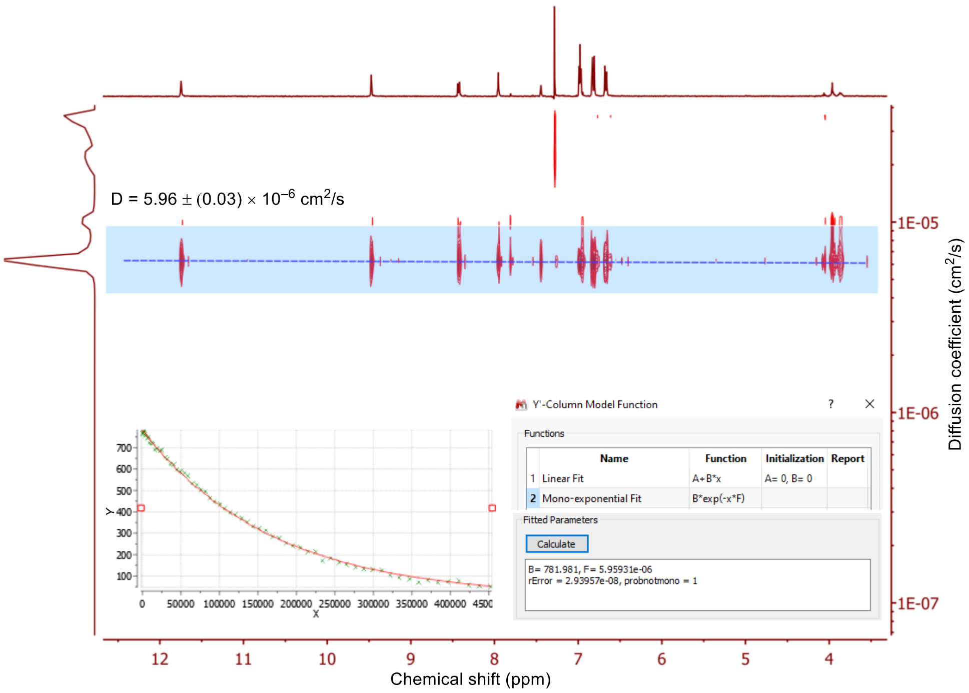

Supplementary Figure 34. The 2D DOSY NMR experiment of 1c. The 2D DOSY NMR spectrum of bis(indole) compound **1c** (5 mM) in $CHCl_3$ at 25 ºC. Two sub-figures in the bottom sections are the screenshots of the processed data. The diffusion coefficient value for this compound in the above-mentioned condition is 5.96 (± 0.03) × 10^{-6} cm^2/s.

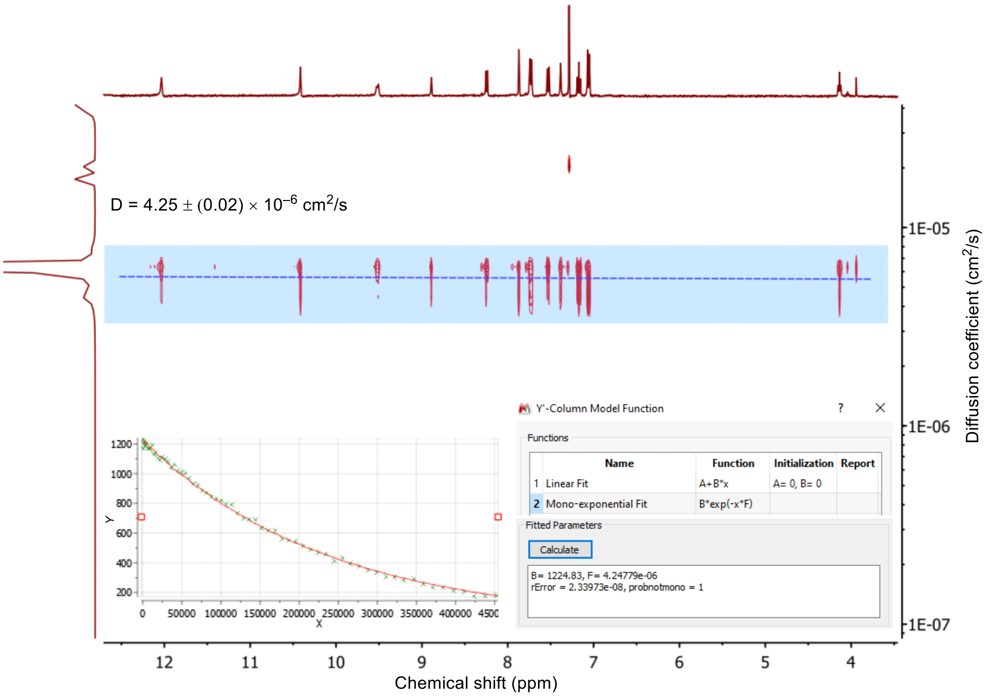

Supplementary Figure 35. The 2D DOSY NMR experiment of 1c with TBACl. The 2D DOSY NMR spectrum of bis(indole) compound **1c** (5 mM) with 3 equiv. of TBACl in $CHCl_3$ at 25 ºC. Two sub-figures in the bottom sections are the screenshots of the processed data. The diffusion coefficient value for this compound in the above-mentioned condition is 4.25 (± 0.02) × 10^{-6} cm^2/s.

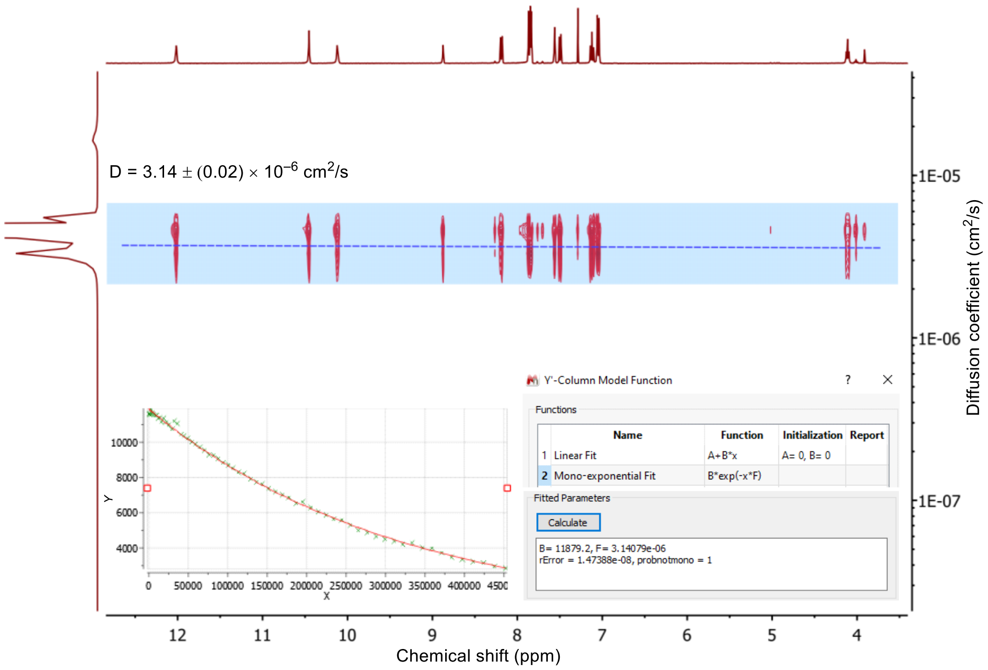

Supplementary Figure 36. The 2D DOSY NMR experiment of 1c with TBACl. The 2D DOSY NMR spectrum of bis(indole) compound **1c** (14 mM) with 3 equiv. of TBACl in $CHCl_3$ at 25 ºC. Two sub-figures in the bottom sections are the screenshots of the processed data. The diffusion coefficient value for this compound in the above-mentioned condition is 3.14 (± 0.02) × 10^{-6} cm^2/s.

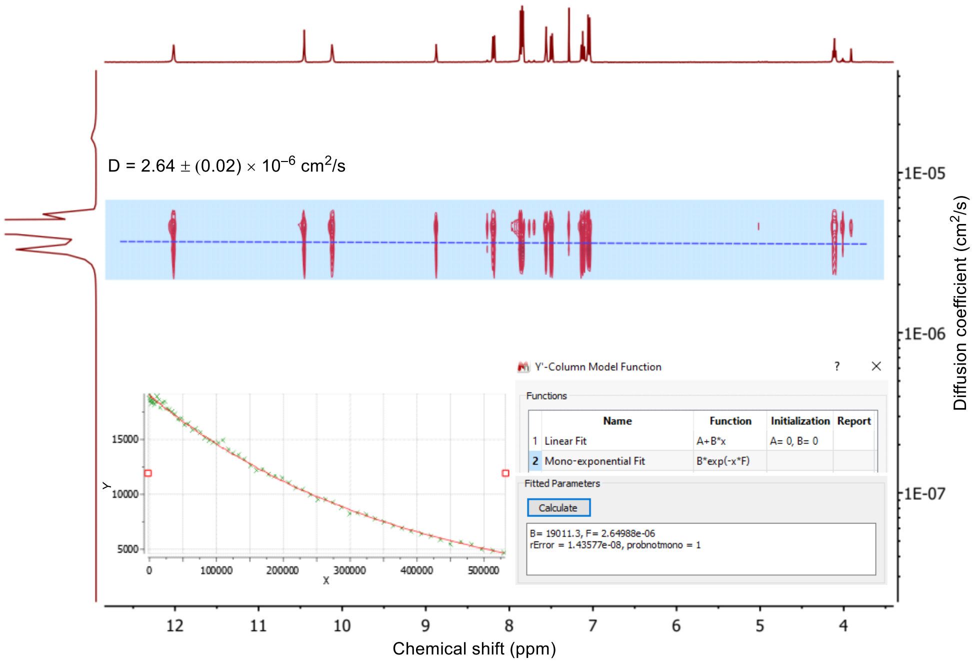

Supplementary Figure 37. The 2D DOSY NMR experiment of 1c with TBACl. The 2D DOSY NMR spectrum of bis(indole) compound **1c** (20 mM) with 3 equiv. of TBACl in $CHCl_3$ at 25 ºC. Two sub-figures in the bottom sections are the screenshots of the processed data. The diffusion coefficient value for this compound in the above-mentioned condition is 2.64 (± 0.02) × 10^{-6} cm^2/s.

HPTS Assay for Checking Ion Transport Activity: A salt solution was prepared using autoclaved water of strength 100 mM of NaCl and 10 mM of HEPES. Initially the pH of the solution was below 7.0 and to make the pH at around 7.0 required amount of NaOH (0.5 M) solution was added. Then HPTS solution of 1 mM was prepared from solid HPTS using the above mentioned buffer solution. The stock solution of the transporter for the HPTS assay was prepared using HPLC grade DMSO.

Vesicles Preparation for HPTS Assay: At first, 1 mL of EYPC lipid solution (25 mg/mL in chloroform) was taken in a 10 mL round bottomed flask. The chloroform present in the lipid solution was then evaporated by a slow stream of nitrogen gas while rotating the round bottomed flask to get a thin film of lipid inside it. Then the trace amount of chloroform present in the lipid was evaporated by drying it in high vacuum for about 5 h. The thin lipid film was hydrated with HPTS solution (1 mM HPTS, 10 mM HEPES, 100 mM NaCl, pH 7) and then vortexed for 5 times (2 minutes each time) with 10 minutes of rest in between, where the overall process takes around 1 hour. Then the hydrated vesicles suspension was subjected through 15 freeze-thaw cycles and extrusions was done for 19 times (must be odd number) using 100 nm polycarbonate membrane. The extravesicular dye was separated from vesicles by size exclusion column chromatography (using Sephadex G-50 gel) eluting with buffer solution (10 mM HEPES, 100 mM NaCl, pH 7). After collecting the vesicles from column were diluted to 6 mL by using aforementioned buffer (10 mM HEPES, 100 mM NaCl, pH 7) to get the concentration of ~ 5.5 mM of EYPC-LUVs⊃HPTS, assuming no loss of lipid throughout the process. The vesicles compositions, inside: 1 mM HPTS, 10 mM HEPES, 100 mM NaCl, pH 7 and outside: 10 mM HEPES, 100 mM NaCl, pH 7.

Description of HPTS Assay for Ion Transport Study: In a clean cuvette, 1975 µL of buffer solution (10 mM HEPES, 100 mM NaCl, pH 7), 25 µL of HPTS trapped vesicles solution were taken and placed in a fluorescence instrument equipped with a magnetic stirrer, which lead to the concentration of lipid is around 65 µM. The fluorescence emission intensity of the HPTS dye, I_t was measured at λ_{em} = 510 nm (where, λ_{ex} = 450 nm) for 350 s. For each reading, the start time of the instrument was considered as t = 0 s. Then at t = 20 s, 20 µL of 0.5 M NaOH solution was added to the same cuvette to generate a pH gradient (ΔpH = 0.8) between intra and extra vesicular medium. Then 20 µL solution of transporters in DMSO of different concentrations were added at t = 100 s. At t = 300 s, 10% triton X-100 (25 µL) was added to destroy all the vesicles for the destructing of the pH gradient.

For the preparation of the vesicles, 25 mg of EYPC lipid (Egg PC, Formula Weight = 770.123) has been used, which resulted 6 mL of vesicles stock solution/suspension of concentration ~ 5.4 mM, considering no significant loss of lipid during the vesicles preparation process. The fluorescence experiments were performed by suspending 25 μL of such stock solution in the 2 mL of buffer solution, which finally furnished the lipid concentration of ~ 65 μM.

The fractional emission intensity (in percentage), I_F (Supplementary Figure 38) was calculated after normalizing all the data using the following equation (Supplementary Equation 1).

$$\% \text{ of } I_F = \frac{(I_t - I_0)}{(I_\infty - I_0)} \times (100) \qquad \text{Supplementary Equation 1}$$

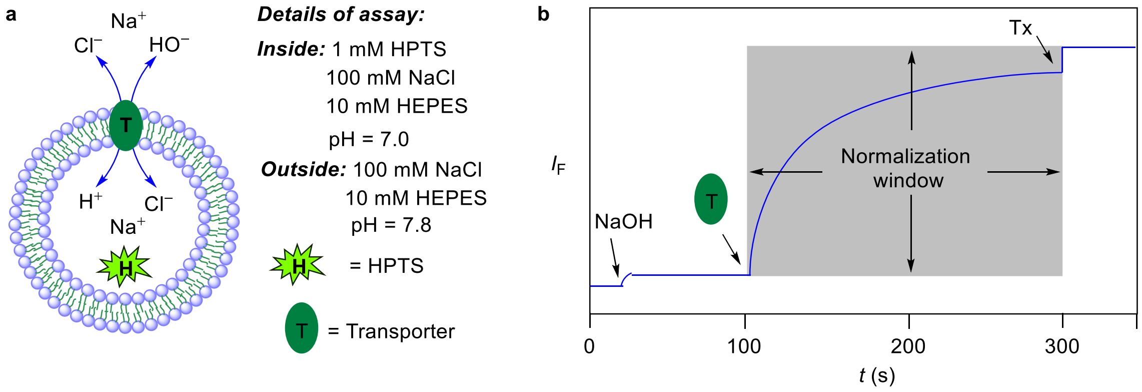

Supplementary Figure 38. Fluorescence kinetics assay. Schematic representation of fluorescence kinetics assay for checking ion transport activity across EYPC-LUVs⊃HPTS (**a**) and normalized working window for the same experiment (**b**).

Where, I_0 is the initial fluorescence intensity *i.e.* before the addition of the transporter compound, I_t is the fluorescence intensity at time t and I_∞ is the final fluorescence intensity *i.e.* after the addition of Triton X-100.

Before plotting the data the time axis was normalized using the following equation:

$$t = t - 100 \qquad \text{Supplementary Equation 2}$$

The concentration dependent ion transport activity data was used for fitting of the "Hill equation" (Supplementary Equation 3) to get the EC_{50} value *i.e.* half maximal effective concentration and Hill coefficient (n).

$$Y = Y_\infty + \frac{(Y_0 - Y_\infty)}{\left[1 + \left(\frac{c}{EC_{50}}\right)^n\right]} \qquad \text{Supplementary Equation 3}$$

Where, Y_0 is the initial fluorescence intensity *i.e.* before the addition of the transporter molecule, Y_∞ is the fluorescence intensity after addition of excess transporter molecule, and c is the concentration of the transporter in the same assay condition.

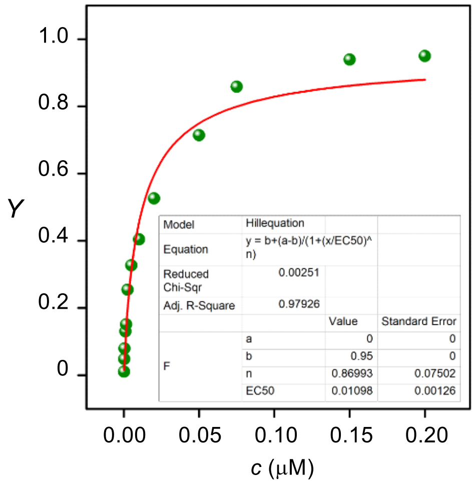

Supplementary Figure 39. The Hill plot for 1b. The Hill plot of transporter **1b** at t = 100 s to get EC_{50} and Hill coefficient.

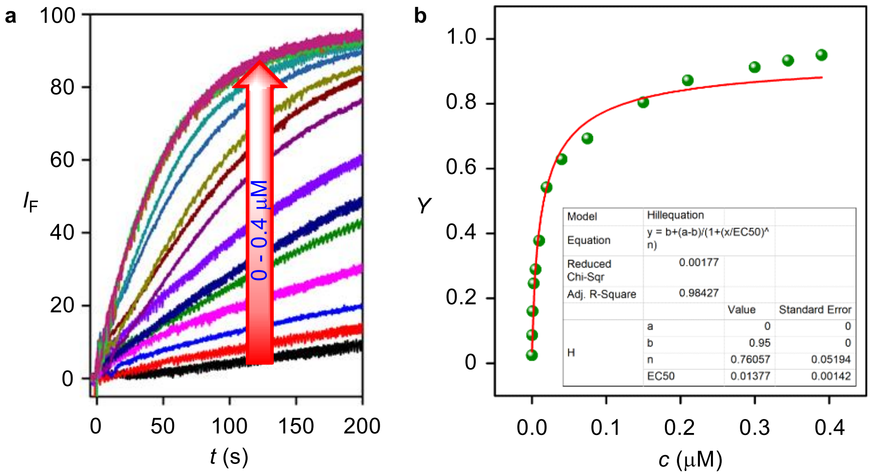

Supplementary Figure 40. The Hill plot for 1a. Concentration dependent ion transport activity assay of transporter **1a** (0–0.4 μM) across EYPC-LUVs⊃HPTS (**a**) and Hill plot of transporter **1a** at t = 100 s to get EC_{50} and Hill coefficient (**b**).

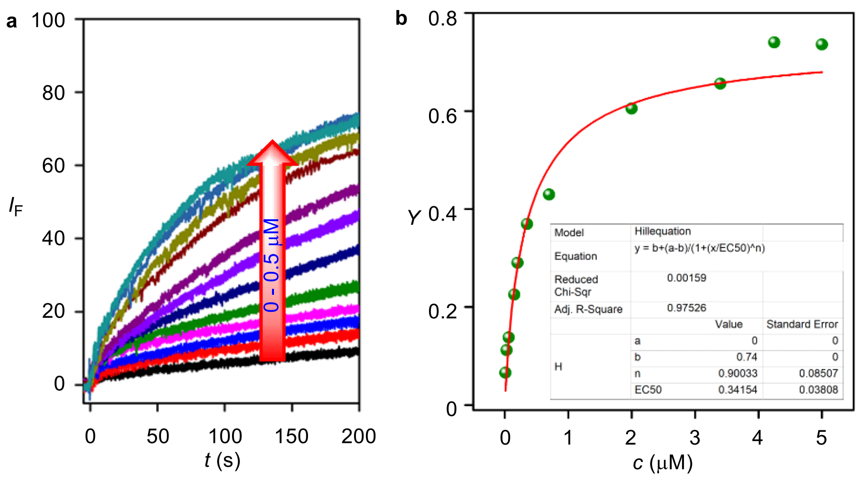

Supplementary Figure 41. The Hill plot for 1c. Concentration dependent ion transport activity assay of transporter **1c** (0–5 µM) across EYPC-LUVs⊃HPTS (**a**) and Hill plot of transporter **1c** at t = 100 s to get EC_{50} and Hill coefficient (**b**).

HPTS Assay for Free and Cl⁻ Ion Incorporated Stock Solution: In the above HPTS-based ion transport experiment, the transporter molecules were added (as a DMSO stock solution) to the vesicular solution containing 100 mM of NaCl solution and it was expected that the similar channel formation (like the crystal structure) will occur inside the hydrophobic lipid bilayer membrane with the help of Cl^- ions from the solution that eventually lead the transport of Cl^- ions across the bilayer membrane. To check the ion transport activity of the Cl^- ion incorporated channel-forming molecule, the stock solution was prepared with NaCl salt. For that in the above-mentioned stock solution NaCl solution (in water, 5 µL volume in 0.5 mL stock solution) was added and then the ion transport activity was checked using same volume of stock solution. From these experiments, no significant difference in the ion transport activity was observed.

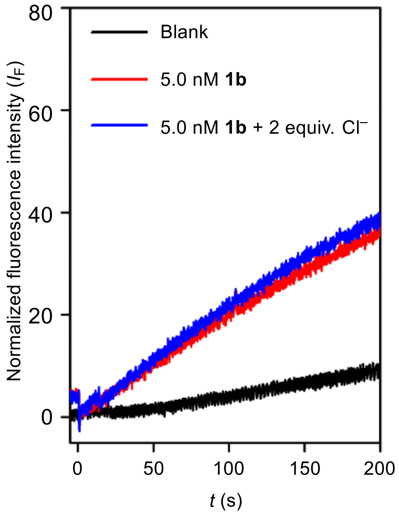

Supplementary Figure 42. The ion transport study in HPTS assay. The ion transport activity in HPTS assay for free and Cl^- ion incorporated stock solution of 5.0 nM of bis(indole) compound **1b**.

HPTS Assay for Checking Ion Selectivity: Different buffer solutions were prepared using autoclaved water of strength 100 mM of MX (where, MX = NaCl, NaBr, NaI, $NaNO_3$, NaOAc, LiCl, KCl, RbCl, CsCl) and 10 mM of HEPES. The pH of the solutions was adjusted to 7.0 by the addition of NaOH solution (0.5 M). The stock solution for the ion selectivity assay was prepared from solid transporter compound by using HPLC grade DMSO.

Vesicles Preparation for Ion Selectivity by HPTS Assay: The vesicles for the ion selectivity assay were prepared following the same procedure as mentioned earlier.

Description of HPTS Assay for Cation Selectivity: In a clean cuvette, 1975 µL of buffer solution (10 mM HEPES, 100 mM MCl, pH 7; where, M^+ = Na^+, Li^+, K^+, Rb^+, and Cs^+), 25 µL of HPTS trapped vesicles solution were taken and placed in a fluorescence instrument equipped with a magnetic stirrer. The fluorescence emission intensity of the HPTS dye, I_t was measured at λ_{em} = 510 nm (where λ_{ex} = 450 nm) for 350 s. The start time of the instrument for each reading was considered as t = 0 s. Then at t = 20 s, 20 µL NaOH solution (0.5 M) was added to the same cuvette to generate a pH gradient (ΔpH = 0.8) between intra and extra vesicular medium. Then 20 µL solution of the transporter in DMSO was added at t = 100 s. At t = 300 s, 10% triton X-100 (25 µL) was added to destroy all the vesicles for the

destructing of the pH gradient. All the data was normalized using the Supplementary Equation 1 and 2.

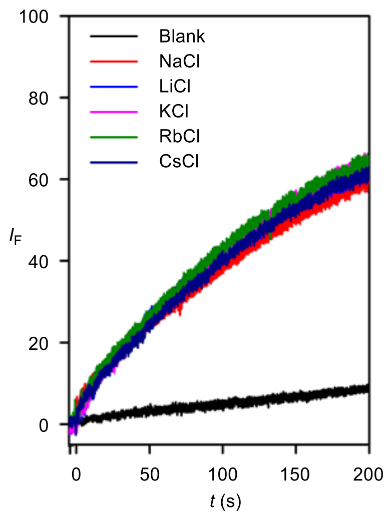

Supplementary Figure 43. The cation selectivity study. The ion transport activity by the transporter **1b** (12 nM) across EYPC-LUVs⊃HPTS by changing the extravesicular cations.

Description of HPTS Assay for Checking the pH Dissipation Trends: In a clean cuvette, 1975 µL of buffer solution (10 mM HEPES, 100 mM NaX, pH 7; where, X^- = Cl^-, Br^-, I^-, NO_3^-, ClO_4^-, SCN^-, and AcO^-), 25 µL of HPTS trapped vesicles solution were taken and placed in a fluorescence instrument equipped with a magnetic stirrer. The fluorescence emission intensity of the HPTS dye, I_t was measured at λ_{em} = 510 nm (where λ_{ex} = 450 nm) for 350 s. For each reading, the start time of the instrument was considered as t = 0 s. Then at t = 20 s, 20 µL NaOH solution (0.5 M) was added to the same cuvette to generate a pH gradient (ΔpH = 0.8) between intra and extra vesicular medium. Then 20 µL solution of the transporter in DMSO was added at t = 100 s. At t = 300 s, 10% triton X-100 (25 µL) was added to destroy all the vesicles for the destructing of the pH gradient. All the data was normalized using the Supplementary Equation 1 and 2.

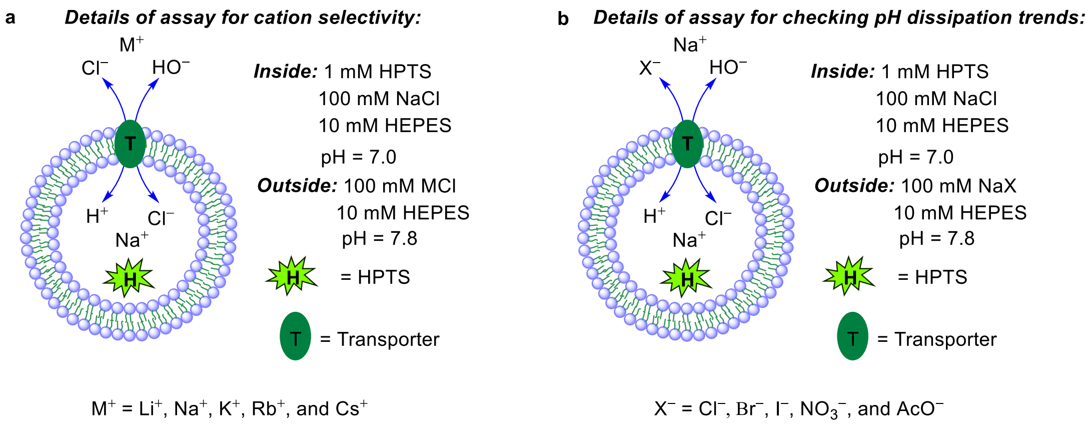

Supplementary Figure 44. The vesicles representation for selectivity study. Schematic representation of fluorescence kinetics assay for checking ion selectivity across EYPC-LUVs⊃HPTS. Cation selectivity assay (**a**) and pH dissipation trends (**b**).

Lucigenin Assay for Checking Cl^- Transport Activity: A 225 mM $NaNO_3$ salt solution was prepared using autoclaved water. Then 1 mM Lucigenin solution was prepared using the 225 mM $NaNO_3$ solution. The stock solution of the transporter for the Lucigenin assay was prepared from solid compound by using HPLC grade CH_3CN : CH_3OH (6 : 1) solution.

Vesicles Preparation Procedure for Lucigenin Assay: At first, 1 mL of EYPC lipid solution (25 mg/mL in chloroform) was taken in a 10 mL round bottomed flask. The chloroform present in the lipid solution was then evaporated by a slow stream of nitrogen gas while rotating the round bottomed flask to get a thin film of lipid inside it. Then the last trace amount of chloroform present in the lipid was evaporated by drying it in high vacuum for about 5 h. The thin film of lipid was hydrated with 1 mL of Lucigenin solution (1 mM Lucigenin, 225 mM $NaNO_3$) while vortexing 4-5 times by keeping 10 mins of gap in between it Then the hydrated vesicles suspension was subjected to 15 freeze-thaw cycles and extrusions was done for 19 times (must be odd number) using 200 nm polycarbonate membrane. The extravesicular dye was separated from vesicles by size exclusion column chromatography (using Sephadex G-50 gel) eluting with buffer solution (225 mM $NaNO_3$). After collecting the vesicles from column were diluted to 4 mL by using aforementioned buffer (225 mM $NaNO_3$) to get the concentration of ~8 mM of EYPC-LUVs⊃Lucigenin, assuming no loss of lipid throughout the process. The vesicles compositions, inside: 1 mM Lucigenin, 225 mM $NaNO_3$ and outside: 225 mM $NaNO_3$.

Concentration Dependent Cl⁻ Transport by Lucigenin Assay: In a clean cuvette, 1950 µL of salt solution (225 mM $NaNO_3$), 50 µL of lucigenin trapped vesicles solution were taken and placed in a fluorescence instrument equipped with a magnetic stirrer. The fluorescence emission intensity of the Lucigenin dye, I_t was measured at λ_{em} = 535 nm (where, λ_{ex} = 455 nm) for 350 s. For each reading, the start time of the instrument was considered as t = 0 s. Then at t = 50 s, 33 µL of 2N NaCl solution was added to the cuvette for generating a Cl^- concentration gradient across the lipid bilayer. Then 20 µL solution of transporters in ACN : MeOH (6 : 1) solvent of different concentrations were added at t = 100 s. At t = 300 s, 10% triton X-100 (25 µL) was added to destroy all the vesicles for the destructing of the Cl^- concentration gradient.

The fractional emission intensity (in percentage), I_F (Supplementary Figure 45) was calculated after normalizing all the data using the following equation (Supplementary Equation 4).

$$\% \text{ of } I_F = \frac{(I_t - I_0)}{(I_\infty - I_0)} \times (-100) \qquad \text{Supplementary Equation 4}$$

Where, I_0 is the initial fluorescence intensity *i.e.* just before the addition of the transporter solution, I_t is the fluorescence intensity at time t, and I_∞ is the final fluorescence intensity *i.e.* after the addition of Triton X-100.

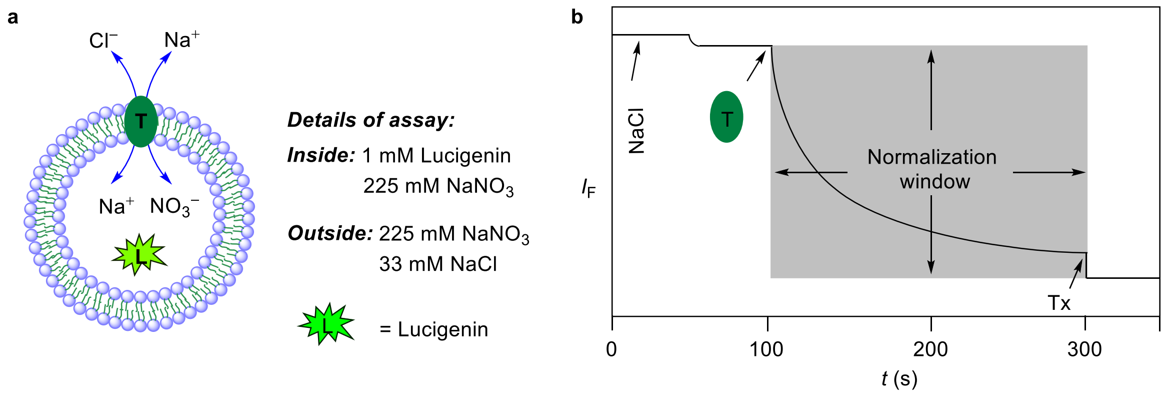

Supplementary Figure 45. The vesicles representation for Lucigenin assay. Schematic representation of fluorescence kinetics assay for checking Cl⁻ ion transport selectivity across EYPC-LUVs⊃Lucigenin (**a**) and normalized working window for the same experiment (**b**).

Before plotting the data the time axis was normalized using the Supplementary Equation 2, The concentration dependent ion transport activity data was used for fitting of the

“Hill equation” (Supplementary Equation 3) to get the EC_{50} value *i.e.* half maximal effective concentration and the Hill coefficient (*n*).

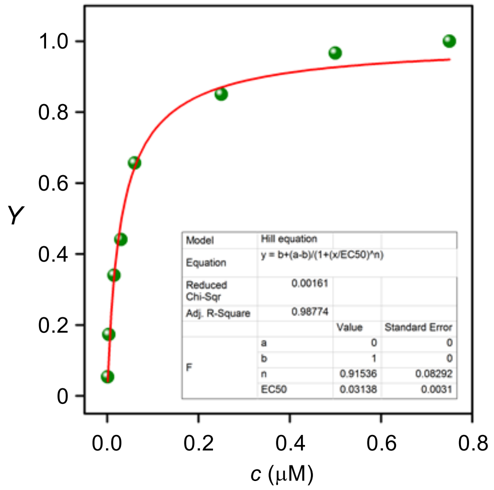

Supplementary Figure 46. The Hill plot for 1b. Hill plot of transporter **1b** at t = 100 s to get half maximal effective concentration (EC_{50}) and Hill coefficient.

Lucigenin Assay for Checking Symport Mechanism: In a clean cuvette, 1950 μL of salt solution (225 mM $NaNO_3$), 50 μL of lucigenin trapped vesicles solution were taken and placed inside the measurement compartment of a fluorescence instrument equipped with a magnetic stirrer. The fluorescence emission intensity of the Lucigenin dye, I_t was measured at λ_{em} = 535 nm by exciting at 455 nm for 350 s. For each reading, the start time of the instrument was considered as t = 0 s. Then at t = 50 s, 33 μL of 2 N MCl (where, $M^+ = Na^+$, Li^+, K^+, Rb^+, and Cs^+) was added to the cuvette for generating a Cl^- and M^+ concentration gradient across the lipid bilayer. Then 20 μL solution of transporters in ACN : MeOH (6 : 1) solvent was added at t = 100 s. At t = 300 s, 10% triton X-100 (25 μL) was added to destroy all the vesicles for the destructing of the Cl^- and M^+ concentration gradient.

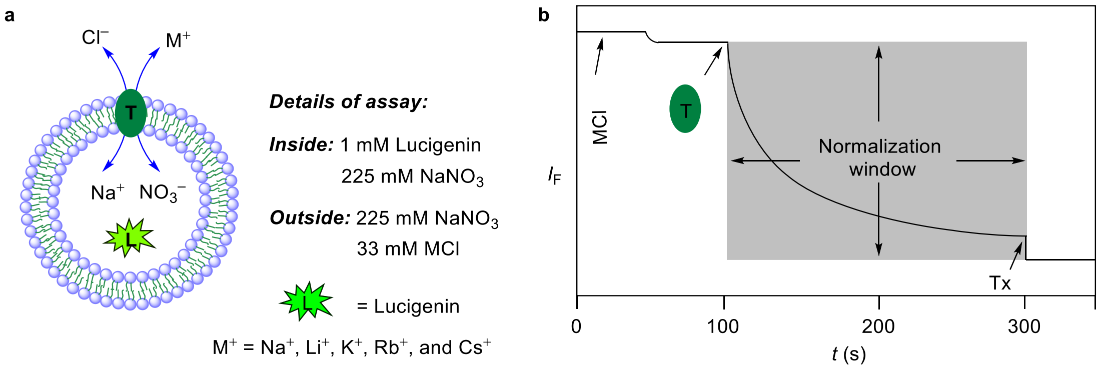

Supplementary Figure 47. The vesicles representation for Lucigenin assay. Schematic representation of fluorescence kinetics assay for checking symport mechanism across EYPC-LUVs⊃Lucigenin (**a**) and normalized working window for the same experiment (**b**).

Lucigenin Assay for Checking Antiport Mechanism in Presence of Valinomycin: In a clean cuvette, 1950 µL of salt solution (225 mM $NaNO_3$), 50 µL of lucigenin trapped vesicles solution were taken and placed inside the measurement compartment of a fluorescence instrument equipped with a magnetic stirrer. The fluorescence emission intensity of the Lucigenin dye, I_t was measured at λ_{em} = 535 nm by exciting at 455 nm for 350 s. For each reading, the start time of the instrument was considered as t = 0 s. Then at t = 50 s, 33 µL of 2 N KCl was added to the cuvette for generating a Cl^- and K^+ concentration gradient across the lipid bilayer. The valinomycin (0.5 µM) was added at t = 50 s. Then 20 µL solution of transporters in ACN : MeOH (6 : 1) solvent was added at t = 100 s. At t = 300 s, 10% triton X-100 (25 µL) was added to destroy all the vesicles for the destructing of the Cl^- and K^+ concentration gradient.

Before plotting the time axis was normalized using the Supplementary Equation 2.

In antiport assay, in the presence of valinomycin and KCl, the concentration gradient dissipation can occur in various possible way such as (a) Cl^-/NO_3^- antiport, (b) K^+/Cl^- symport by cooperative effect of transporter and valinomycin. However, the cooperative transport of K^+/NO_3^- symport will have comparably less effect, as the concentration of NO_3^- ion is the same in both the intra and extravesicular solution. The synergistic effect of valinomycin and the transporter enhanced the overall transport activity of the transporter in the presence of valinomycin, compared to the transporter alone. On the other hand, in the symport mechanism, a similar cooperative effect between transporter and valinomycin will not occur as the transporter would be self-sufficient in transporting K^+ along with Cl^- and thereby maintaining the electroneutrality. Therefore, the activity of the transporter molecule will be similar in the presence of valinomycin, compared to the transporter alone.

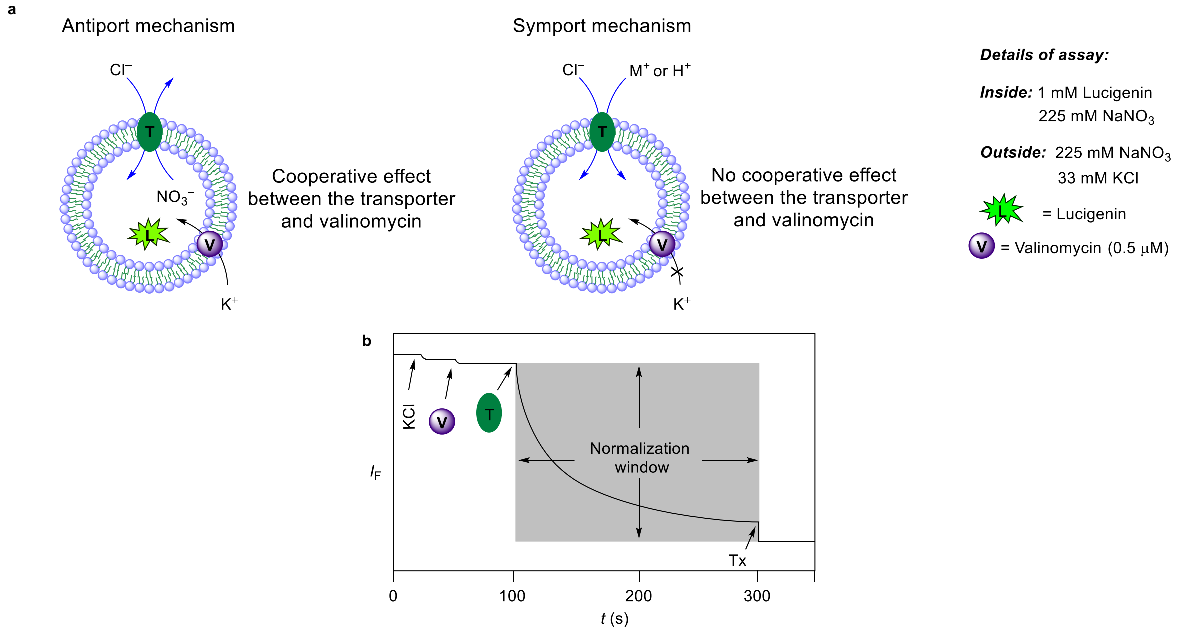

Supplementary Figure 48. The vesicles representation for Valinomycin assay. Schematic representation of fluorescence kinetics assay for checking antiport mechanism in presence of Valinomycin across EYPC-LUVs⊃Lucigenin (**a**) and normalized working window for the same experiment (**b**).

Planar Bilayer Conductance Measurement: The conductance measurement across the bilayer lipid membrane was done with the help of Warner instrument (USA). For that, a solution of diphytanoylphosphatidylcholine lipid (DPhPC; Avanti Polar Lipids) was prepared by dissolving it in *n*-decane of concentration 20 mg/mL. Then this solution was used to form a bilayer membrane across an aperture of 150 µm diameter in a polystyrene cup (Warner Instrument, USA). Both the *cis* and *trans* chambers of the instrument were filled with symmetrical solution, containing 1 M KCl. The *trans* chamber was held at virtual ground whereas the *cis* chamber was connected to the BC *535* head–stage (Warner Instrument, USA) via matched Ag-AgCl electrodes. Then the channel forming molecule **1b** was added (15 µM) to the trans chamber and the solution was stirred for with a inbuilt magnetic stirred for 20 min. The formation of the channel through the lipid bilayer was conformed from channel opening and closing events while applying different voltages using pClamp9 software (Molecular probes, USA). The currents were low pass filtered at 1 kHz using pClamp9 software (Molecular probes, USA) and analog-to-digital converter (Digidata 1440A, Molecular Devices).

The channel opening and closing events at positive as well as negative potentials were analysed by pClamp software (v. 10.6). A complete trace comprised of a series of opening

and closing events for the time period of 2 h from where a small fraction is presented in the manuscript.

The channel diameter was calculated using the equation (Hille equation) mentioned below.

$$\frac{1}{g} = \left(l + \frac{\pi d}{4}\right) \times \left(\frac{4\rho}{\pi d^2}\right) \qquad \text{Supplementary Equation 5}$$

Where, g = corrected conductance (calculated by multiplying measured single channel conductance with the Sansom's correction factor), l = length of the ion channel (34 Å), and ρ = resistivity of the KCl solution (ρ = 10.1 Ω·cm).

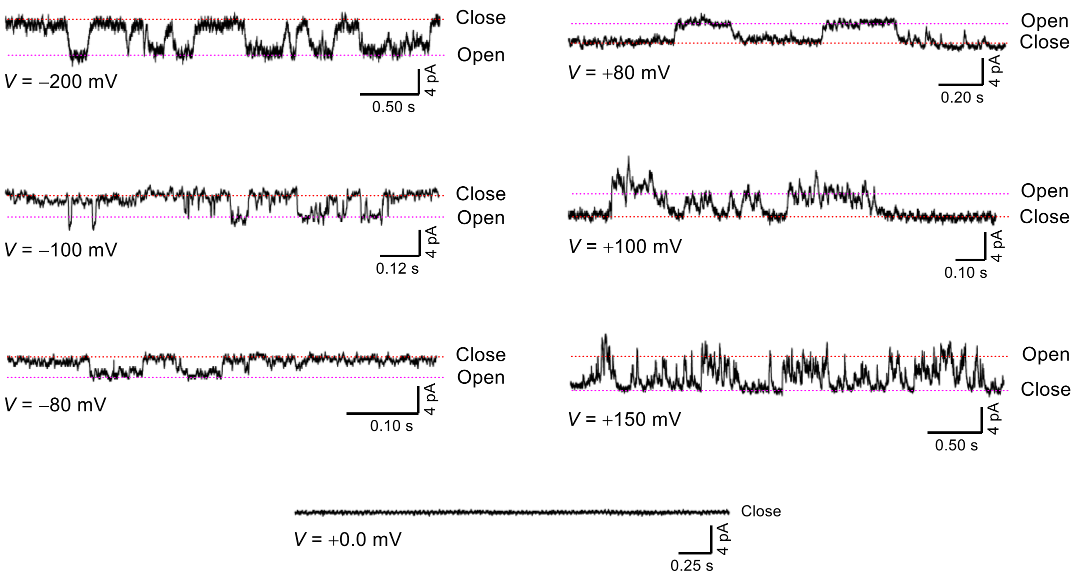

Supplementary Figure 49. Conductance measurement experiment. The single channel current traces by **1b** (15 µM) recorded at various negative and positive potentials under symmetrical KCl solution.

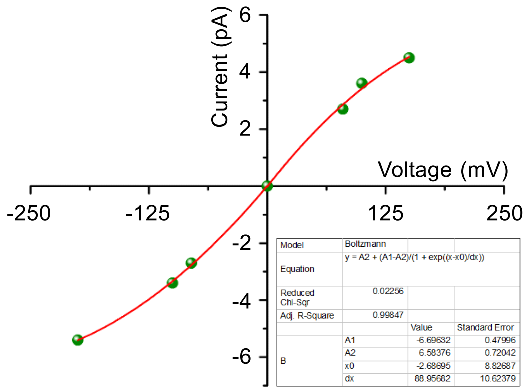

Model	Boltzmann		
Equation	y = A2 + (A1-A2)/(1 + exp((x-x0)/dx))		
Reduced Chi-Sqr	0.02256		
Adj. R-Square	0.99847		
		Value	Standard Error
B	A1	-6.69632	0.47996
	A2	6.58376	0.72042
	x0	-2.68695	8.82687
	dx	88.95682	10.62379

Supplementary Figure 50. The I–V plot from conductance measurement experiment. The plot current traces vs voltage obtained from the channel opening data at different potentials of **1b** fit in sigmoidal equation.

Supramolecular Channel Formation Proposed Model: To support the supramolecular channel formation inside the bilayer membrane the following schematic explanation is used.

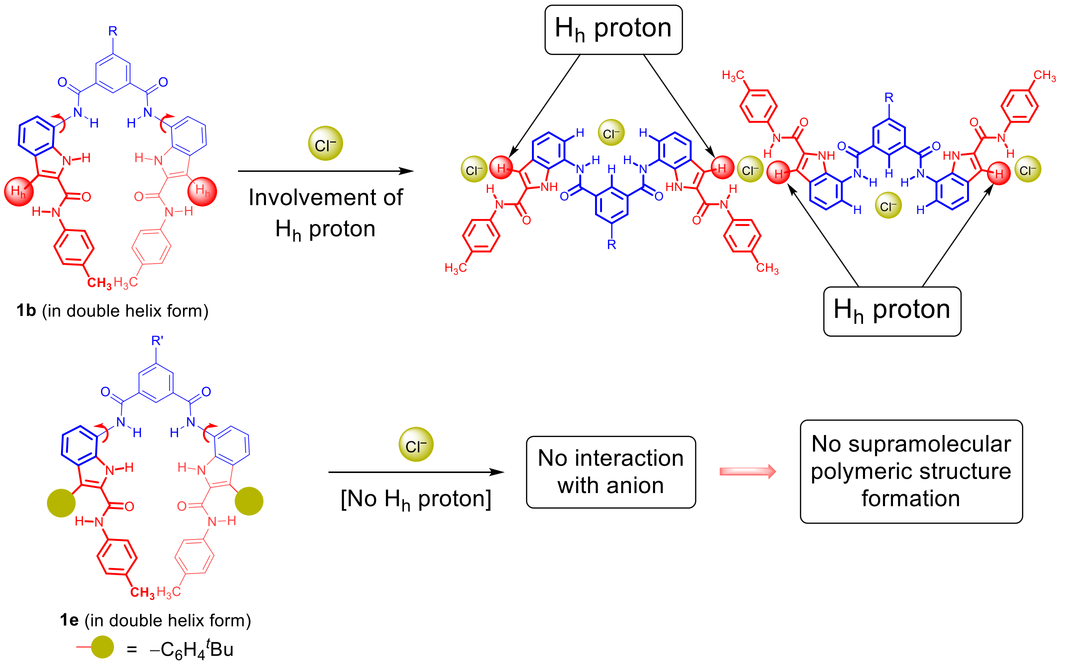

Supplementary Figure 51. The Polymer formation strategy. Proposed schematic representation of supramolecular polymer formation involving the H_h proton.

NMR Spectra of Compounds:

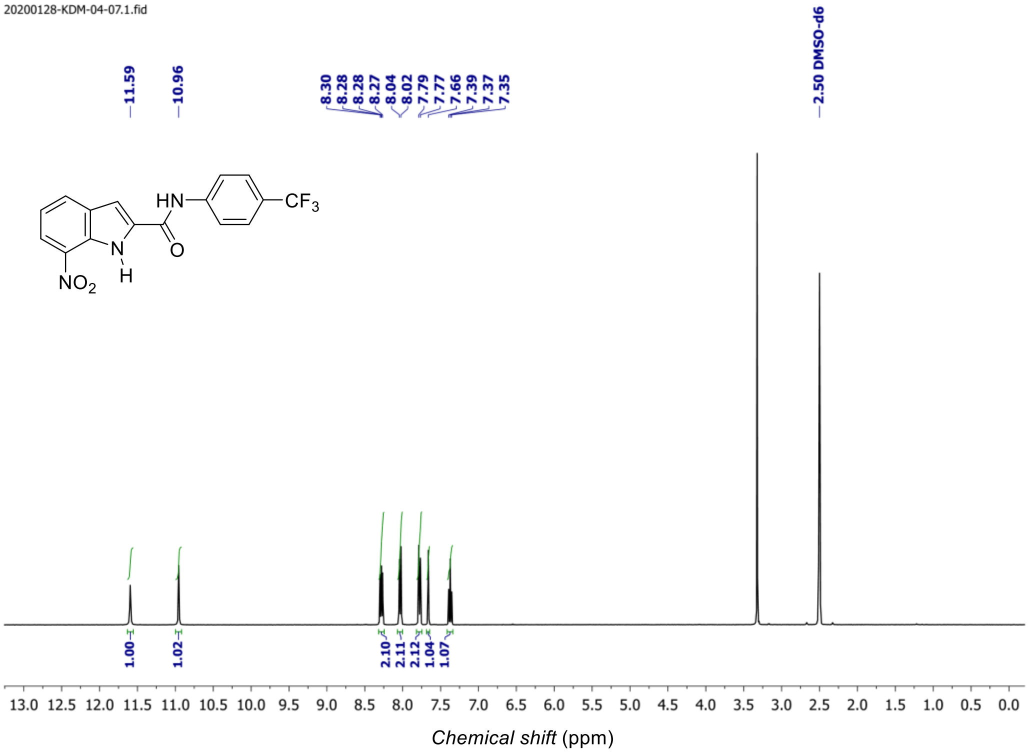

Supplementary Figure 52. ^{1}H NMR spectrum of compound **9a** in DMSO-d_6.

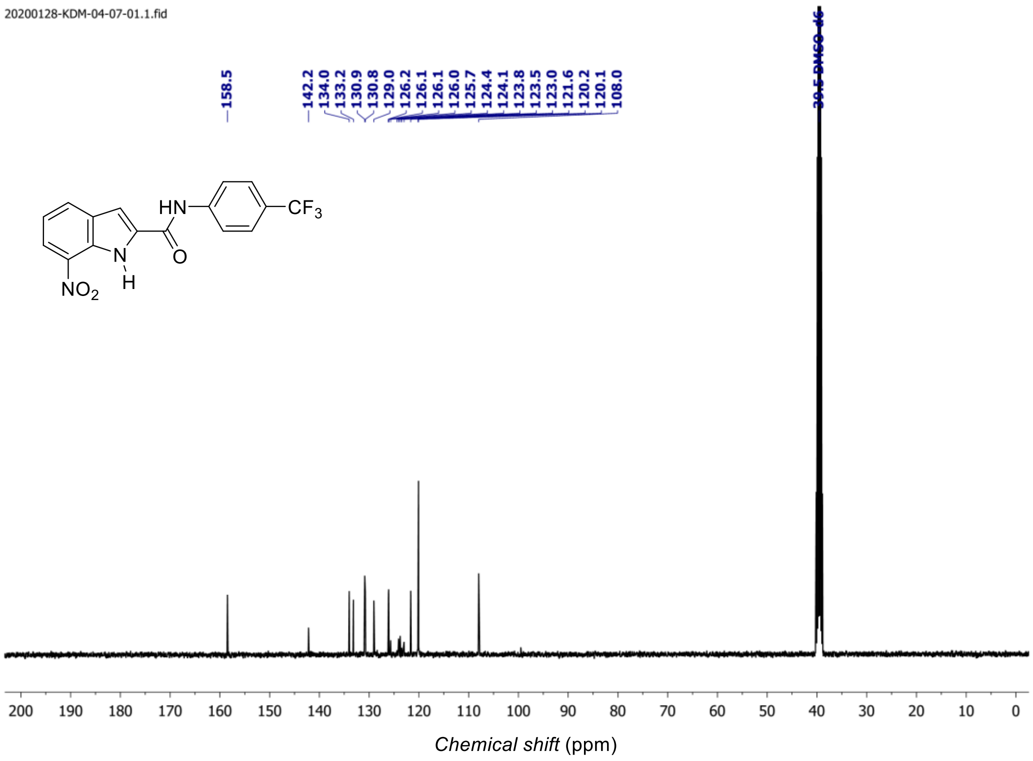

Supplementary Figure 53. ^{13}C NMR spectrum of compound **9a** in DMSO-d_6.

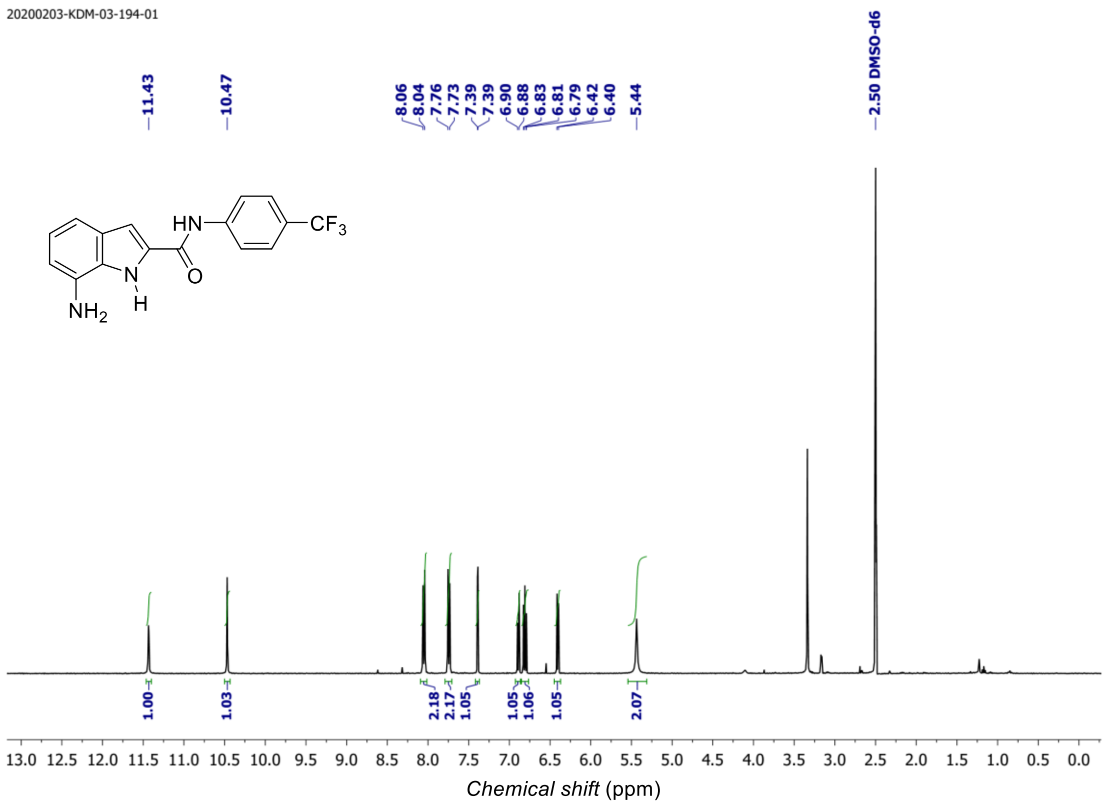

Supplementary Figure 54. ^{1}H NMR spectrum of compound **10a** in DMSO-d_6.

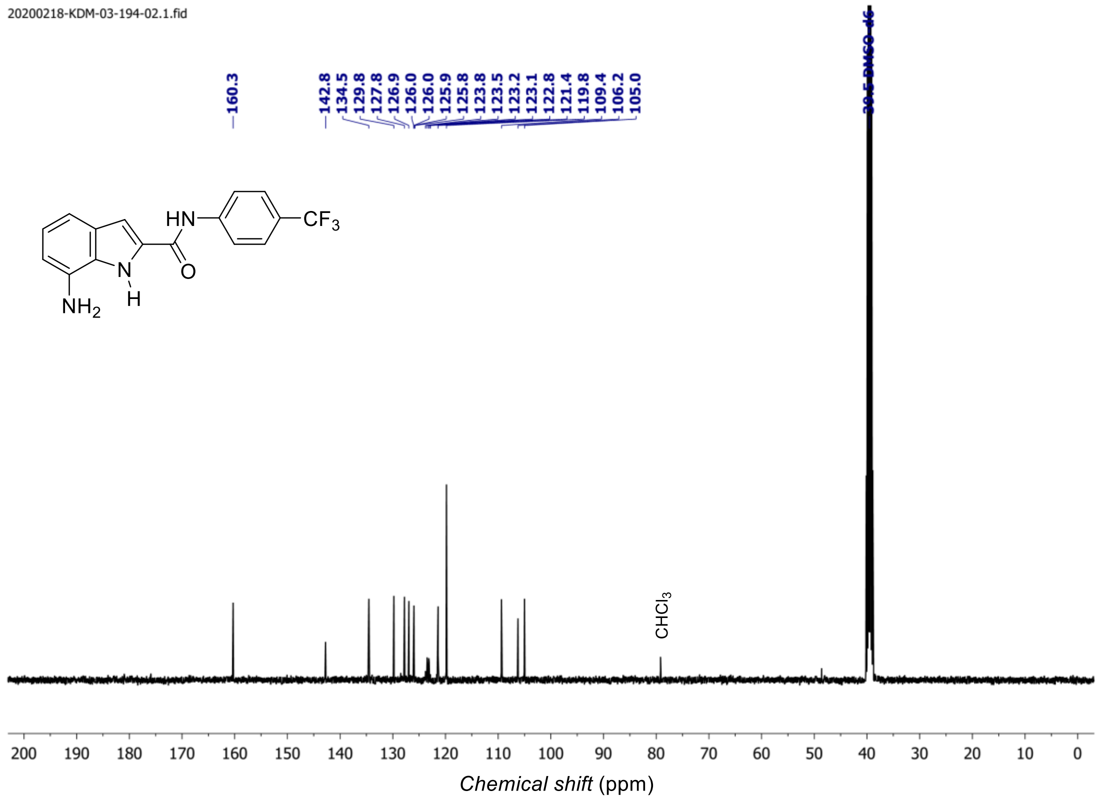

Supplementary Figure 55. ^{13}C NMR spectrum of compound **10a** in DMSO-d_6.

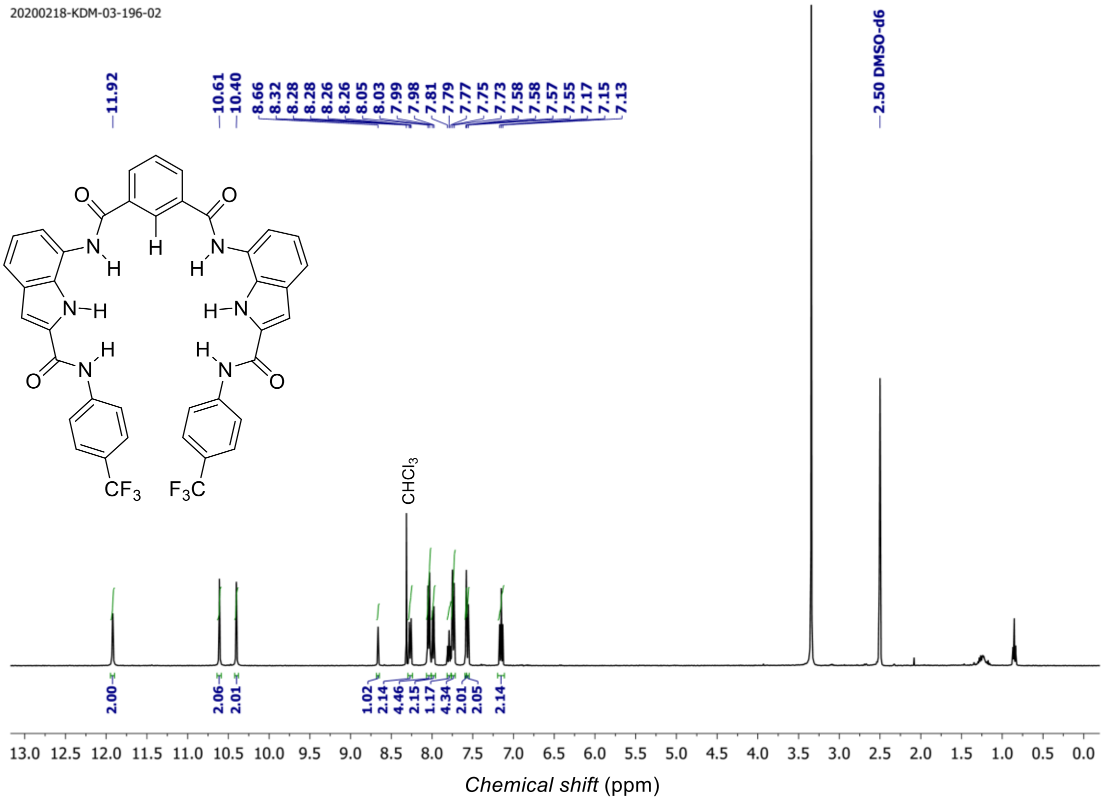

Supplementary Figure 56. ^{1}H NMR spectrum of compound **1a** in DMSO-d_6.

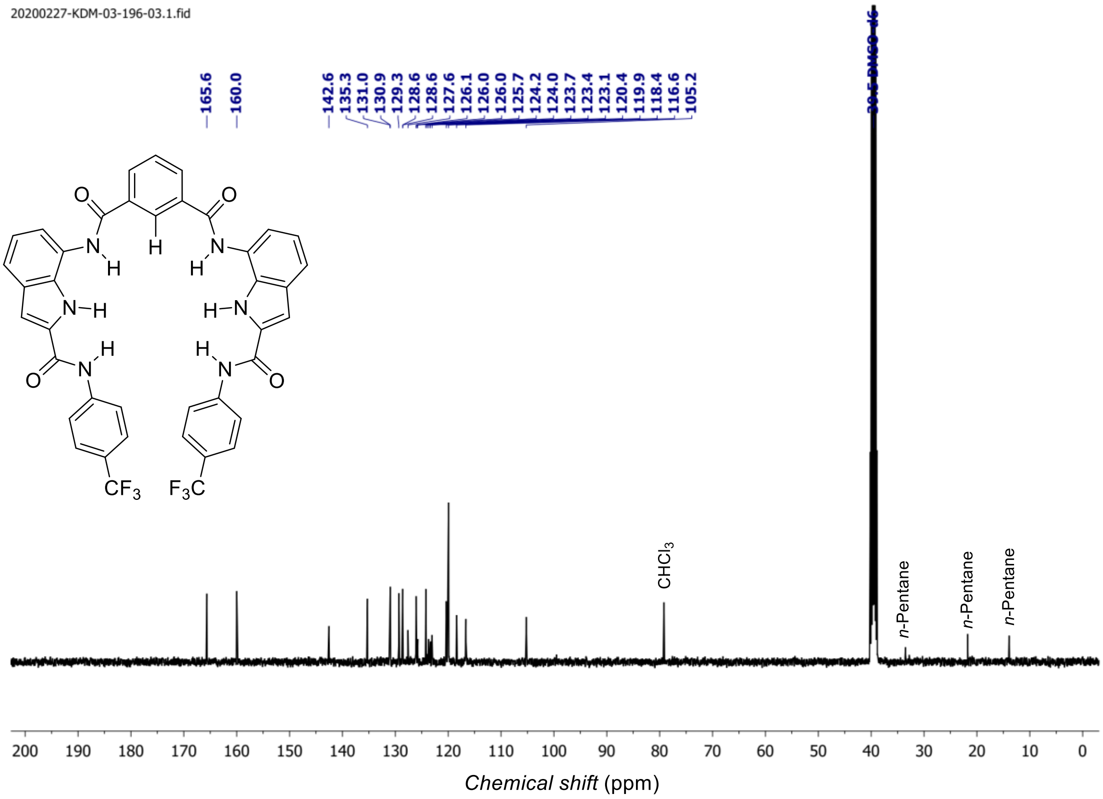

Supplementary Figure 57. ^{13}C NMR spectrum of compound **1a** in DMSO-d_6.

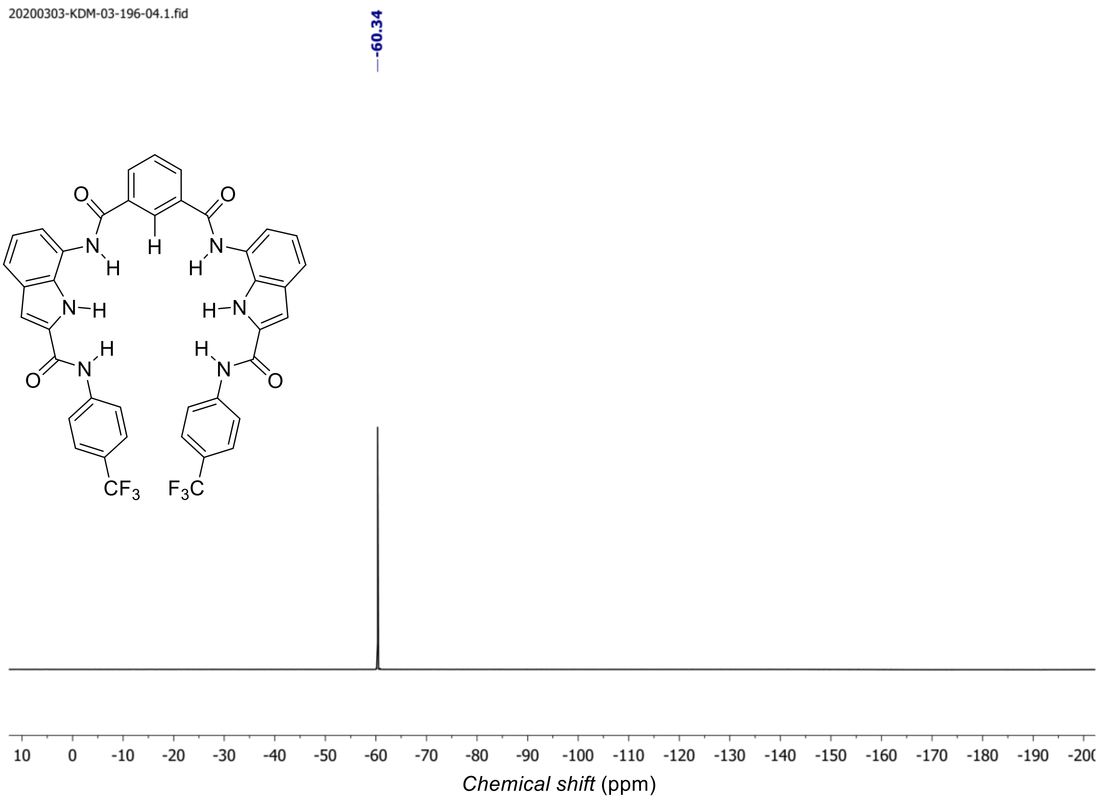

Supplementary Figure 58. ^{19}F NMR spectrum of compound **1a** in DMSO-d_6.

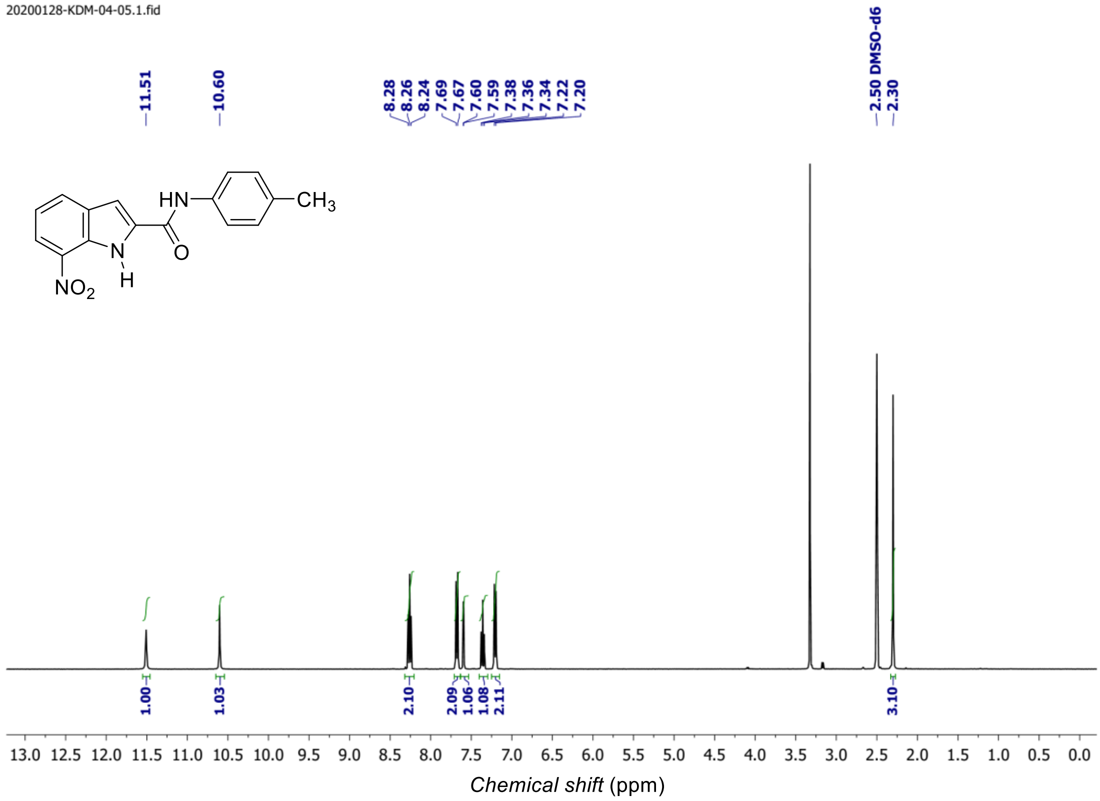

Supplementary Figure 59. ^{1}H NMR spectrum of compound **9b** in DMSO-d_6.

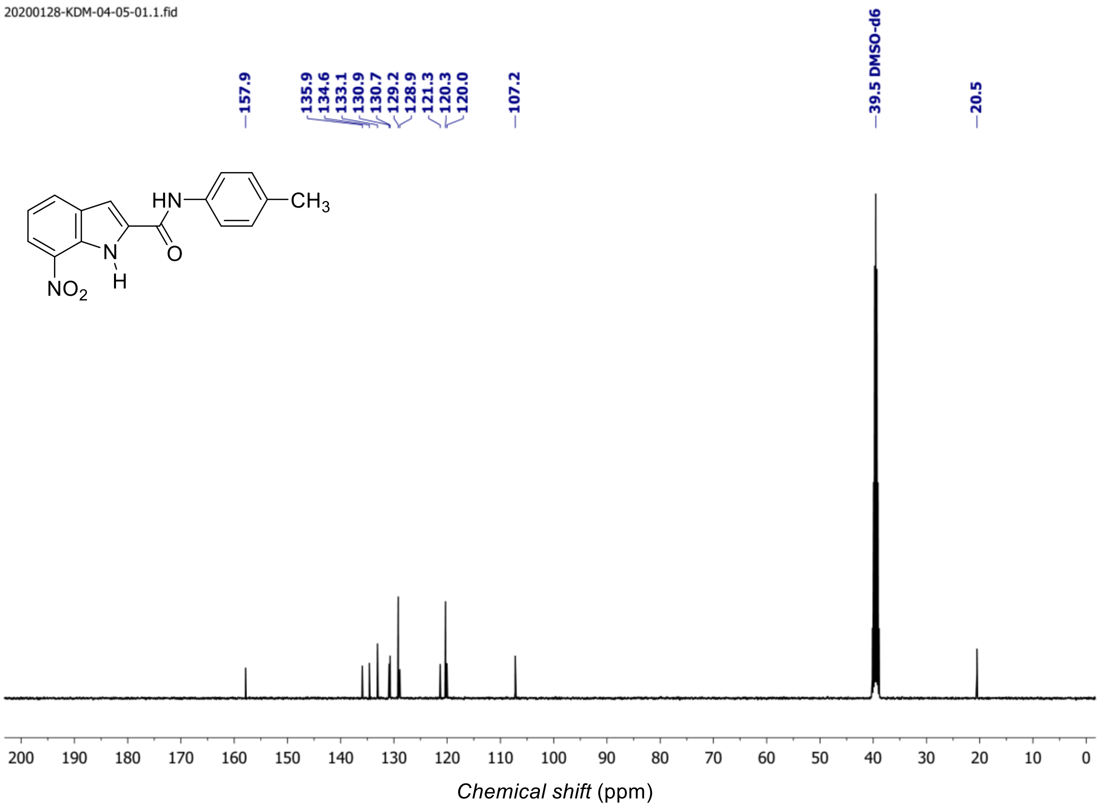

Supplementary Figure 60. ^{13}C NMR spectrum of compound **9b** in DMSO-d_6.

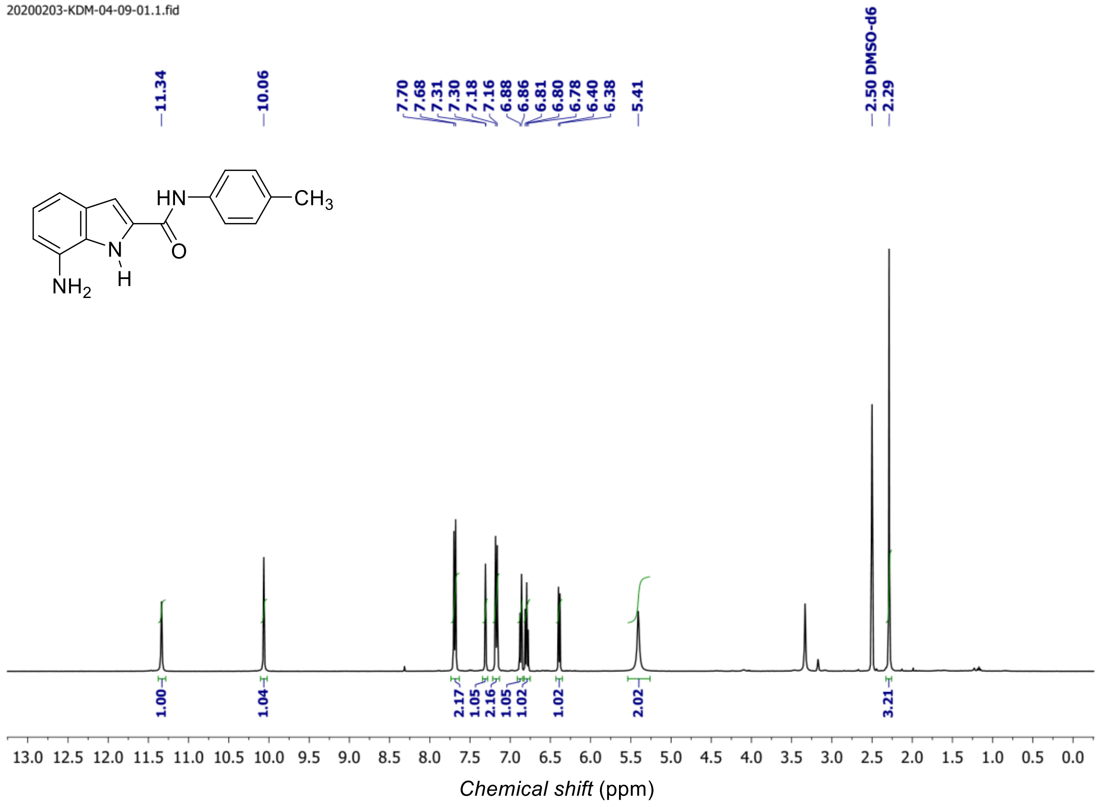

Supplementary Figure 61. ^{1}H NMR spectrum of compound **10b** in DMSO-d_6.

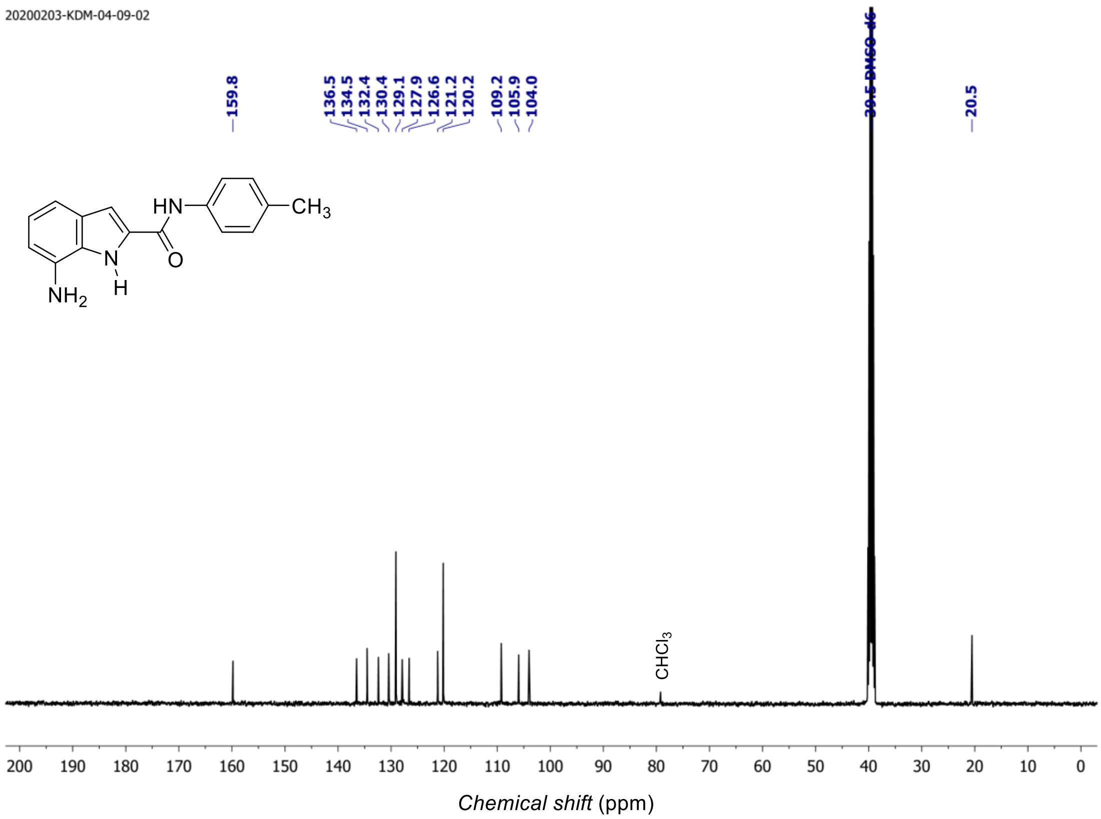

Supplementary Figure 62. ^{13}C NMR spectrum of compound **10b** in DMSO-d_6.

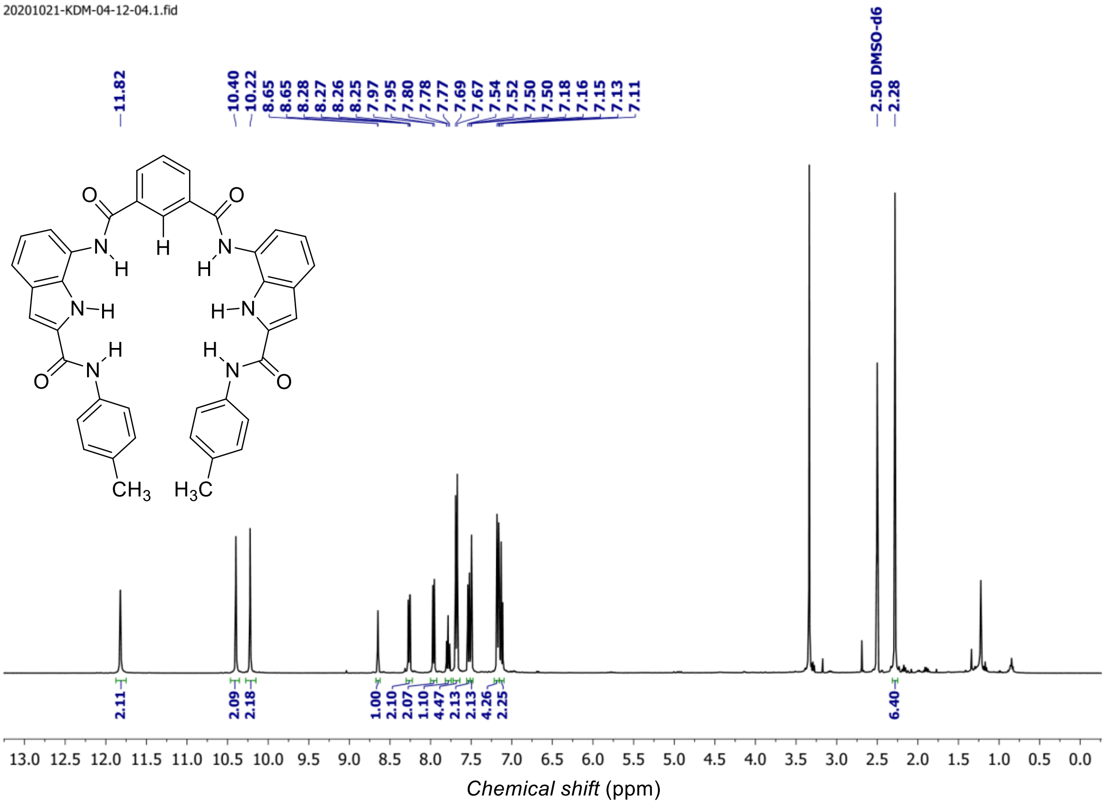

Supplementary Figure 63. ^{1}H NMR spectrum of compound **1b** in DMSO-d_6.

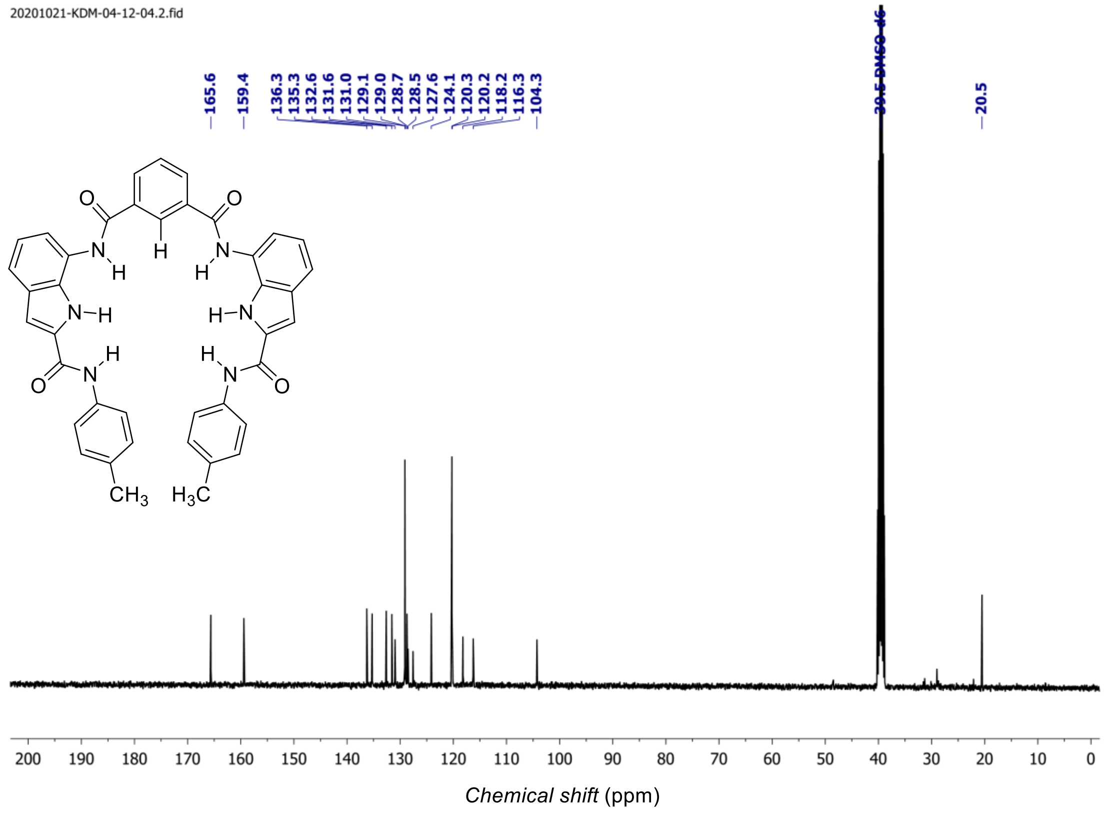

Supplementary Figure 64. ^{13}C NMR spectrum of compound **1b** in DMSO-d_6.

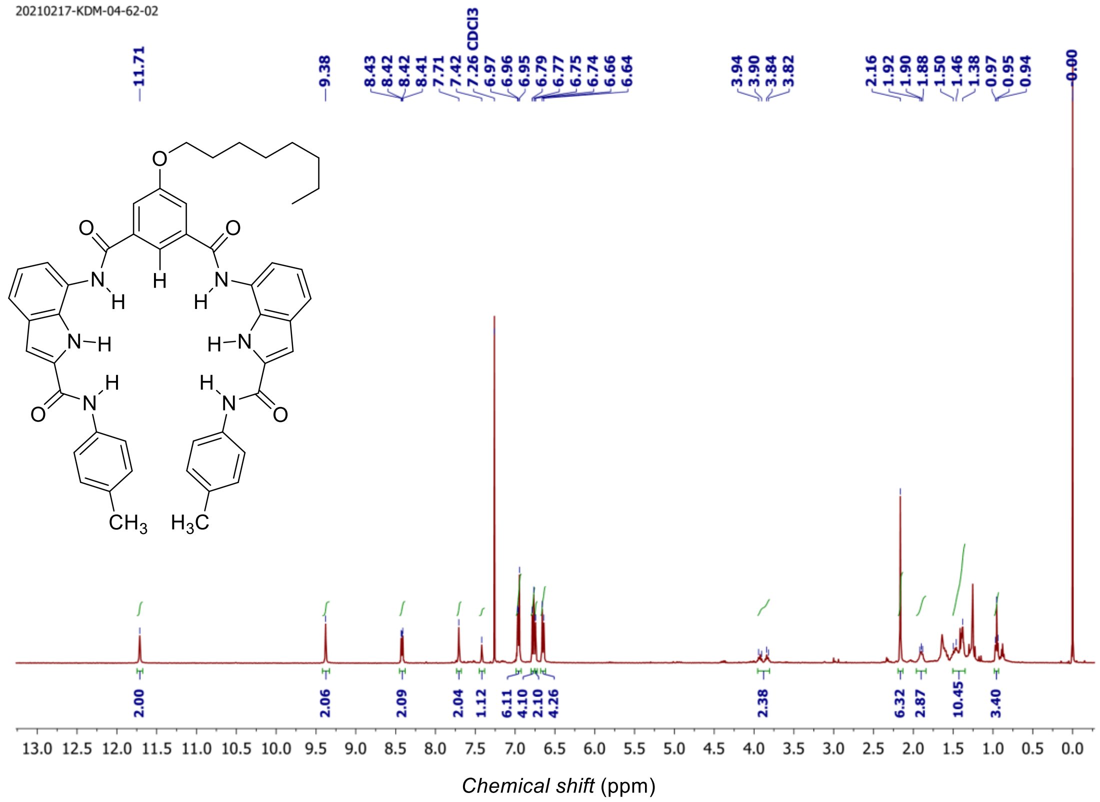

Supplementary Figure 65. ^{1}H NMR spectrum of compound **1c** in $CDCl_3$.

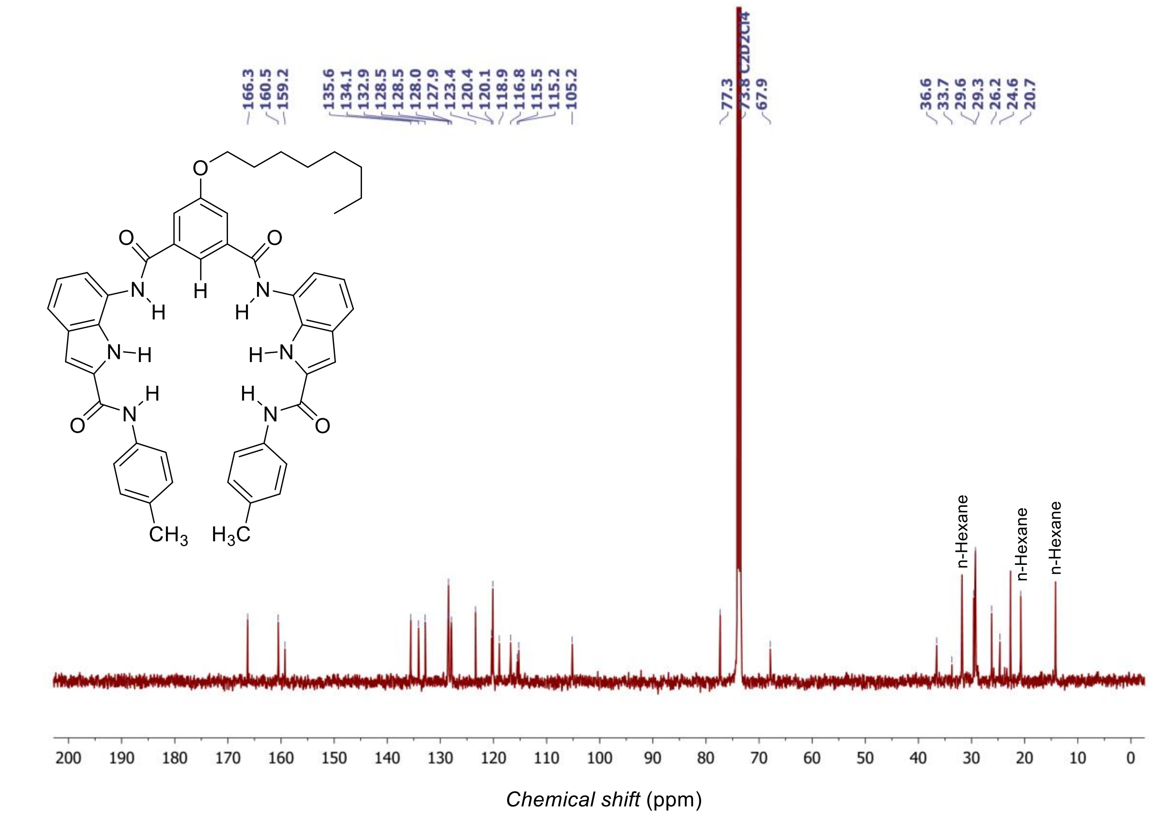

Supplementary Figure 66. ^{13}C NMR spectrum of compound **1c** in $C_2D_2Cl_4$.

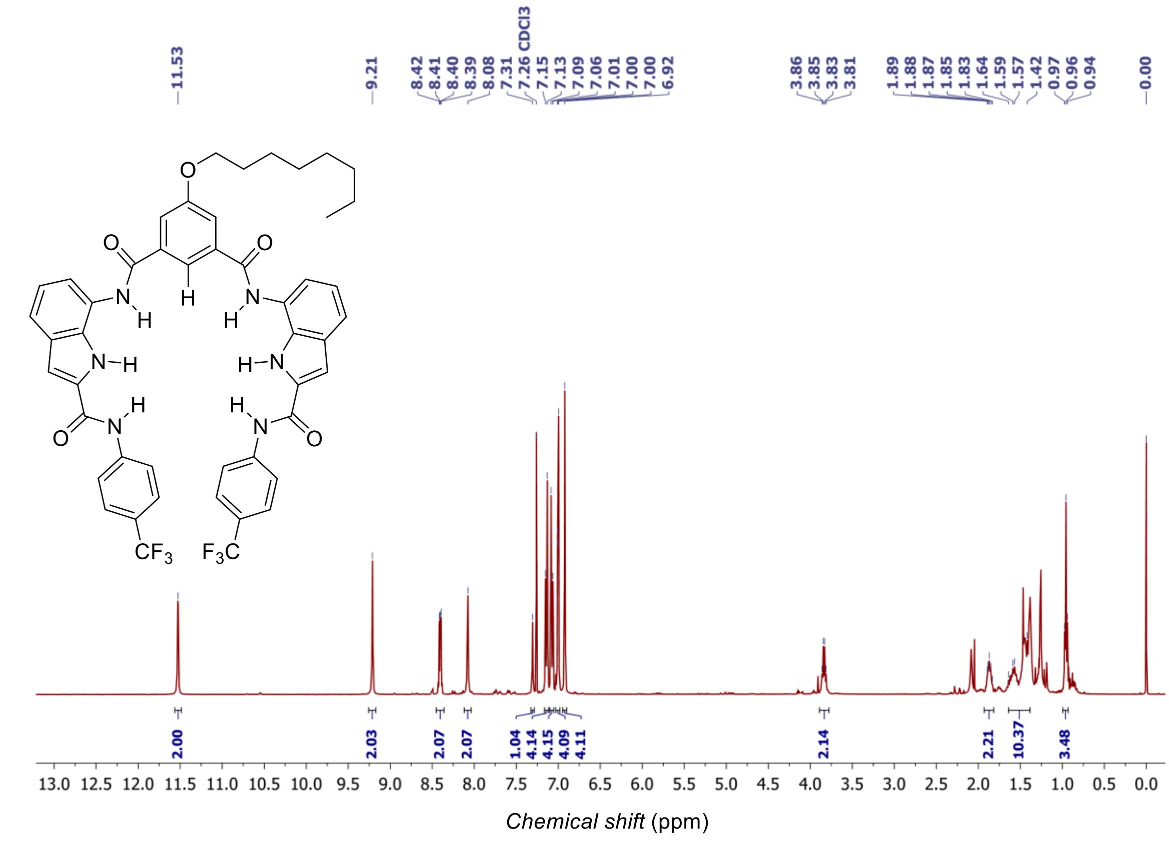

Supplementary Figure 67. ^{1}H NMR spectrum of compound **1d** in $CDCl_3$.

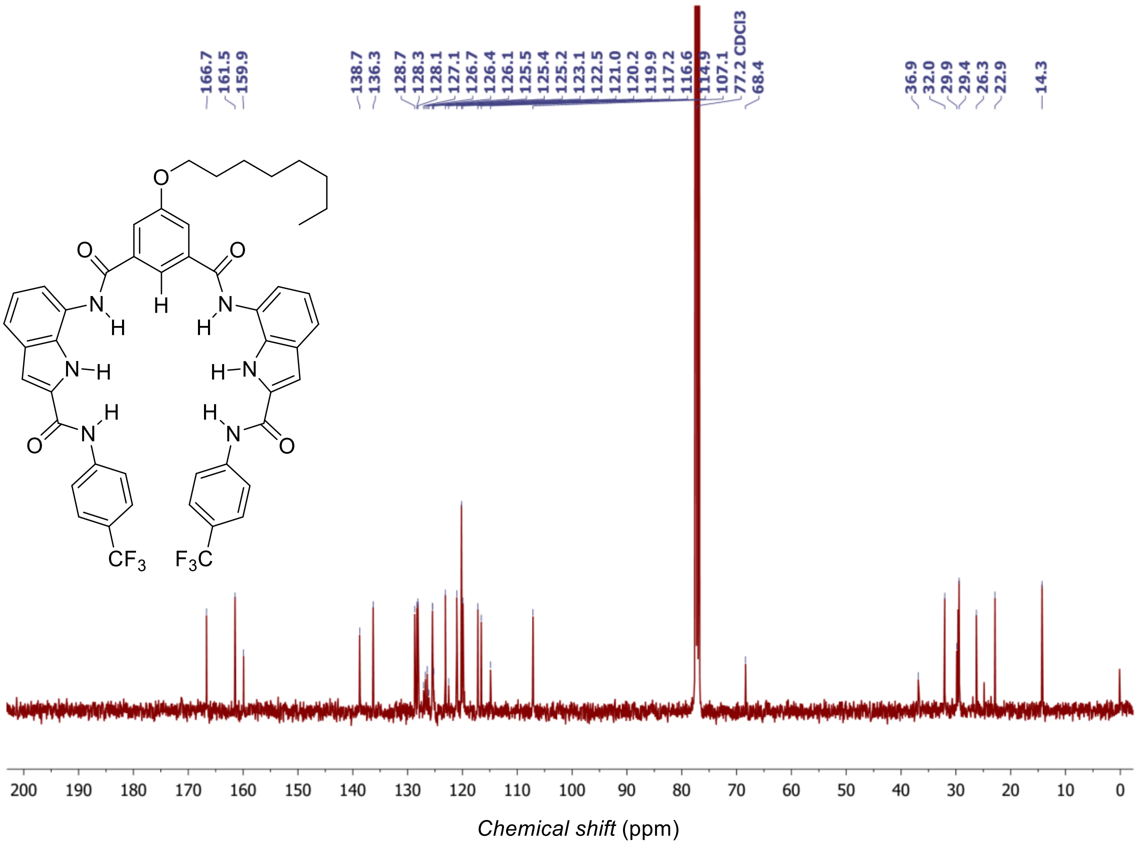

Supplementary Figure 68. ^{13}C NMR spectrum of compound **1d** in $CDCl_3$.

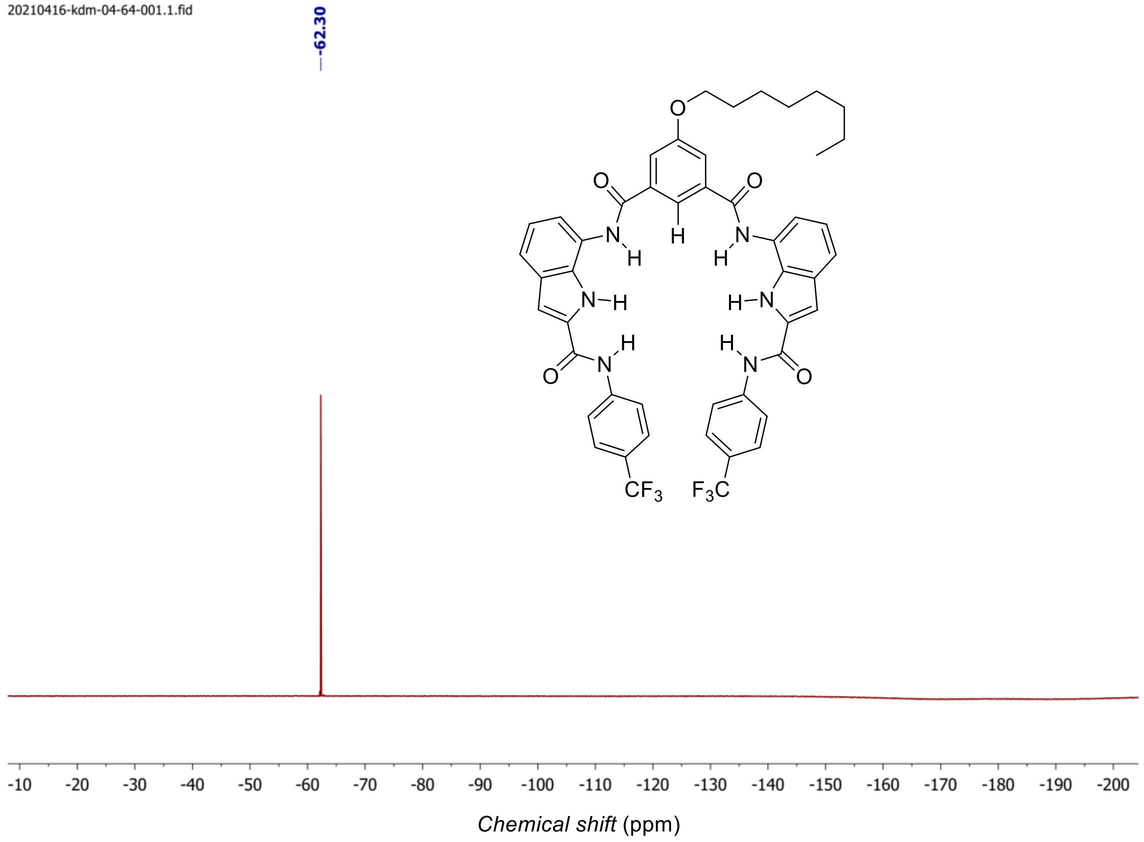

Supplementary Figure 69. ^{19}F NMR spectrum of compound **1d** in $CDCl_3$.

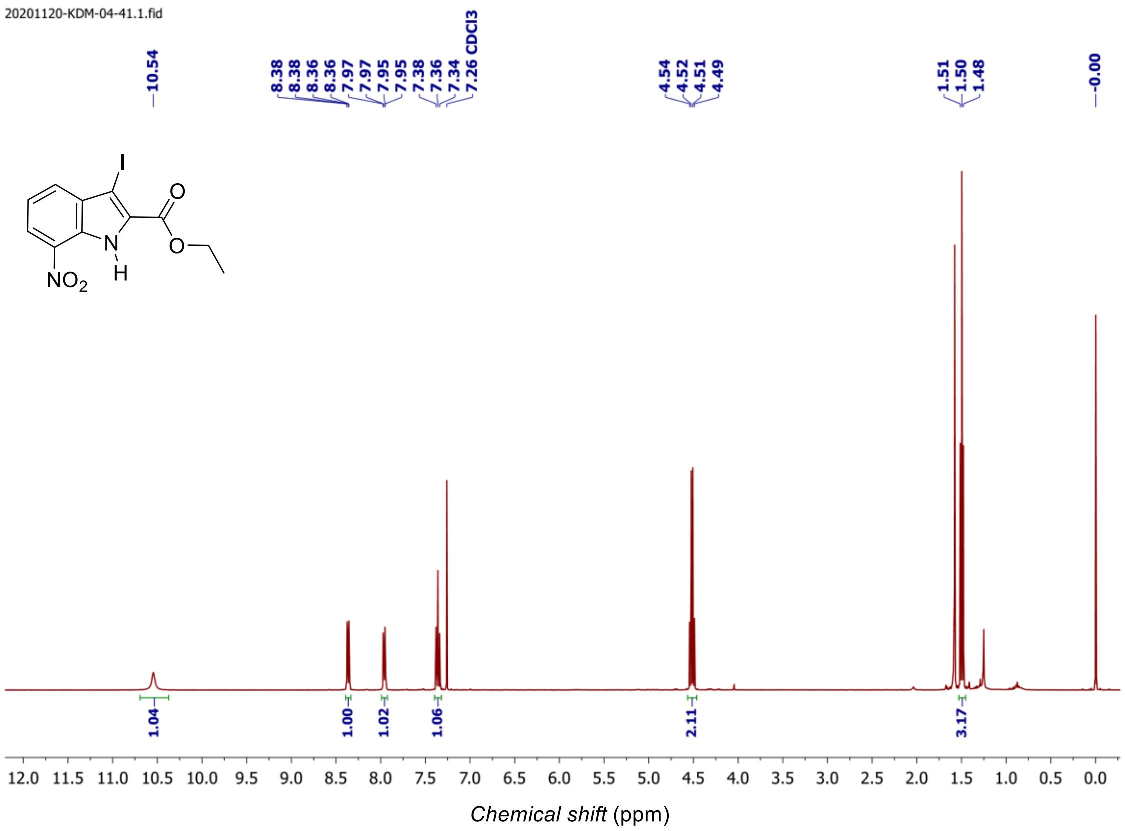

Supplementary Figure 70. ^{1}H NMR spectrum of compound **13** in $CDCl_3$.

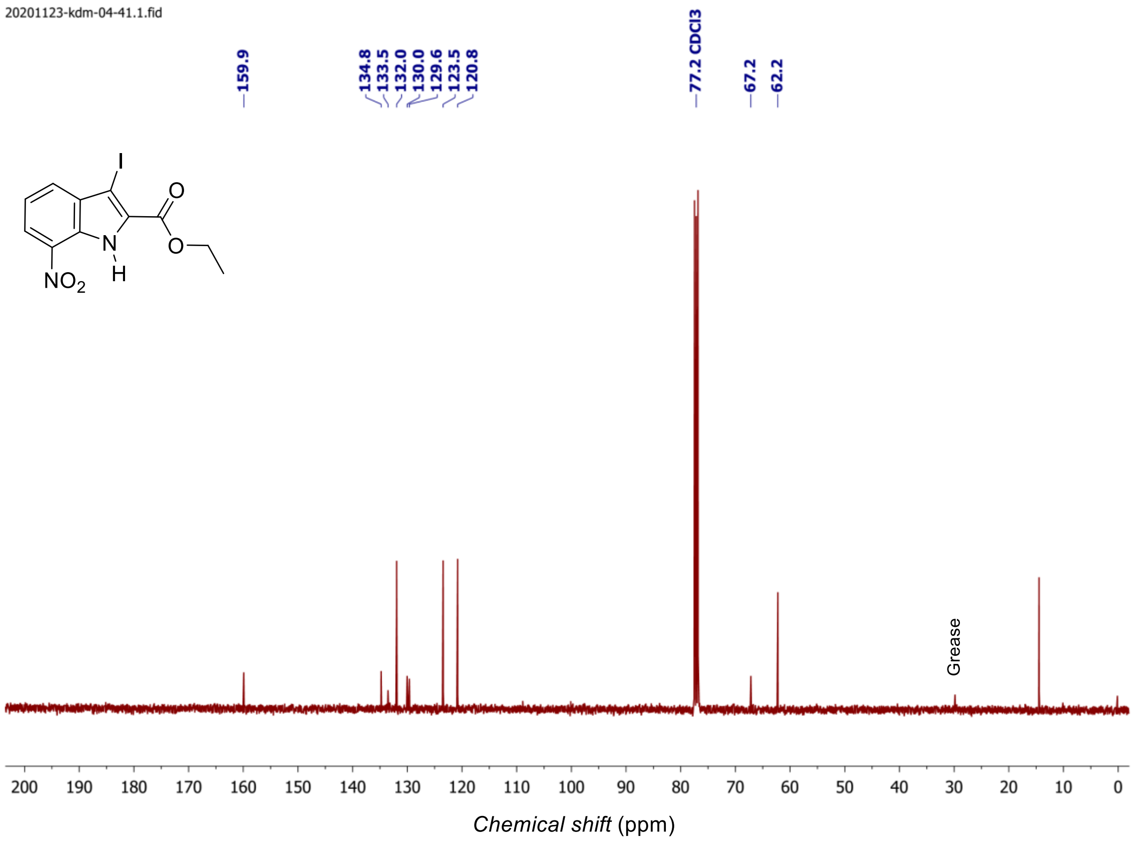

Supplementary Figure 71. ^{13}C NMR spectrum of compound **13** in $CDCl_3$.

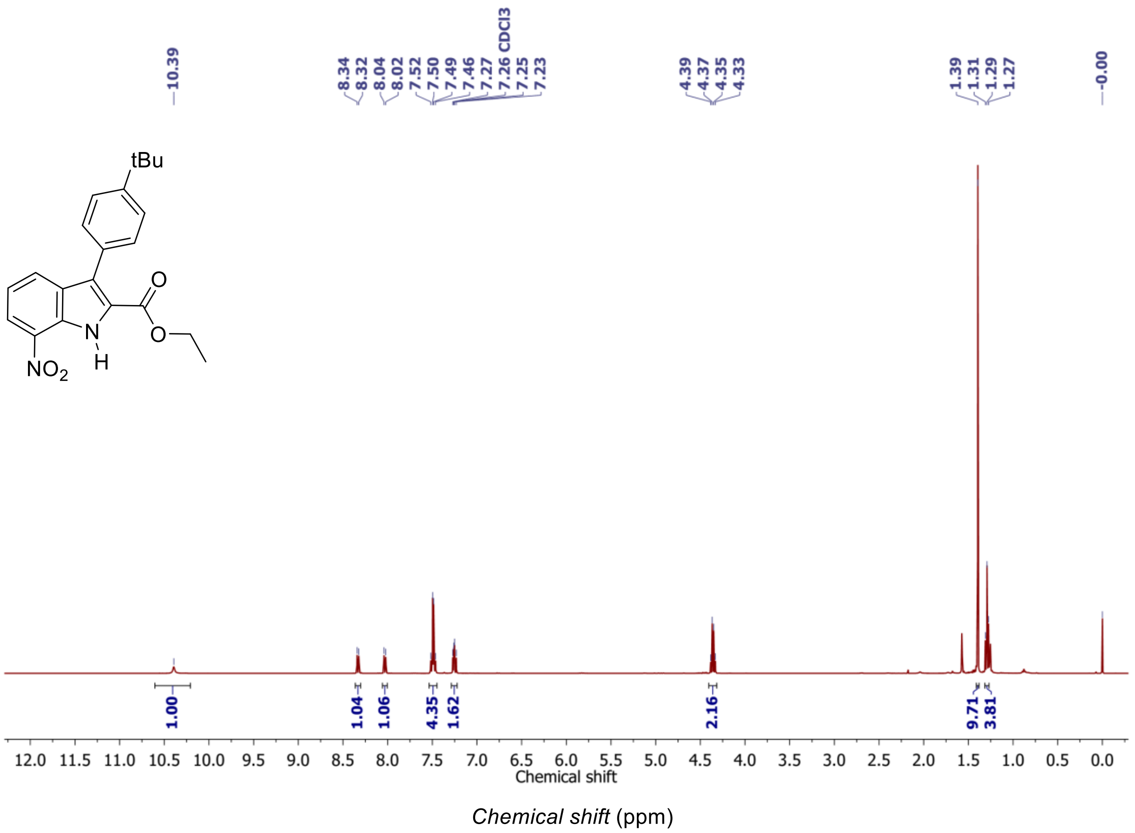

Supplementary Figure 72. ^{1}H NMR spectrum of compound **14** in $CDCl_3$.

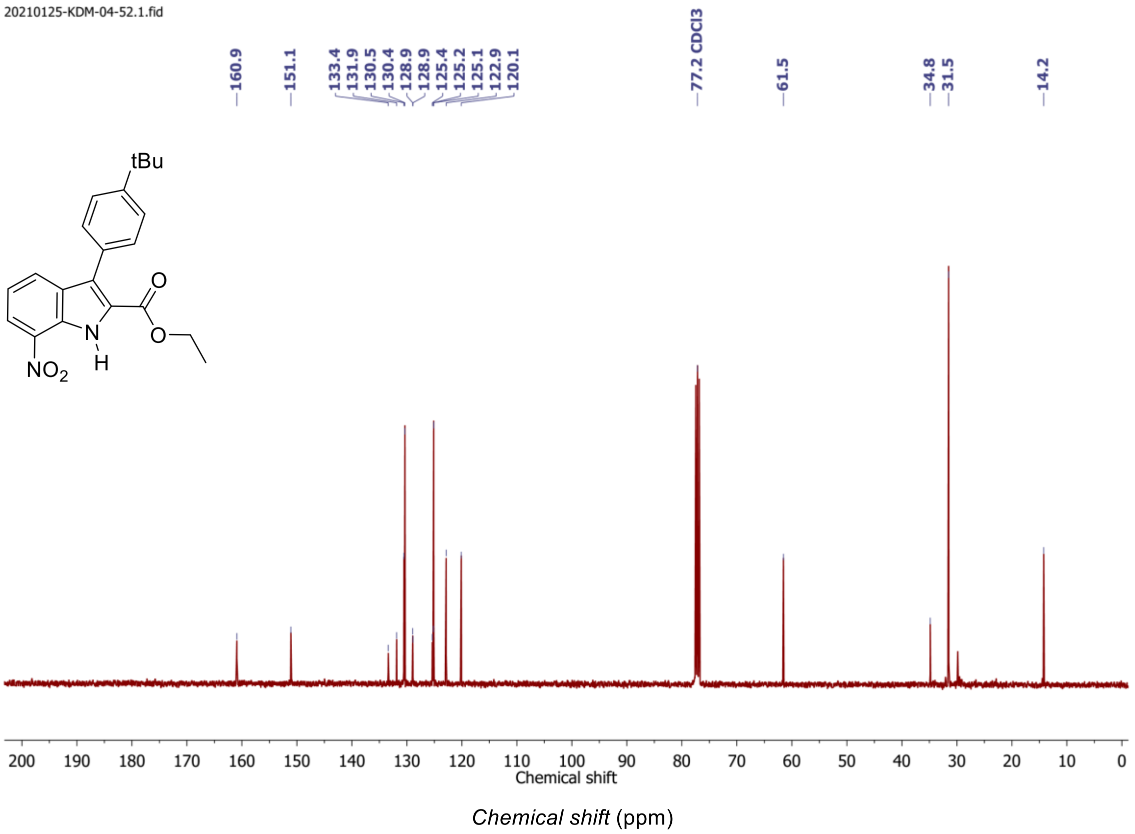

Supplementary Figure 73. ^{13}C NMR spectrum of compound **14** in $CDCl_3$.

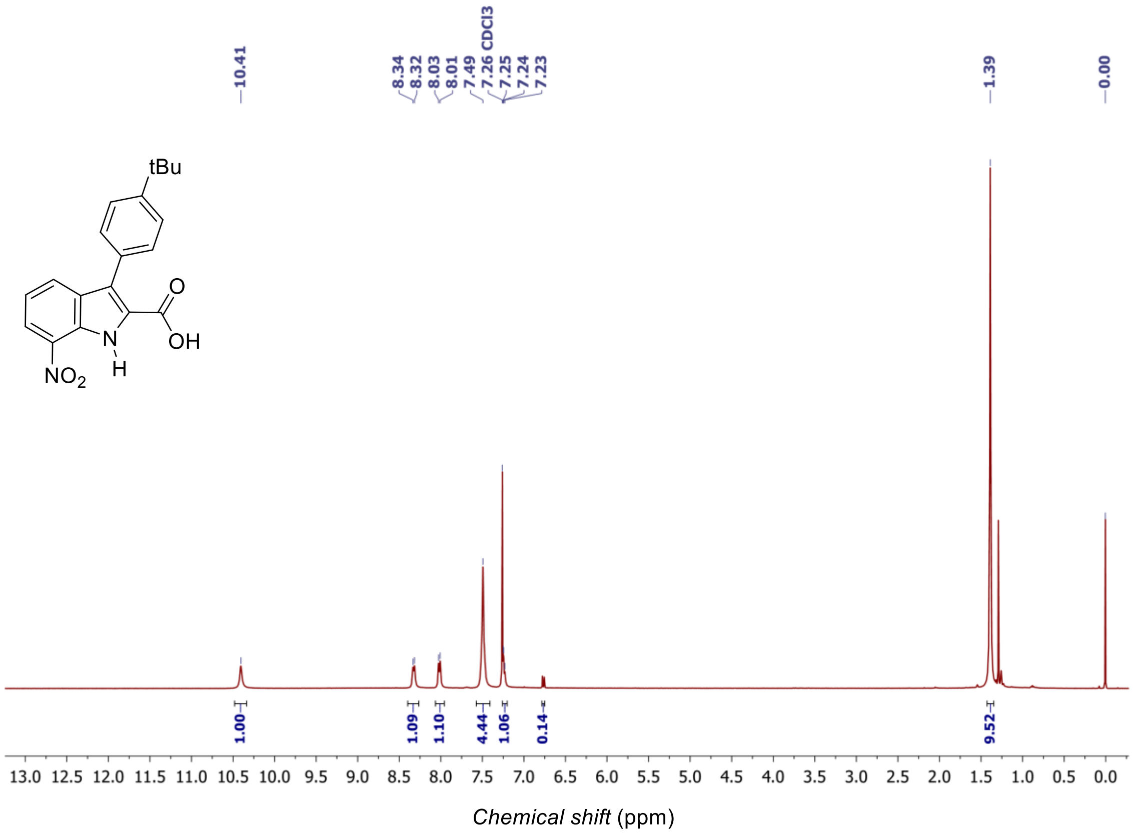

Supplementary Figure 74. ^{1}H NMR spectrum of compound **15** in $CDCl_3$.

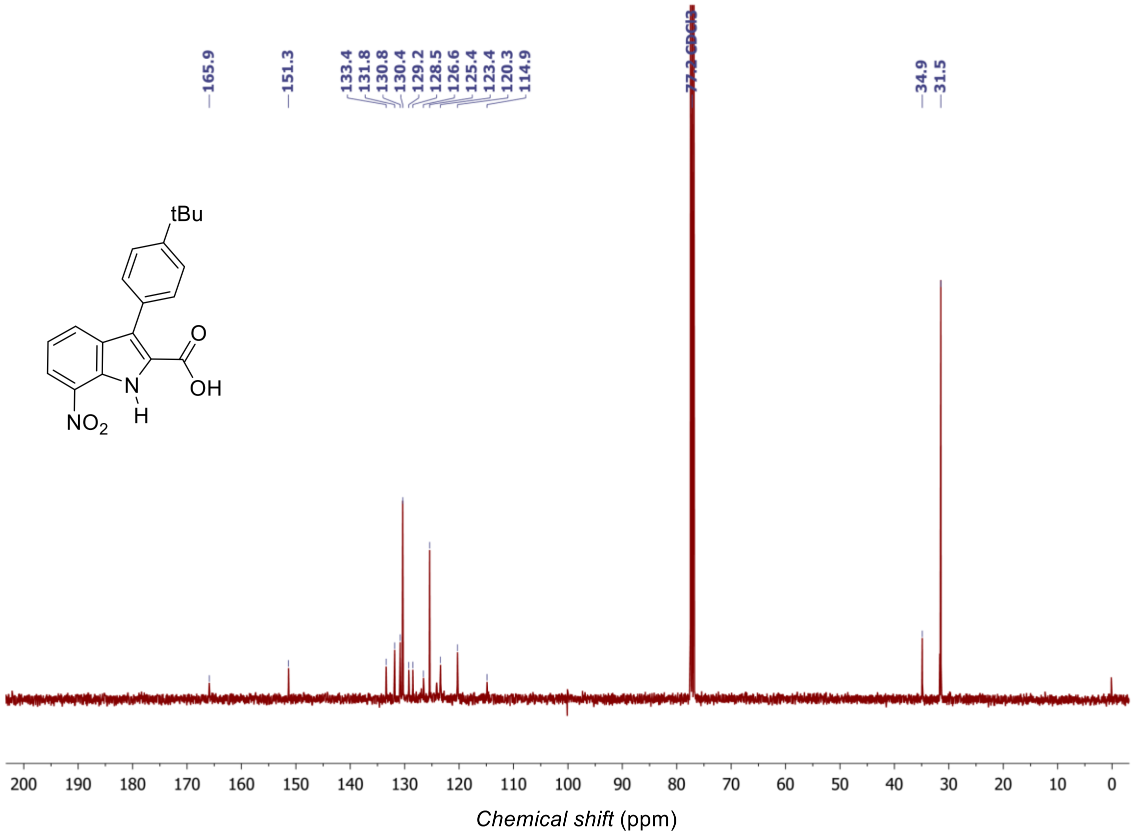

Supplementary Figure 75. ^{13}C NMR spectrum of compound **15** in $CDCl_3$.

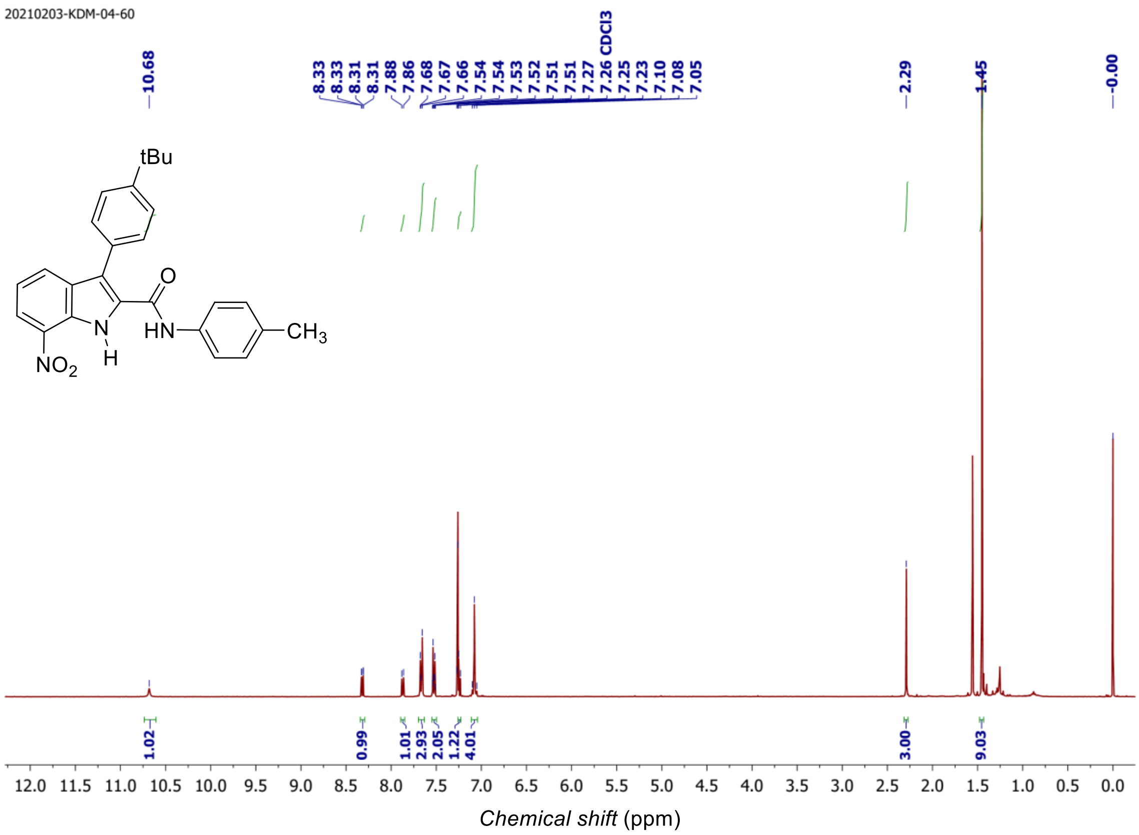

Supplementary Figure 76. ^{1}H NMR spectrum of compound **9c** in $CDCl_3$.

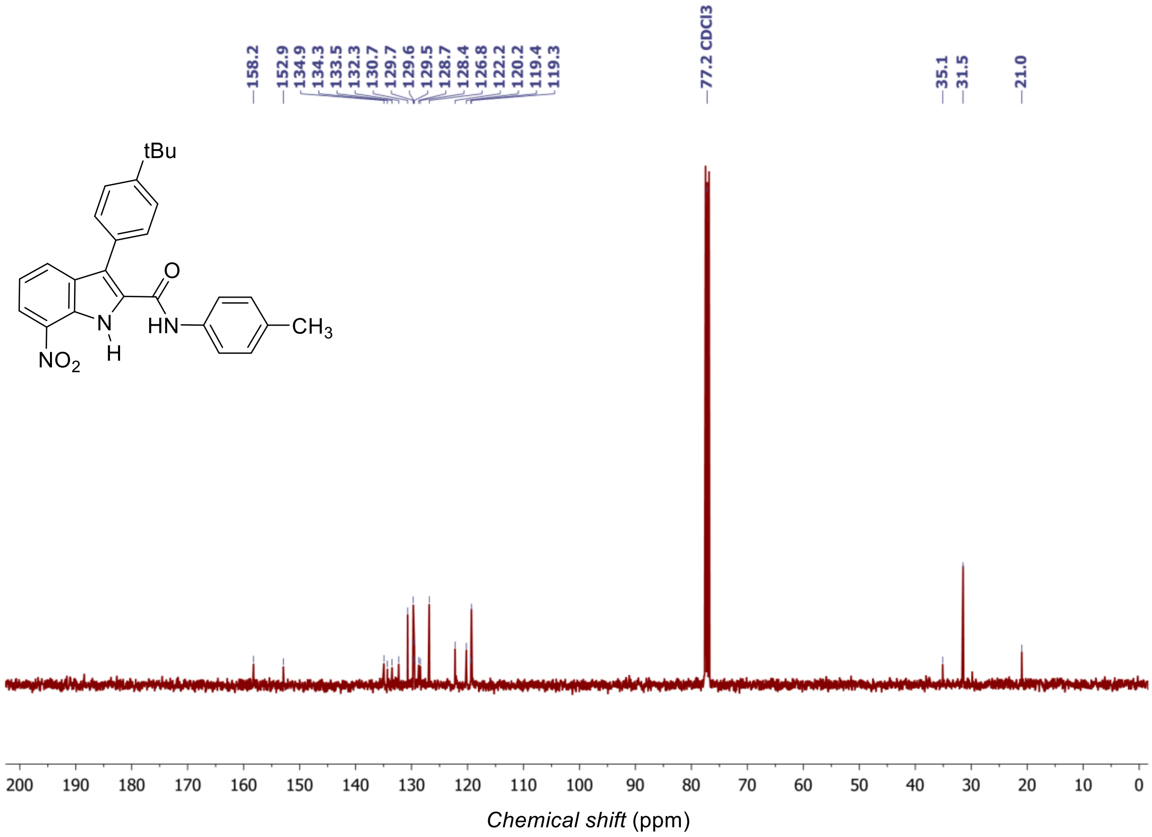

Supplementary Figure 77. ^{13}C NMR spectrum of compound **9c** in $CDCl_3$.

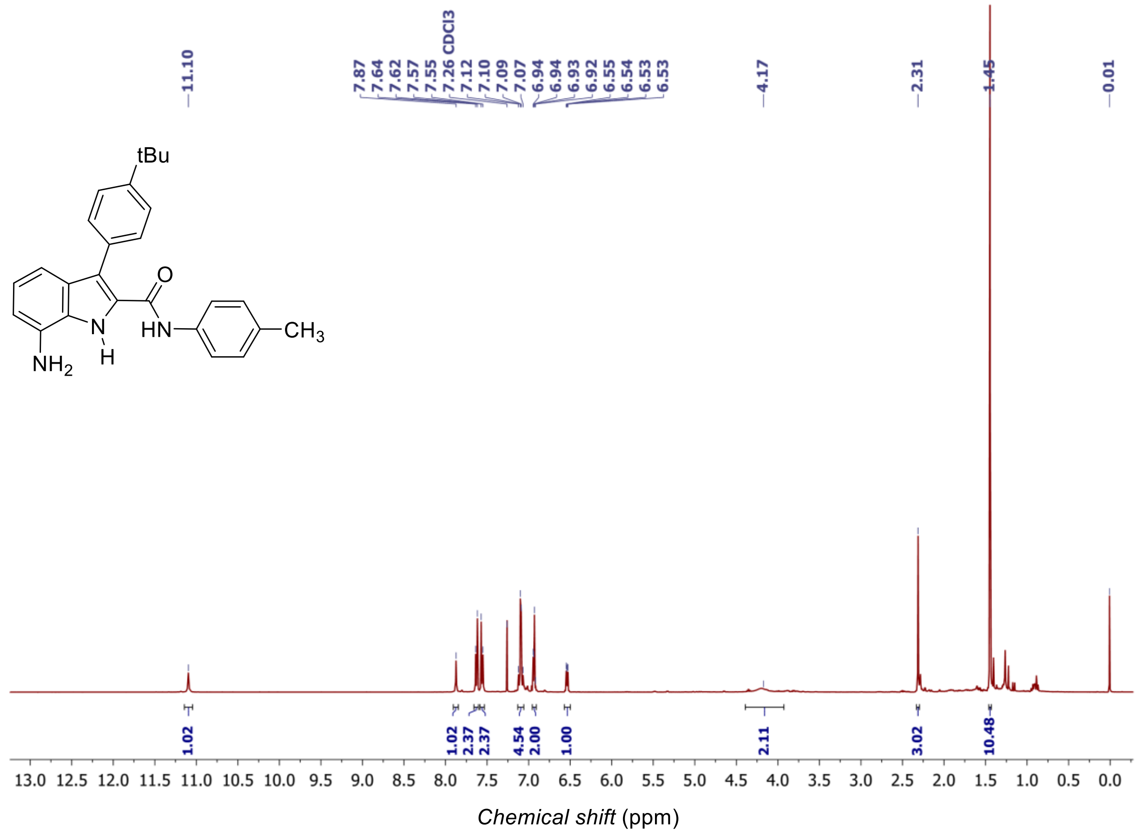

Supplementary Figure 78. ^{1}H NMR spectrum of compound **10c** in $CDCl_3$.

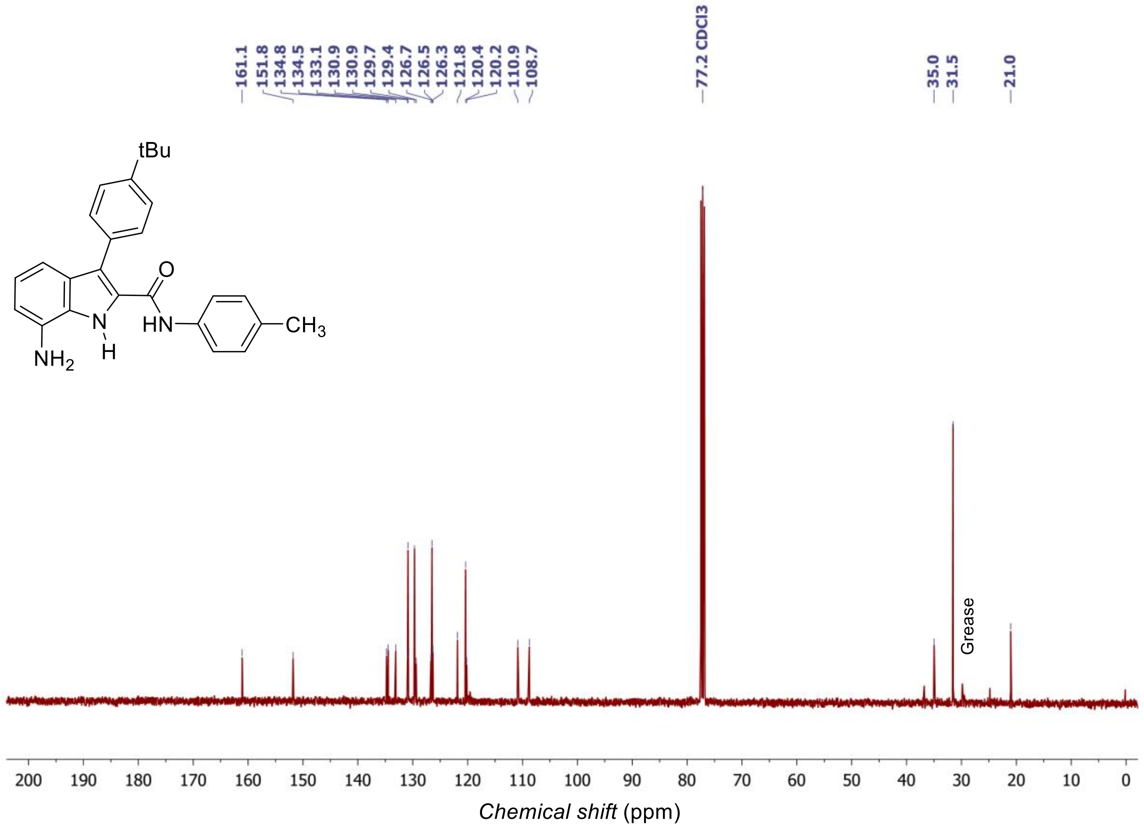

Supplementary Figure 79. ^{13}C NMR spectrum of compound **10c** in $CDCl_3$.

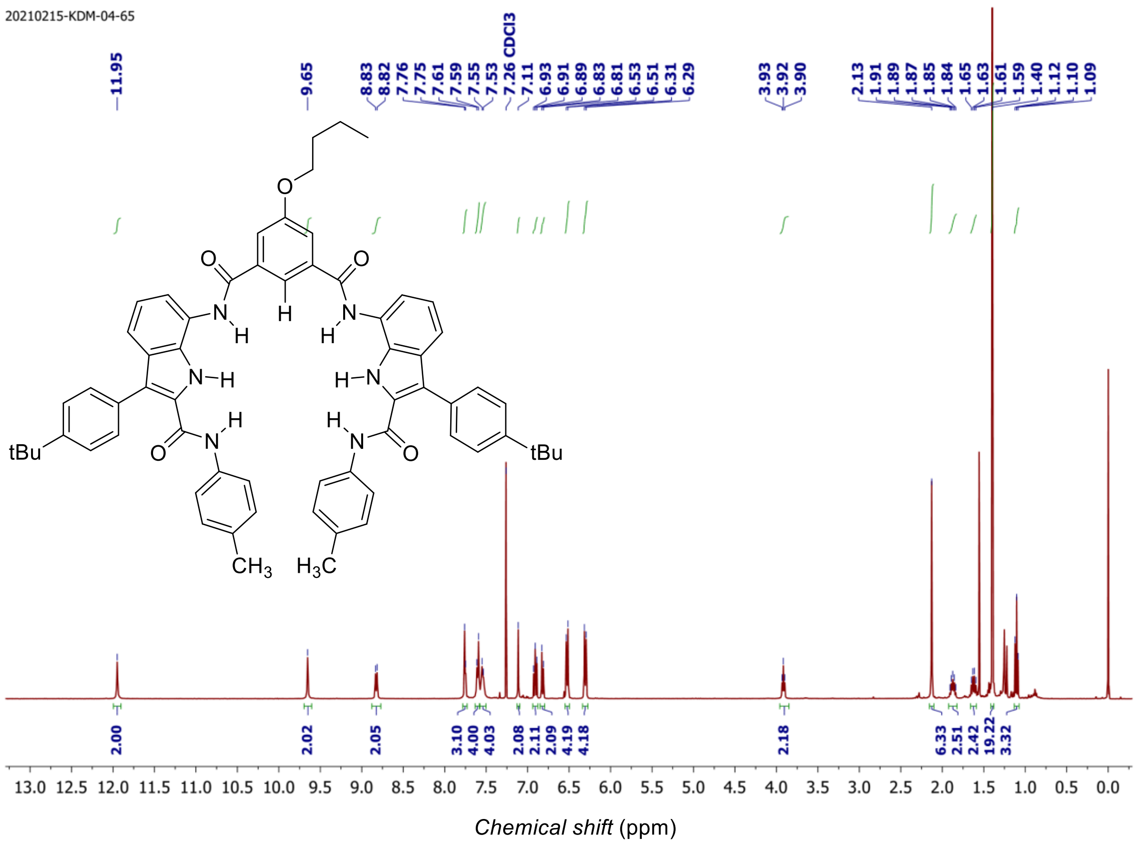

Supplementary Figure 80. ^{1}H NMR spectrum of compound **1c** in $CDCl_3$.

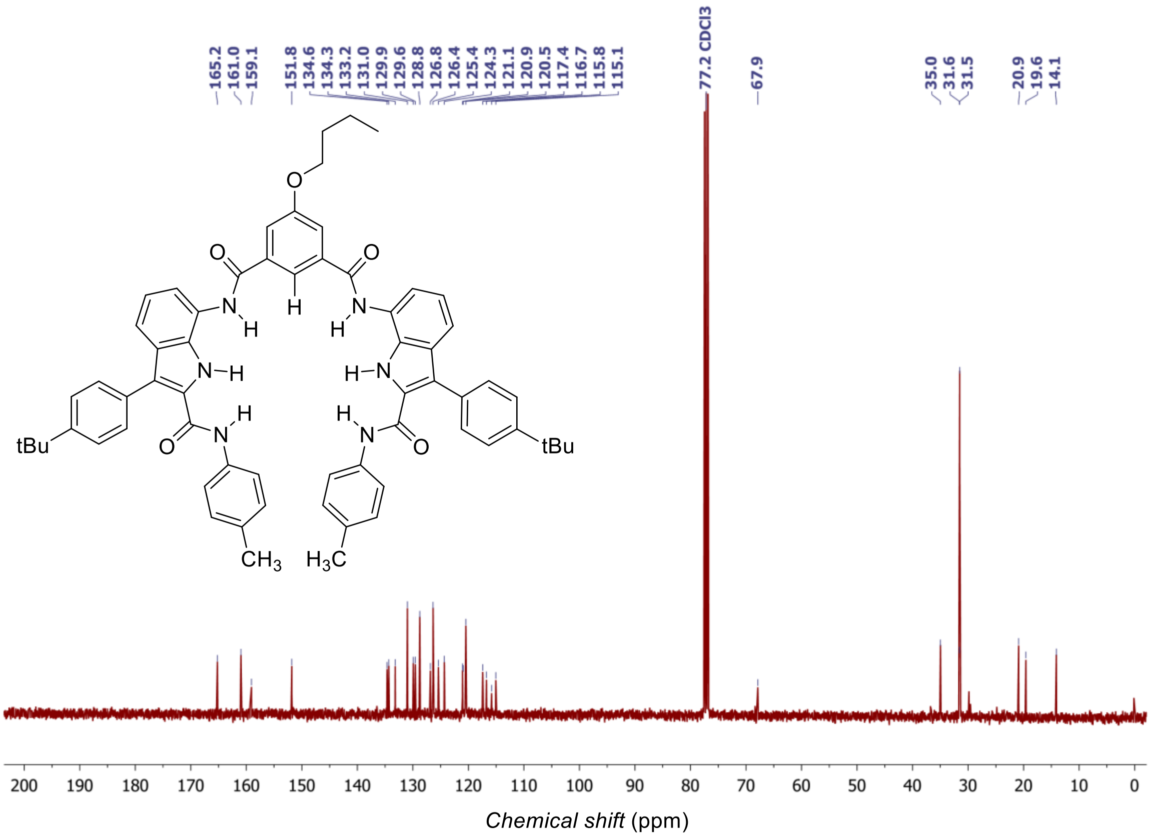

Supplementary Figure 81. ^{13}C NMR spectrum of compound **1c** in $CDCl_3$.

Supplementary References

1. Xu, R., *et al.* Design, Synthesis, Crystal Structures, and Insecticidal Activities of Eight-Membered Azabridge Neonicotinoid Analogues. *J. Agric. Food Chem.* **62**, 381–390 (2014).
2. Xiong, L., *et al.* Discovery of Potent Succinate-Ubiquinone Oxidoreductase Inhibitors via Pharmacophore-linked Fragment Virtual Screening Approach. *J. Agric. Food Chem.* **64**, 4830–4837 (2016).
3. Dolomanov, O. V., Bourhis, L. J., Gildea, R. J., Howard, J. A. K. & Puschmann, H. OLEX2: a complete structure solution, refinement and analysis program. *J. Appl. Cryst.* **42**, 339–341 (2009).
4. Sheldrick, G. SHELXT - Integrated space-group and crystal-structure determination. *Acta Cryst.* **C71**, 3–8 (2015).
5. Sheldrick, G. SHELXT - Integrated space-group and crystal-structure determination. *Acta Cryst.* **A71**, 3–8 (2015).
6. Nordstrom, L. J., Clark, C. A., Andersen, B., Champlin, S. M. & Schwinefus, J. J. Effect of Ethylene Glycol, Urea, and N-Methylated Glycines on DNA Thermal Stability: The Role of DNA Base Pair Composition and Hydration. *Biochemistry* **45**, 9604–9614 (2006).
7. Oprzeska-Zingrebe, E. A. & Smiatek, J. Preferential Binding of Urea to Single-Stranded DNA Structures: A Molecular Dynamics Study. *Biophys. J.* **114**, 1551–1562 (2018).
